# Tailor-made 3D *in vitro* maturation of early antral follicles uncovers cumulus-cell transcriptomic driver signature to predict oocyte competence

**DOI:** 10.3389/fendo.2025.1629815

**Published:** 2025-10-01

**Authors:** Camila Rojo-Fleming, Fani Konstantinidou, Alessia Peserico, Chiara Di Berardino, Giulia Capacchietti, Delia Nardinocchi, Chiara Camerano Spelta Rapini, Valentina Russo, Nicola Bernabò, Antonio Capalbo, Liborio Stuppia, Valentina Gatta, Barbara Barboni

**Affiliations:** ^1^ Department of Bioscience and Technology for Food, Agriculture and Environment, University of Teramo, Teramo, Italy; ^2^ Department of Neuroscience, Imaging and Clinical Sciences, School of Medicine and Health Sciences, “G. d’Annunzio” University of Chieti-Pescara, Chieti, Italy; ^3^ Unit of Molecular Genetics, Center for Advanced Studies and Technology (CAST), “G. d’Annunzio” University of Chieti-Pescara, Chieti, Italy

**Keywords:** cumulus cells, transcriptome, ovine oocyte competence, differentially expressed genes (DEGs), highly interconnected genes (HUBs), follicle – enclosed oocyte *in vitro* maturation (FEO-IVM), early antral follicles

## Abstract

**Background:**

Assisted reproductive technologies (ART) are constrained by the limited pool of medium antral follicles. Early antral follicles (EAfs) are an emerging additional source coming either from cryopreserved ovaries or from *in vitro* folliculogenesis protocols. The EAfs have the advantage of being gonadotropin sensitive follicles enclosing fully grown oocytes that can be enrolled in ART using advanced IVM techniques. The present research has been designed using the validated 3-D follicle-enclosed IVM protocol to insight into EAfs ART competence by profiling the transcriptome of the surrounding cumulus cells (CCs) to uncover non-invasive predictors of oocyte developmental competence.

**Results:**

Transcriptome analysis of 22–141 genes revealed 1–386 DEGs in CCs supporting metaphase-II (MII) oocytes and 1–420 in CCs enclosing germinal-vesicle (GV) oocytes. Network modelling pinpointed as signature of competent CCs three down-regulated outliers (EFHD1, HS6ST2, SLC35G1) and two hubs (CDC6, KIF11), while the unsuccessful ones revealed six outliers (HBA1, SLC39A8, ERO1A, TKDP5, CALCRL, ELOVL6) and the hub CASP3. The profile of EAfs enclosing competent oocyte converged towards cumulus expansion and controlled cell-cycle quiescence pathways whereas lipid dysregulation, oxidative stress and apoptosis characterized CC surrounding incompetent oocytes. The endpoint comparison yielded 11 markers, highlighted by SEMA3A up-regulation and IL1A, DDIT4 and VNN1 down-regulation. qPCR confirmed the transcriptome profile of the key genes (HS6ST2, CDC6, ERO1, CASP3 and SEMA3A) while immuno-assays corroborated the differential expression and localization of some related codified driver proteins (HAS2, CASP3 and SEMA3A).

**Conclusions:**

CC transcriptomics defines a concise 12-gene driver panel plus 11 endpoint markers that accurately predict the maturation fate of individual EAfs by providing actionable targets and a practical basis for rapid, non-invasive selection of high-quality follicles, with potential to enhance fertility preservation, livestock breeding and clinical ART outcomes. In addition, the analysis point on new gene/protein mediating CCs maturation mechanism is to be considered.

## 1 Introduction

Assisted Reproductive Technologies (ART) have transformed clinical and veterinary management of infertility, yet they still exploit only a narrow fraction of the ovarian follicular reserve. Conventional stimulation protocols mainly recruit fully−grown, pre−ovulatory follicles whose oocytes are readily accessible, but whose numbers are intrinsically limited—an issue that becomes critical in women with diminished ovarian reserve or advanced age and in species where recovery of large follicles is impractical.

Early antral follicles (EAfs)- follicles located at the transition between pre−antral and true antral stages-outnumber later stage follicles by orders of magnitude (1–5). They can be collected directly *in vivo* or generated *in vitro* as the end−point of multi−step folliculogenesis protocols in several mammals (mouse (6, 7), sheep (4, 8–11), cattle (12), buffalo (13), goat (14–17). Although EAfs contain oocytes that have not completed growth, these oocytes are actively undergoing chromatin remodeling, epigenetic reprogramming and telomere elongation—processes tightly linked to future developmental competence (18–21). Leveraging EAfs therefore offers a powerful avenue to enlarge the pool of fertilizable gametes for fertility preservation, livestock improvement and conservation programs.

A peculiar advantage of EAfs is their sensitivity to luteinizing−hormone (LH) signals despite their immature size. Human chorionic gonadotropin (hCG), which exhibits strong LH−mimetic and residual FSH−like activity, has proven essential for inducing their meiotic resumption and cytoplasmic maturation in the Follicle−Enclosed Oocyte (FEO) culture system (1, 2). Specifically, experimental evidence shows that EAfs fail to progress beyond the germinal−vesicle stage in the absence of hCG, whereas a single hCG stimulus supports both nuclear maturation and oocyte quality without the need for additional FSH supplementation (1). Nonetheless, only a minority of EAfs respond optimally in terms of developmental competence if compared with those from advanced stage follicles, underscoring the need to specialize *in-vitro* maturation (IVM) approaches.

Traditional IVM relies on isolated cumulus–oocyte complexes (COCs) and has been thoroughly optimized for cattle and sheep (22–26). In contrast, the FEO approach maintains the three−dimensional follicular architecture, allowing a more physiological diffusion of gonadotropins and local growth factors. Recent bio−inspired 3D scaffolds have markedly improved maturation rates of EAf−derived oocytes over conventional COC−IVM (1, 2), but competence still lags behind that of oocytes obtained from larger antral follicles.

Progress in EAf−based ART is further hampered by the absence of reliable, non−invasive biomarkers that could predict oocyte developmental competence before fertilization. Because sampling the oocyte itself compromises viability, attention has shifted toward the surrounding somatic compartment. Cumulus cells (CCs), linked to the oocyte via trans−zonal projections and gap junctions, mirror the metabolic and signaling milieu that supports oocyte maturation, making them prime candidates for molecular assessment.

The present study employs a genome−wide transcriptomic analysis of CCs collected from EAfs cultured in a 3D FEO system. By comparing CC profiles associated with oocytes arrested at the germinal−vesicle stage to those surrounding metaphase−II oocytes after hCG stimulation, we seek to identify differentially expressed genes that could serve as predictive biomarkers of meiotic and developmental competence. These insights are expected to refine follicle selection, optimize IVM protocols and ultimately expand the pool of high−quality oocytes available for ART in both human and animal reproduction.

## 2 Materials and methods

### 2.1 Ovary collection

Ovaries from prepubertal (approximately 5-month-old) *Appenninica* lambs were collected post – mortem from a local slaughterhouse as discarded material. Transported under temperature-controlled conditions (<1 hour), the tissues were rinsed multiple times with a 0.9% NaCl solution supplemented with 1 mg/mL Benzoxonium chloride (Bialcol #032186013, Vemedia Pharma). After medulla removal, ovaries were placed in HEPES-buffered TCM199 medium (#M7528, Sigma Aldrich) and sectioned into 0.5 × 0.5 × 0.5 cm cortical fragments.

### 2.2 Ovarian surface epithelium cell collection for follicle-enclosed oocyte coculture system

Ovarian surface epithelial (OSE) cells were isolated according to Peserico et al. (14). More in detail, OSE were recovered from prepubertal ovarian cortex using a surgical scalpel after 5 min incubation in 0.25% Trypsin/EDTA solution (200 mg/L) at 38.5°C. The resulting cell suspensions were transferred to a 6 cm Petri dish containing DPBS supplemented with 30% FBS (10270-106, Gibco) to neutralize Trypsin(25200056, Gibco). Following centrifugation, OSE cells were resuspended in alphaMEM (#BE02-002F, Lonza), 20% FBS (#10270-106, Gibco), 1% glutamine (BE17-605E/U1, Lonza), and antibiotics, including 75 mg/L penicillin-G and 50 mg/L streptomycin sulfate (#DE17-602E, Lonza). OSE identity was confirmed according to Nakamura et al. (27). After one passage, cells were seeded onto transwells to establish a feeder cell monolayer for FEO coculture.

### 2.3 FEO *in vitro* maturation from EAfs

FEO IVM of EAfs was performed as previously described (14). Briefly, EAfs were isolated from ovarian cortical fragments using 32 G needles, under sterile conditions. Follicles were selected based on size (360 ± 31 μm), morphology, and theca integrity. Prior to culture, follicles were screened under an inverted-phase microscope with time-lapse imaging to assess diameter and signs of degeneration. EAfs were matured in transwell systems in 96-well plates, with scaffolds of PCL-patterned electro spun materials and a monolayer of ovarian surface epithelial (OSE) cells at the bottom, using alphaMEM, 20% FBS, 1% glutamine, antibiotics- based medium supplemented with 25 IU/mL hCG and incubated at 38.5°C with 5% CO_2_. The poly(ϵ-caprolactone) (PCL) patterned material was validated in our previous studies for its ability to create a structured containment grid that prevents follicular collapse and cell adhesion, thereby preserving long-term 3D follicle architecture (15, 28). In addition, the co-culture with OSE cells contributes to recreating the ovarian cortex cellular assembly by reproducing the paracrine microenvironment of ovarian cortex (14). After 24 h IVM, FEO was opened to isolate CCs. CCs were then selected based on the nuclear stage of the oocyte; CCs from EAfs before hCG stimulation were collected as a control group at the germinal vesicle (GV) stage.

### 2.4 Oocyte nuclear stage assessment and CCs collection

Following *in vitro* maturation, oocytes were isolated from their surrounding cumulus cells, stained with DAPI, and analyzed using NIS-Elements time-lapse imaging software (Eclipse Ti Series, Nikon, Tokyo, Japan) to determine their meiotic nuclear stage. Oocytes were classified into different stages, including Germinal Vesicle (GV) and Metaphase II (MII), as previously described (13, 29).

### 2.5 Microarray transcriptomic analysis

The workflow for the microarray analyses follows the approach already validated in our previous study (29). Specifically, total RNA was extracted from pools of five CCs using the Single-Cell RNA Purification Kit (#51800, Norgen Biotek Corp) per the manufacturer’s protocol. Transcriptomic profiling was performed with the MicroArray GeneChip System (Applied Biosystems). For microarray analysis, 100 ng of RNA was used to synthesize complementary DNA (cDNA) using the GeneChip WT PLUS Reagent Kit (Applied Biosystems). cRNA was generated via *in vitro* transcription, purified, reverse-transcribed, and converted into single-stranded cDNA (ss-cDNA), which was then fragmented and labeled. Hybridization was performed with GeneChip™ Hybridization, Wash, and Stain Kits using OviGene 1.0 ST GeneChip Arrays (Ovine; Applied Biosystems) in the GeneChip Hybridization Oven 645 and Fluidics Station. Scanning was done with the GeneChip Scanner 3000 7G, and analysis was conducted using Affymetrix Command Console (AGCC) software. Raw data were processed with Transcriptome Analysis Console (TAC) software (ver. 4.0.2, Applied Biosystems). Normalization followed the SST-RMA algorithm, with a DABG cutoff of 0.05 and an AUC threshold ≥0.7. Differentially expressed genes (DEGs) were identified using a p-value < 0.05 (one-way ANOVA) and a fold change >2 for upregulation or < -2 for downregulation.

### 2.6 Network creation, visualization and analysis

Three datasets (designated as pairwise 1, 2, and 3) were generated using the Transcriptome Analysis Console (TAC) 4.0 Software (Applied Biosystems). Each data set served as an input for constructing a network using Cytoscape 3.10.2 (http://www.cytoscape.org). The resulting networks were analyzed using the Network Analyzer plug-in in Cytoscape, following previously described methods (30, 31). The analysis included the computation of key topological parameters such as the number of nodes, number of edges, average number of neighbors, network diameter, characteristic path length, clustering coefficient, and connected components (**Supplementary Datasheet 1**). Additional network- and node-level analyses are detailed in the subsequent sub-sections.

#### 2.6.1 Identification of highly interconnected regions using MCODE

The Cytoscape plugin Molecular Complex Detection (MCODE) (28) was utilized to identify and analyze clusters of densely interconnected nodes (modules) within the network. Modules with k-core values greater than 5 and node degrees exceeding 5 were selected for further KEGG pathway analysis, which was conducted using the Cytoscape ClueGO plugin. The reference database used for network construction and pathway analysis was Ovis aries (Taxid:9940).

#### 2.6.2 Identification of drivers within network modules´

The study distinguished two types of drivers: highly modulated DEG and highly interconnected DEGs (HUBs). Highly modulated DEGs, identified as outlier genes based on their expression levels, were detected by comparing gene expression values applying the Interquartile Range Method (32) and visualized using a Volcano Plot (GraphPad Prism 10.1.1; https://www.graphpad.com/). Of note, a significance threshold of p < 0.05 was applied to determine whether a gene’s expression significantly deviated from the expected Gaussian distribution of non-outliers.

HUBs were identified using the CytoHubba plugin in Cytoscape (33) by following a four-step workflow according to recent literature methodological evidence (22–26, 34, 35) (29).

- First, 12 centrality coefficients supported by CytoHubba (**Supplementary Table 1**), were calculated for each DEG, providing each DEG with a score.
- DEGs were then ranked based on the scores of these centrality coefficients (II).
- The top 10 DEGs for each centrality coefficient (those with the highest scores) were selected (III).
- and DEGs appearing in the top 10 for at least 5 of the 12 algorithms were identified as hubs (IV).

#### 2.6.3 Venn diagram

A Venn diagram tool (https://bioinformatics.psb.ugent.be/webtools/Venn/) was used to illustrate the shared and signature DEGs across the networks.

#### 2.6.4 Microarray validation

The validation of the microarray data was conducted through the application of qPCR, Immunofluorescence and immunoblotting as follow.

### 2.7 Protein localization using immunofluorescence

EAfs enclosing competent and incompetent oocytes were collected in PBS and fixed with paraformaldehyde for 1 hour. After washing three times with PBS, samples were incubated overnight at 4°C. For embedding, EAfs were dehydrated through graded ethanol (70%, 90%, 95%, 100%) and Xylene, followed by infiltration in molten Paraplast (56-58°C) overnight. After solidification, samples were sectioned at 5 µm thickness. Sections were deparaffinized in Xylene (2 × 5 minutes), rehydrated through ethanol and distilled water, and washed in PBS. Slides were incubated with Hoechst 33342 (1:1000 in DPBS; #62249; Thermo Fisher Scientific, Waltham, MA, USA) for 10 minutes to stain nuclei. Primary antibodies were incubated overnight at 4°C under the following conditions: anti-HAS2 antibody (1:600 dilution in 1% BSA in PBS; #AB140671, Abcam), anti-CASP3 antibody (1:1000 dilution in PBS containing 0.05% Tween-20; #9662S, Cell Signaling Technology), and anti-SEMA3A antibody (1:250 dilution in 1% BSA in PBS with 0.05% Tween-20; #PAB7888, Abnova). After washing, samples were incubated with the appropriate secondary antibodies for 1 hour at room temperature: anti-mouse Cy3-conjugated antibody (1:100 in PBS, Alexa Fluor 594) for HAS2, and anti-rabbit IgG Alexa Fluor 488-conjugated antibody (1:100 in PBS) for CASP3 and SEMA3A. After washing, imaging was performed using a Nikon confocal microscope (Nikon Arl, Düsseldorf, Germany) equipped with NIS-Element software 4.40 (Nikon, Düsseldorf, Germany). In all experiments, non-immune serum was used in place of the primary antisera as a negative control according to Russo et al. (36). All controls performed were negative.

### 2.8 Protein expression quantification through immunoblotting

EAf cumulus cell extracts were prepared in RIPA buffer (R0278, Sigma-Aldrich, St. Louis, MO, USA), supplemented with protease inhibitors (P2714, Sigma-Aldrich, St. Louis, MO, USA) and phosphatase inhibitors (P5726, Sigma-Aldrich, St. Louis, MO, USA), incubated on ice for 30 minutes and then centrifuged at 12,000× g for 10 minutes at 4°C. Protein concentration was determined using the Quick Start™ Bradford 1x Dye Reagent (Bio-Rad Laboratories, Hercules, CA, USA). Total protein from each sample (20 μg) were denatured in Laemmli buffer, run on SDS-PAGE, and analyzed by immunoblotting using HRP-conjugated secondary antibodies used were mouse (sc-516102, Santa Cruz Biotechnology, Dallas, TX, USA), rabbit (sc-2357, Santa Cruz Biotechnology, Dallas, TX, USA), and goat (sc-2354, Santa Cruz Biotechnology, Dallas, TX, USA). Chemiluminescent signals on the membranes were detected using the ChemiDoc™ MP Imaging System (Bio-Rad Laboratories, Milan, Italy). Densitometric analysis for protein quantification was conducted using ImageLab software analyzer (ImageLab v. 6.1.0 Bio-Rad Laboratories, Milan, Italy). Primary antibodies against *HAS2* (1:1000 in T-TBS 0.1% + 5% BSA; #Ab140671, Abcam), *SEMA3A* (1:1000 in T-TBS 0.1% + 5% BSA; #PAB7888, Abnova) and *CASP3* (1:1000 in T-TBS 0.1% + 5% BSA; #9662S, Cell Signaling Technology) were used.

### 2.9 Transcript quantification using RT – qPCR

Total RNA was extracted using the Single-Cell RNA Purification Kit (#51800, Norgen Biotek Corp.). A total of 1 μg of RNA was reverse transcribed with oligodT primers (Bioline) and Tetro Reverse Transcriptase (Bioline), following the manufacturer’s protocol. qPCR was performed in triplicate using the SensiFAST SYBR Lo-ROX kit (Bioline) on a QuantStudio3 System (Life Technologies). The following PCR conditions were applied for all experiments: 95°C for 10 minutes, followed by 40 cycles of 95°C for 10 seconds and 60°C for 30 seconds. Relative quantification was calculated using the ΔΔCt method. *GAPDH* (Glyceraldehyde 3-phosphate dehydrogenase) and *YWHAZ* (Tyrosine 3-Monooxygenase/Tryptophan 5-Monooxygenase Activation Protein Zeta) were chosen as reference genes for gene quantification. Primer sequences were selected for one highly modulated DEG and one hub gene per network, as well as one representative gene for pairwise 3: (Network 1: highly modulated DEG *HAS2*, hub *CDC6*; Network 2: highly modulated DEG *ERO1*, hub *CASP3*; pairwise 3: DEG *SEMA3A*). The forward and reverse primer sequences are provided in **Supplementary Datasheet 2**.

### 2.10 Statistical analysis

Three independent biological replicates were conducted. The statistical analysis for the Microarray GeneChip assays was performed through One-Way ANOVA followed by an empirical Bayes correction for differential expressions by the TAC software. Benjamini–Hochberg correction was applied for multiple hypothesis testing, and the adjusted values were used for all subsequent analyses. GraphPad Prism 10.1.1 (https://www.graphpad.com/) was used for all statistical analyses, with p-values less than 0.05 considered statistically significant.

## 3 Results

This study analyzed the transcriptome of EAfs induced to mature *in vitro* via hCG stimulation, comparing CCs that support oocyte maturation with those that do not. The goal was to gain single-follicle insights into the competence of EAfs releasing mature or immature oocytes as potential targets for ARTs. IVM was performed using a 3D Follicle-Enclosed Oocyte (FEO) system, which more accurately replicates intrafollicular mechanisms of meiosis resumption. At the end of IVM, follicles were classified based on the nuclear stage and developmental potential of their oocytes. This classification allowed a comparative transcriptomic analysis between CCs from two groups: those surrounding incompetent GV oocytes (unsuccessful EAfs) and those enclosing competent MII oocytes capable of parthenogenetic activation (competent follicles) (**Supplementary Datasheet 3**).

### 3.1 Transcriptome comparison between competent and incompetent of cumulus cells

**Figure 1:**
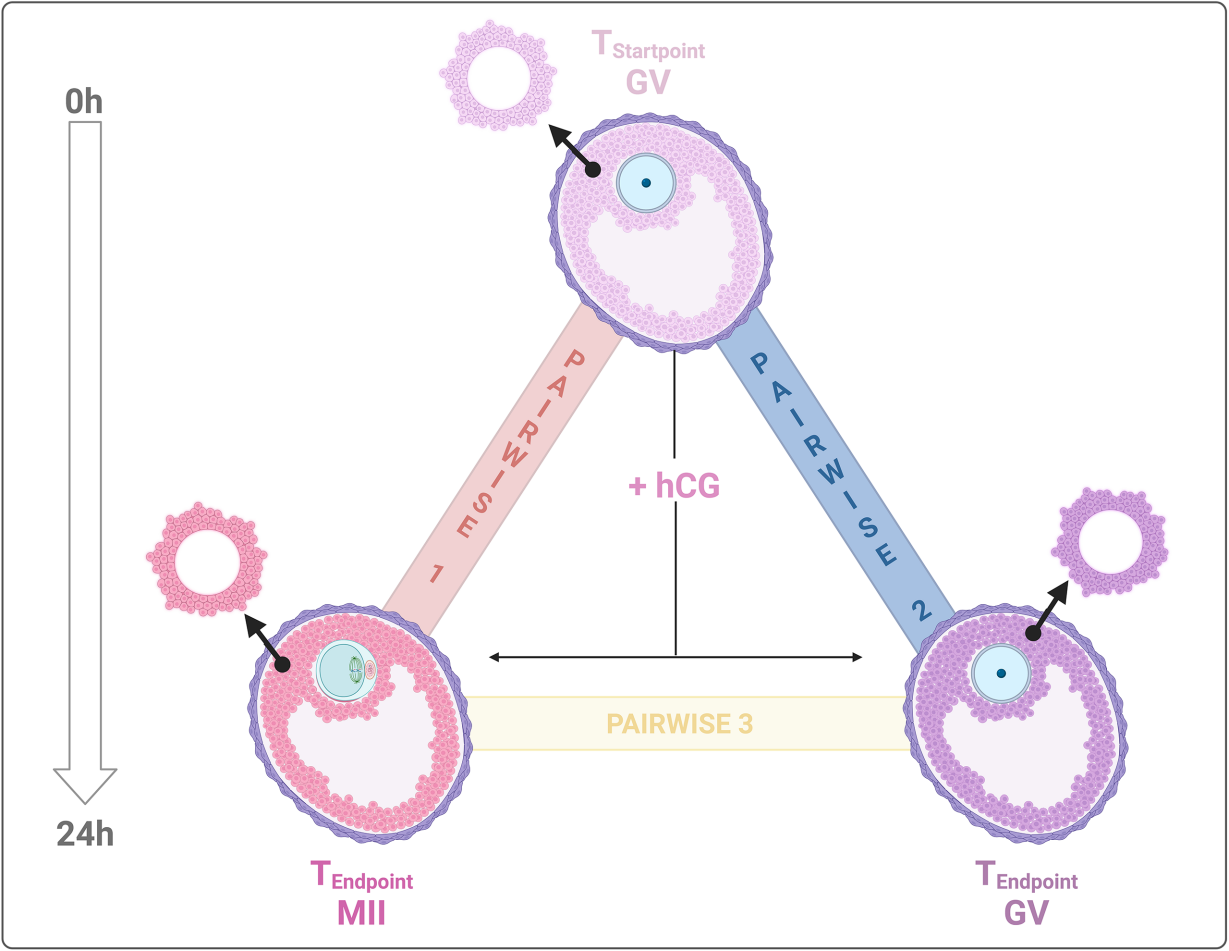
Experimental transcriptome landscape comparisons. Biological samples for transcriptome analysis were collected from: 1. CCs surrounding incompetent (GV) and competent (MII) oocytes after IVM on FEO systema and, 2. CCs from immature EAfs (GV_Startpoint_). The analysis aimed to: AIM 1 – compare CC transcriptomes pre- and post-IVM (MII *vs*. GV); and AIM 2 – compare CC transcriptomes from competent (MII) vs, incompetent (GV) EAfs post – IVM. Three pairwise comparisons were generated: Pairwise 1, MII_Endpoint_-GV_Startpoint_ indicated with red line; Pairwise 2, GV_Endpoint_-GV_Startpoint_ represented by blue and, pairwise 3, MII_Endpoint_-GV_Endpoint_ is described by a yellow line. Created with BioRender.com.

The CC transcriptomes were categorized into three datasets for comparative analysis: Pairwise 1: CCs releasing competent oocyte (MII_Endpoint_) *vs* unstimulated EAfs (before hCG: GV_Startpoint_); Pairwise 2: Cumulus cells surrounding incompetent (GV_Endpoint_) *vs* unstimulated EAfs (GV_Startpoint_); Pairwise 3: Cumulus cells releasing competent oocytes (MII_Endpoint_) *vs* cumulus cells enclosing incompetent EAfs (GV_Endpoint_). These three pairwise comparisons were analyzed to address two primary biological objectives (**Figure 1**): AIM 1: Identify the CC driver genes of follicles involved in maturation (MII_Endpoint_ *vs* GV_Startpoint_) and those linked to its failure (GV_Endpoint_ *vs* GV_Startpoint_). AIM 2: Differentiate the driver genes of CCs that enclose competent oocytes *vs* incompetent EAfs (MII _Endpoint_ *vs* GV_Endpoint_). **Table 1** summarizes the differentially expressed genes (DEGs) identified from the analysis of 22,141 *Ovis aries* transcripts. Across all pairwise comparisons, the majority of DEGs turned out to be downregulated, with rates of 54%, 59.3%, and 84.6% in Pairwise 1, 2, and 3, respectively. The complete list of DEGs is available in **Supplementary Table 2**.

**Table 1:** Differentially expressed genes (DEGs) across three pairwise comparisons.

Denomination of Pairwises	*Total number of genes*	*Genes passed filter criteria (DEG)*	*Genes with no changes*	*Upregulated*	*Downregulated*
*Pairwise 1* *(MII_Endpoint –_ GV_Startpoint_)*	22141	1386 (6.3%)	20755 (93.7%)	644 (46%)	742 (54%)
*Pairwise 2* *(GV_Endpoint –_ GV_Startpoint_)*	22141	1420 (6.4%)	20715 (93.6%)	579 (40.7%)	841 (59.3%)
*Pairwise 3* *(MII_Endpoint –_ GV_Endpoint_)*	22141	13 (0.06%)	22128(99.94%)	2 (15.4%)	11 (84.6%)

The identification of DEGs in the GeneChip array analysis using Affymetrix was based on filtering criteria of a fold change greater than 2 and a p-value greater than 0.05.

### 3.2 Driver genes of CC derived from EAfs enclosing competent and incompetent oocytes

#### 3.2.1 Signaling modules of network 1(MII_Endpoint_- GV_Startpoint_) and network 2 (GV_Endpoint_-GV_Startpoint_)

The network datasets were built up by excluding non-characterized genes, long non-coding RNAs, and genes without protein annotations. Specifically, 17.5% (242/1,386) of genes were removed from Pairwise 1, and 15% (214/1,420) were excluded from Pairwise 2 (**Supplementary Datasheet 4**). The refined datasets (**Supplementary Table 2**) were used to generate two different Networks based on DEGs named: Network 1: MII_Endpoint_ *vs* GV_Startpoint;_ Network 2: GV_Endpoint_ *vs* GV_Startpoint._ Network 1 (MII_Endpoint_ – GV_Startpoint_) contained 1,144 nodes, 7,388 edges, and 84 connected components, while Network 2 (GV_Endpoint_ - GV_Startpoint_) had 1,206 nodes, 7,617 edges, and 60 connected components. Topological analysis confirmed that both networks were scale-free (**Supplementary Datasheet 1**).

**Figure 2:**
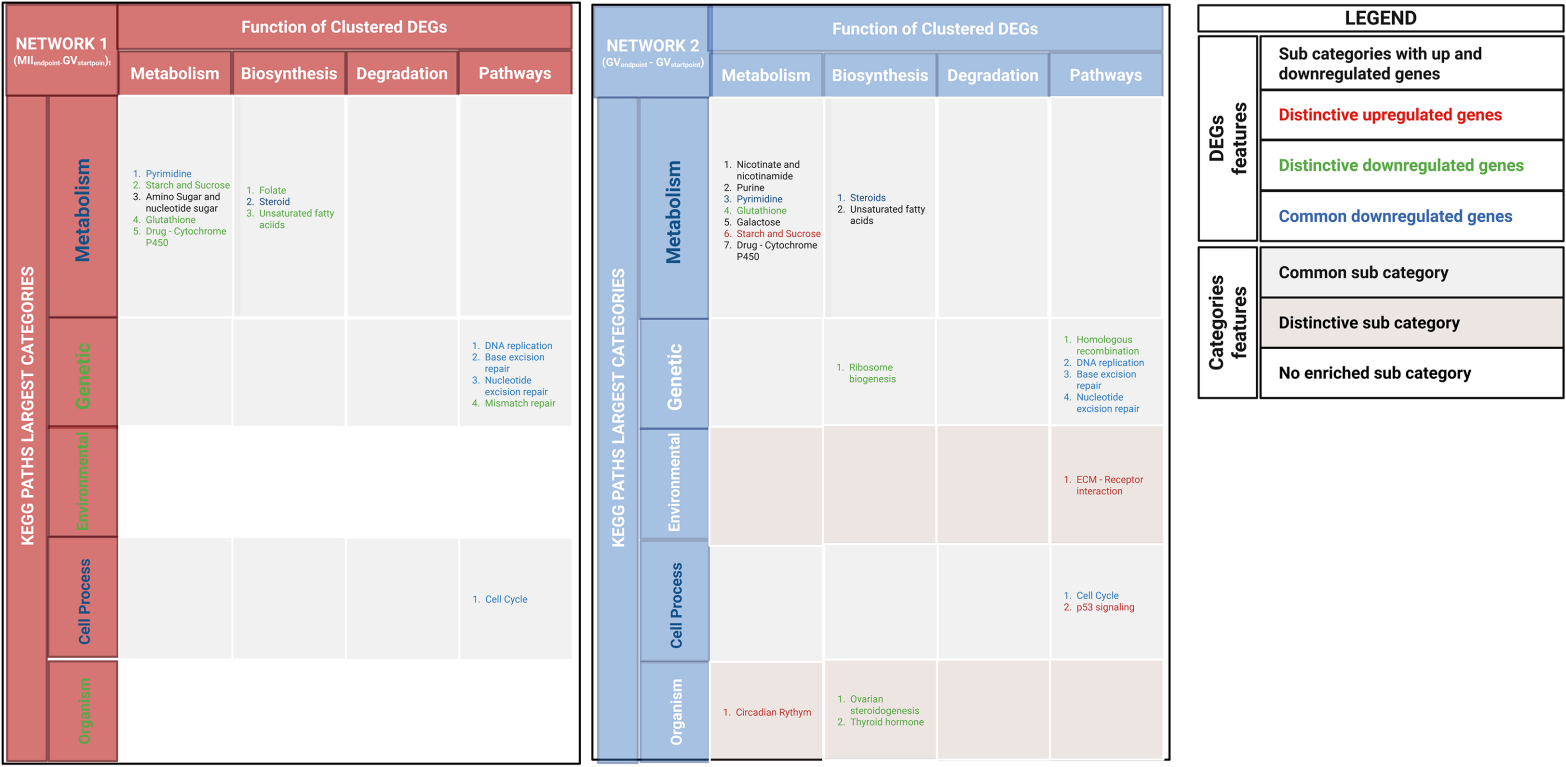
DEG modules in network 1(MII_Endpoint_- GV_Startpoint_) and network 2 (GV_Endpoint-_GV_Startpoint_) were identified using the MCODE plugin in Cytoscape and annotated with ClueGO. KEGG pathways were categorized into major groups, with a legend detailing DEG features and transcriptional regulation levels. Colors indicated shared and unique DEGs and KEGG categories.

The MCODE clustering algorithm was applied to identify highly connected DEG modules, which were then grouped into the seven largest KEGG pathway categories using the ClueGO plug-in (**Supplementary Table 3**; **Figure 2**).

Network 1 (MII_Endpoint_ *–* GV_Startpoint_) identified 14.79% (170/1,144) of highly connected DEGs, which were clustered into 8 modules. Network 2 (GV_Endpoint_ *-*GV_Startpoint_) identified 21.4% (259/1,206) of highly connected DEGs, which were clustered into 9 modules. The identified modules in Network 1(MII_Endpoint_- GV_Startpoint_) and Network 2(GV_Endpoint_ *-*GV_Startpoint_) (**Supplementary Table 3**), respectively, belonged to: Three KEGG pathway categories – metabolism, genetic processes, and cellular processes. Five KEGG pathway categories – metabolism, genetic processes, cellular processes, organismal systems, and environmental interactions. Notably, environmental and organism are the distinctive KEGG categories of Network 2 (GV_Endpoint_- GV_Startpoint_).

In more detail, environmental category primarily includes upregulated DEGs, particularly those involved in ECM-receptor interactions. The organismal systems category contains both up- and downregulated DEGs, spanning pathways such as circadian rhythm, ovarian steroidogenesis, and thyroid hormone signaling. Notably, within this category, downregulated DEGs in the ovarian steroidogenesis pathway are uniquely associated with oocyte meiotic failure. These DEGs include genes essential for hormonal signaling (*FSHR, CYP19*) and intracellular signaling (*PRKACA*) controlling cumulus expansion and oogenesis. Instead, metabolism, genetic processes, and cellular processes categories were shared between both networks.

However, **Figure 2** showed that these categories recognize also distinguishing features as specific network pathways or distinctive regulatory signaling.

#### 3.2.2 Driver DEGs of network 1 (MII_Endpoint_-GV_Startpoint)_ and network 2 (GV_Endpoint_-GV_Startpoint_)

To identify CC driver genes, clustered DEGs from MCODE were filtered for *(i)* highly modulated or *(ii)* highly interconnected (HUB) genes. Modulation analysis identified *(i)* co-regulated genes with similar biological roles, *(ii)* while interconnection analysis identified HUBs as key regulatory genes. Lists of highly modulated genes and HUBs were compiled, followed by a literature review to assess their role in oocyte maturation.

##### 3.2.2.1 Highly modulated DEGs of network 1(MII_Endpoint_- GV_Startpoint_) and network 2(GV_Endpoint_-GV_Startpoint_)

**Figure 3:**
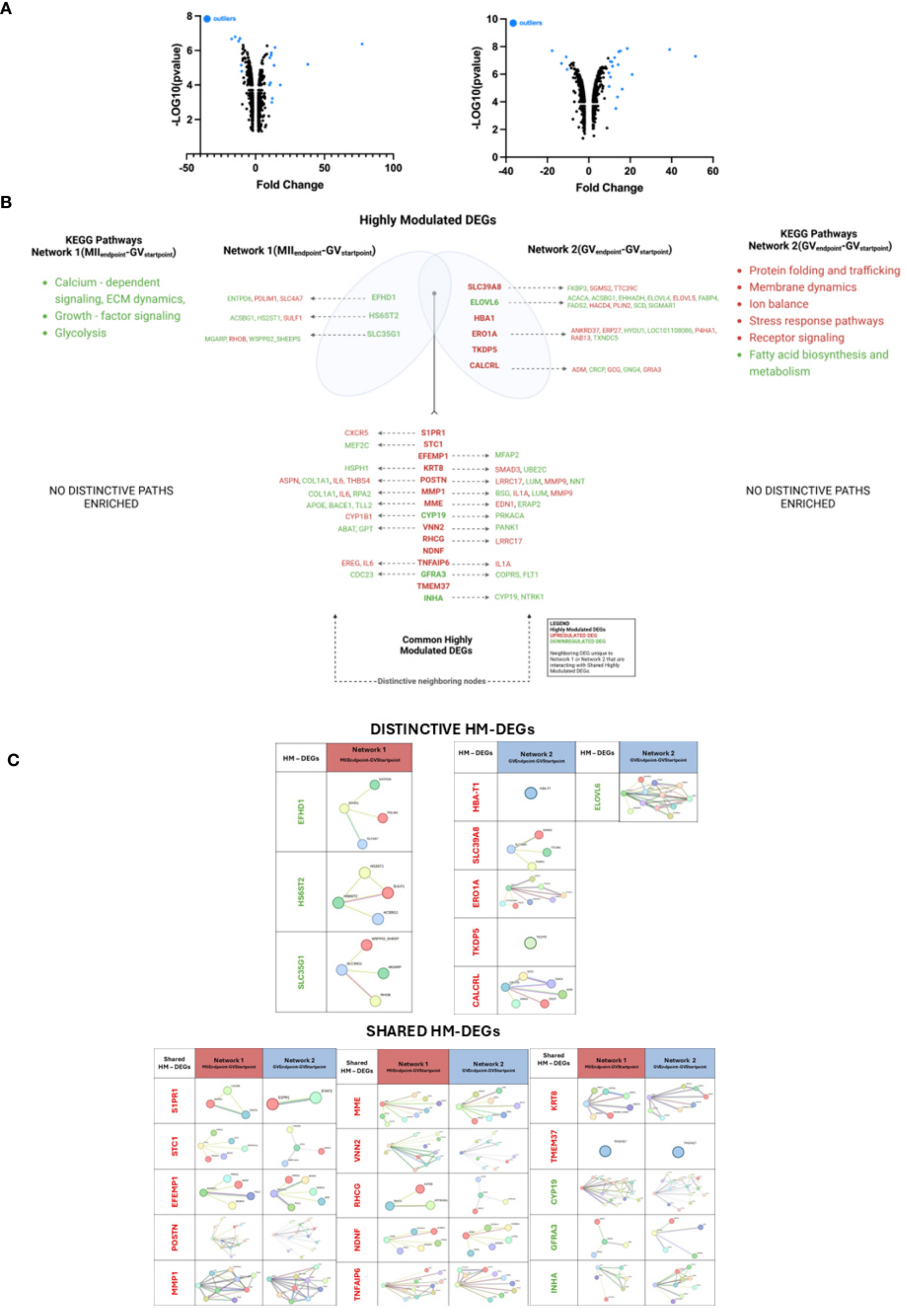
Key highly modulated DEGs (HM-DEGs) and their neighbors in EAf CCs with competent and incompetent oocytes. **(A)** Volcano plot illustrates upregulated (right part of each graph) and downregulated (left part of each graph) DEGs in Networks 1(MII_Endpoint-_GV_Startpoint_) - [left panel] and 2 (GV_Endpoint-_GV_Startpoint_) - [right panel] with highly modulated outliers circled. **(B)** Venn diagram showing shared and unique outlier DEGs between the networks, with highly interconnected module DEGs in bold. **(C)** Highly modulated differentially expressed genes (HM-DEGs) identified in Network 1(MII_Endpoint-_GV_Startpoint_) and in Network 2 (GV_Endpoint-_GV_Startpoint_). The subpanels show selected genes and their interaction networks in this group. Nodes represent genes, and edges denote interactions. Created with BioRender.com.

Highly modulated DEGs (outliers) were identified using gene stratification based on significant deviation of expression from average modulation levels and statistical significance (**Figure 3A**). As shown in, the highly modulated DEGs of Network 1 (MII_Endpoint_-GV_Startpoint_) and Network 2 (GV_Endpoint_-GV_Startpoint_) are 18 and 21, respectively. Moreover, the shared and distinctive ones are detailed in **Figures 3B, C**.

Specifically, the distinctive highly modulated DEGs include Network 1(MII_Endpoint_-GV_Startpoint)_: 3 downregulated genes (*EFHD1, HS6ST2, SLC35G1*); Network 2 (GV_Endpoint_-GV_Startpoint_): 5 upregulated *genes (HBA1, SLC39A8, ERO1A, TKDP5, CALCRL)* and 1 downregulated gene *(ELOVL6).*

To gain insight into the functional roles of the distinctive highly modulated genes, their relationships with neighboring nodes were analyzed (**Figure 3C**; **Supplementary Table 4**), along with their corresponding KEGG pathway categories (**Supplementary Datasheet 5**).

In Network 1(MII_Endpoint_- GV_Startpoint_), these genes co-operate in regulating ECM remodeling. More in detail, *EFHD1* interacted with 3 additional DEGs involved in calcium-dependent signaling, ECM stability and pH regulation (**Figures 3B, C**; **Supplementary Table 4**, **Datasheet 5**). *HS6ST2* and its 3 co-neighboring regulate growth factor signaling and ECM dynamics. While *SLC35G1* along with 3 additional DEGs, were involved in glycolysis, structural remodeling, and ECM production.

In Network 2 (GV_Endpoint_-GV_Startpoint_), the joint action of highly modulated genes with their neighboring nodes were classified under functional control of cellular homeostasis and metabolic regulation, specifically (**Figures 3B, C**; **Supplementary Table 4**, **Datasheet 5**) *ELOVL6* (downregulated) and its network of 11 neighboring genes. Together they contribute to metabolic regulation and lipid homeostasis by controlling energy balance and membrane remodeling. *SLC39A8* and *ERO1A* (both upregulated) participated in proteostasis (Protein Homeostasis). *SLC39A8* with three additional DEGs engaged in protein folding, trafficking and membrane dynamics, ensuring proper protein function and cellular integrity. *ERO1A* together with 7 DEGs overall control stress response, regulation of protein folding and redox balance. Meanwhile, *CALCRL*, along with five additional DEGs cell signaling and stress response tackling the receptor signaling, ion balance and stress response pathways, which are vital processes for cellular adaptation to environmental changes. Notably, *HBA1* and *TKDP5* had no neighboring partners.

A similar analysis was conducted also on the highly modulated genes shared between Network 1(MII_Endpoint_- GV_Startpoint_) and Network 2(GV_Endpoint_-GV_Startpoint_). However, no distinct relationships with different neighboring genes were identified (**Figure 3C**; **Supplementary Table 4**, **Datasheet 5**).

##### 3.2.2.2 HUBs of network 1(MII_Endpoint_-GV_Startpoint_) and network 2(GV_Endpoint_-GV_Startpoint_)

**Figure 4:**
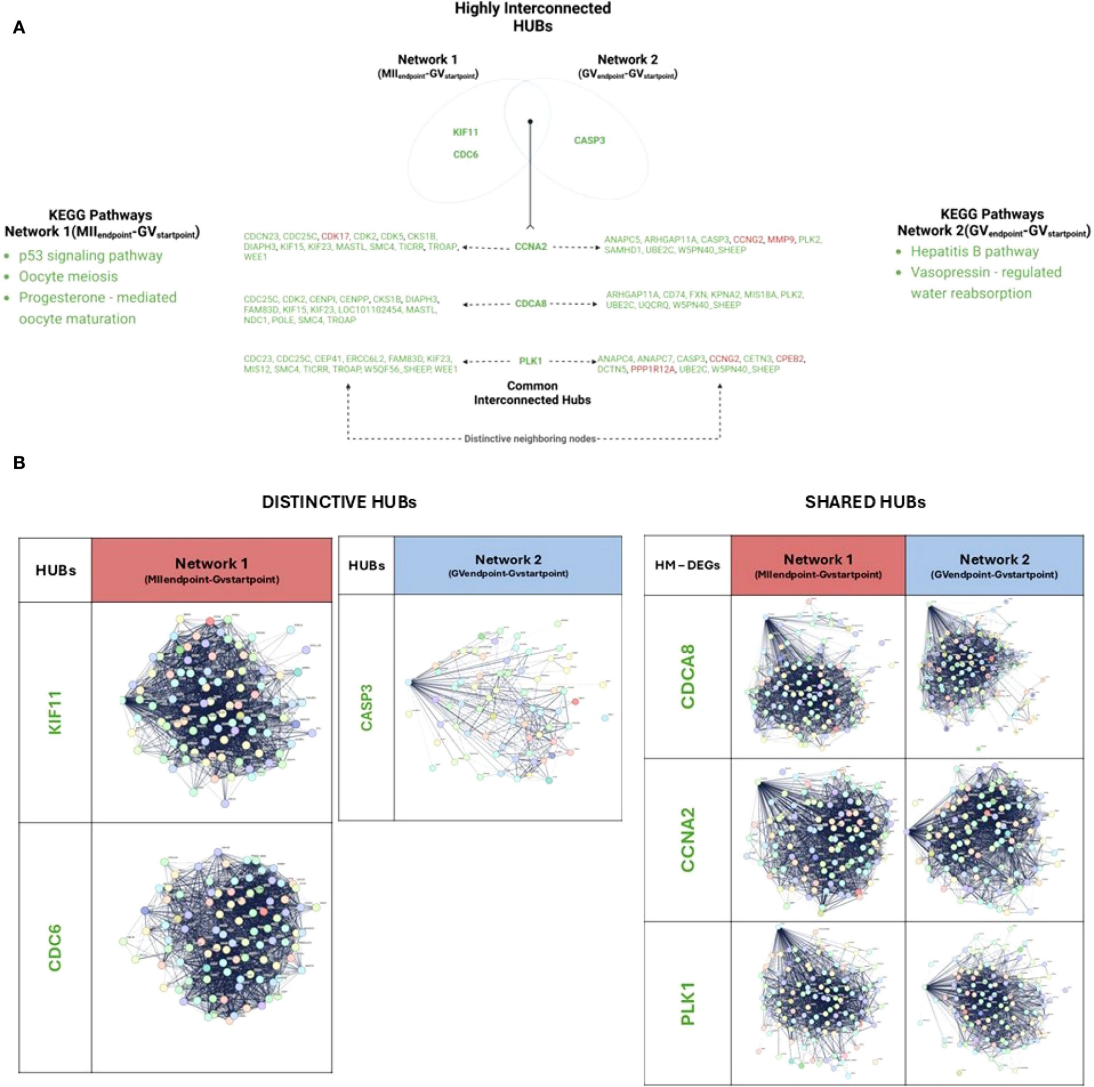
Network analysis of HUB DEGs with interacting partners in EAf CCs enclosing competent and incompetent oocytes. **(A)** Venn diagram showing shared and unique HUBs between the networks and their neighbors. **(B)** Distinctive and shared HUBs identified in both networks. The subpanels show selected genes and their interaction networks in this group. Nodes represent genes, and edges denote interactions. Created with BioRender.com.

HUBs selection began by ranking the top 10 DEGs for each of 12 centrality coefficients: closeness, degree, MCC, radiality, stress, MCN, DNMC, betweenness, clustering coefficient, eccentricity, bottleneck, and EPC. Genes that met at least five out of 12 centrality criteria were classified as HUBs. Detailed information on HUB selection can be found in **Supplementary Table 1**. As previously performed, both distinctive and shared HUBs between Network 1 (MII_Endpoint_-GV_Startpoint_) and Network 2 (GV_Endpoint_-GV_Startpoint_) were identified. The relationships of HUBs with neighboring controlled nodes were analyzed (**Figure 4**; **Supplementary Table 5**, **Datasheet 6**) before defining their affiliation to KEGG path largest categories (**Supplementary Table 6**).

Three distinctive HUBs were identified across the networks, in Network 1(MII_Endpoint-_GV_Startpoint_): *KIF11* and *CDC6*, as downregulated HUBs. In parallel, in Network 2 (GV_Endpoint-_GV_Startpoint_) *CASP3*, as a downregulated HUB (**Figure 4A**).Functionally, *CDC6* and *KIF11* along their 99 and 98 downstream DEGs, respectively (**Figure 4B**; **Supplementary Table 5**, **Datasheet 6**), influence key KEGG pathways, including cell cycle, DNA replication, homologous recombination, oocyte meiosis, *p53* signaling, and progesterone-mediated oocyte maturation.

*CASP3*, distinctive HUB of Network 2, interacts with 72 downstream DEGs (**Figure 4B**), clustering into pathways related to apoptosis and thyroid hormone synthesis, as well as cell cycle, DNA replication, oocyte meiosis, and *p53* signaling (**Figure 4A**; **Supplementary Table 5**, **Datasheet 6**).

Three shared HUBs, downregulated DEGs in both networks, were identified: *CDCA8*, *CCNA2*, *PLK1 (* **Figure 4**). They influence downstream DEGs predominantly grouped into similar KEGG pathways. However, a subset of them diverges between the two networks, leading to distinct KEGG pathway enrichments and to specific network functions.

This analysis documented (**Figure 4**; **Supplementary Table 6**, **Datasheet 7**) that *CDCA8* controls *108* downstream DEGs in Network 1(MII_Endpoint_- GV_Startpoint_) and 100 in Network 2(GV_Endpoint_-GV_Startpoint_). The strictly network-dependent DEGs were 15 and 9, respectively. Amongst them, *CDC25C* and *CDK2* are notable Network 1(MII_Endpoint_- GV_Startpoint_) – dependent regulators of oocyte maturation (*p53* signaling, oocyte meiosis and progesterone-mediated oocyte maturation).

Also, *CCNA2* controls 110 and 105 downstream DEGs in Network 1(MII_Endpoint_- GV_Startpoint_) and Network 2(GV_Endpoint_-GV_Startpoint_), respectively. The strictly network-dependent genes were 14 and 9, respectively. Amongst them, *KIF23* and *CDC25C* in Network 1(MII_Endpoint_- GV_Startpoint_) were recognized as notable MicroRNAs regulators of cancer pathway while *MMP9* and *CASP3* are functional related in Network 2(GV_Endpoint_-GV_Startpoint_) to Hepatitis B pathway.

Additionally reported, *PLK1* interacts with 114 genes in Network 1(MII_Endpoint_- GV_Startpoint_) 107 in Network 2(GV_Endpoint_-GV_Startpoint_). The downstream distinctive genes are 12 and 10 in Network 1(MII_Endpoint_- GV_Startpoint_) and Network 2(GV_Endpoint_-GV_Startpoint_), respectively. A Network 1(MII_Endpoint_- GV_Startpoint_) – specific function was recognized for *DTCN5*, a microtubule organizer involved in the Vasopressin-regulated water absorption KEGG pathway.

**Figure 5:**
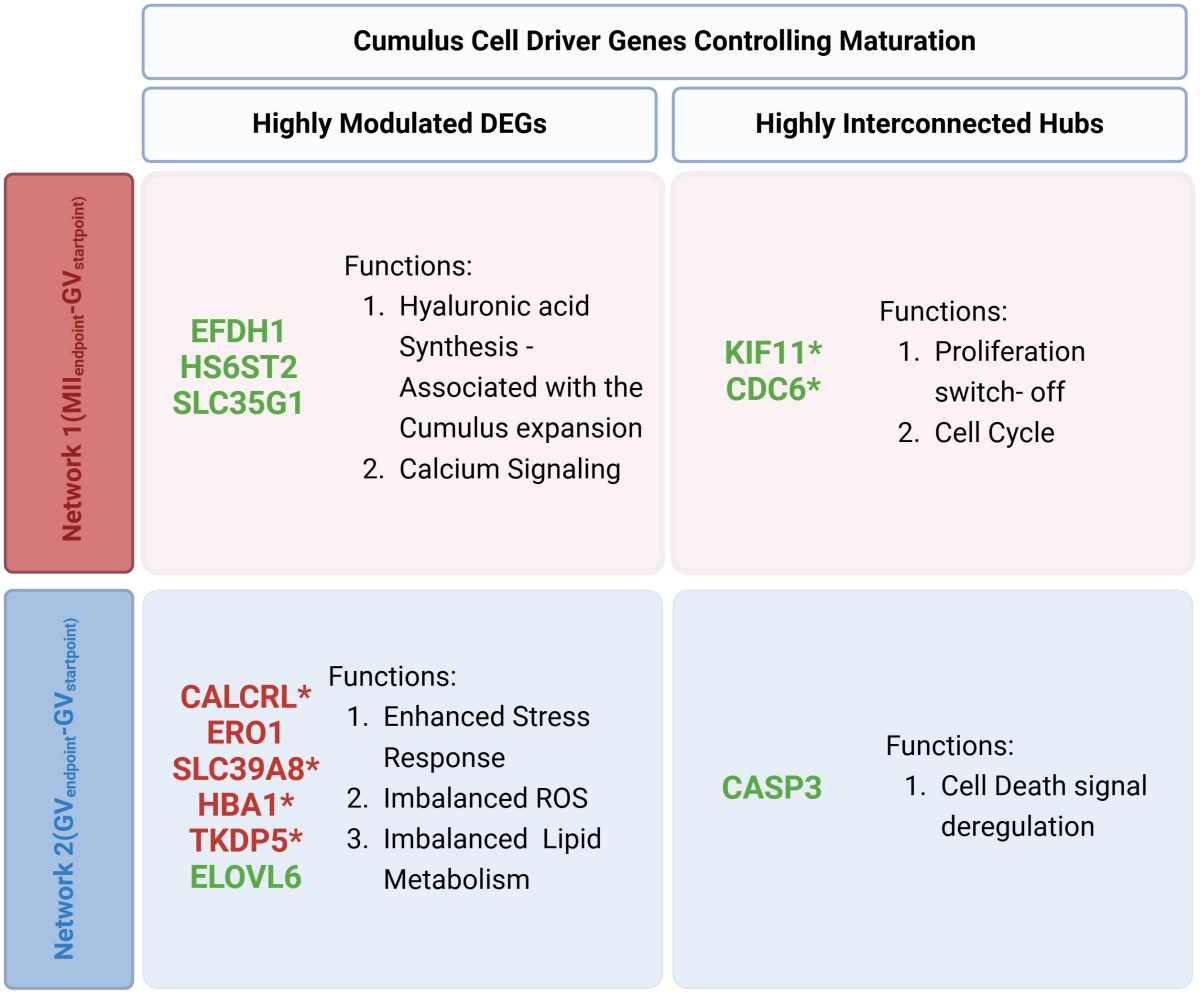
Summary of distinct biological processes governing each network’s functional network. The figure illustrates the comparison of Driver DEGs between Network 1(MII_Endpoint_- GV_Startpoint_) and Network 2(GV_Endpoint_-GV_Startpoint_) highlighting highly modulated DEGs and highly interconnected hubs and the key action in each outcome. (*) 6 novel genes with potential regulatory elements in the maturation process, never previously shown to have any role in follicular development and oocyte maturation.

The functional interaction of highly modulated DEGs and HUBs provides new CC biological insights by identifying network-specific maturation drivers (**Figure 5**). Network 1(MII_Endpoint_- GV_Startpoint_) indicates that CCs, that release competent and successfully matured oocytes, rely on three downregulated highly modulated outliers (*EFHD1*, *HS6ST2*, *SLC35G1*) and two downregulated HUBs (*KIF11*, *CDC6*) which use their neighboring interactors overall to regulate in CCs hyaluronic acid synthesis (cumulus expansion), proliferation switch-off, and calcium signaling.

Conversely, Network 2(GV_Endpoint_-GV_Startpoint_) revealed seven highly modulated outliers (5 upregulated: *HBA1*, *SLC39A8*, *TKDP5*, *CALCRL*, *ERO1* and, 2 downregulated: *SLC39A8, ELOVL6)* and 1 downregulated HUB *(CASP3).* These genes were linked to stress responses, imbalanced ROS levels, lipid metabolism disruptions, and abnormal cell death regulation.

The computational analysis identified 12 distinctive driver genes, of which only 7 have been previously documented to play a role in meiotic maturation (**Supplementary Datasheet 8**). The remaining 5 genes may represent novel regulatory elements in the maturation process, with their involvement in key biological pathways highlighting their potential significance in oocyte development and competence acquisition.

### 3.3 Key genes defining CCs enclosing competent and incompetent oocytes at maturation endpoint

**Figure 6:**
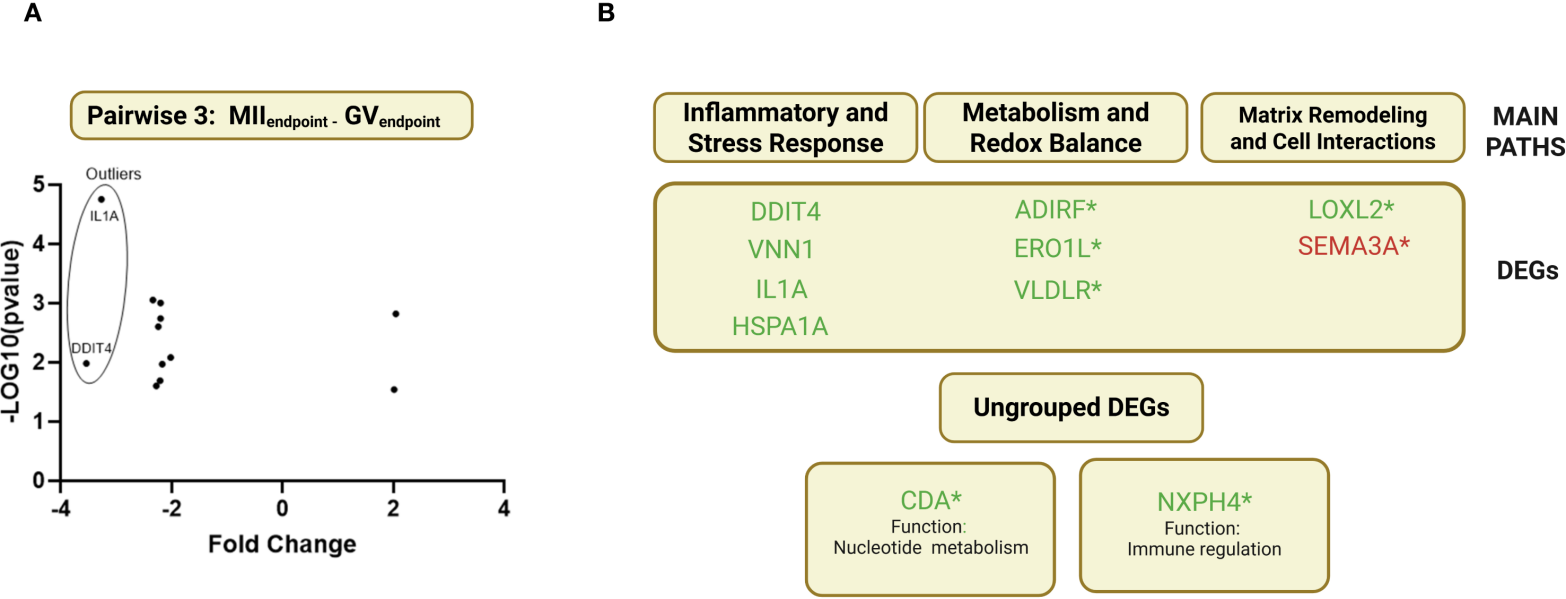
Pairwise 3 (MII_endpoint-_GV_endpoint_) clustering DEGs and classification for biological processes. **(A)** Volcano plot showing the upregulated and downregulated DEGs in Pairwise 3(MII_Endpoint-_GV_Endpoint_). **(B)** DEGs retrieved seeking gene-by-gene in each cellular pathway. (*) 7 novel genes with potential regulatory elements in the maturation process, never previously shown to have any role in follicular development and oocyte maturation.

The study also aimed to identify CC gene markers for successful oocyte maturation by comparing the transcriptomes of CCs surrounding MII and GV oocytes (*see* AIM 2 **Figure 1**). After excluding two uncharacterized transcripts, 11 DEGs were identified (**Figure 6**): 1 upregulated (*SEMA3A*) and ten downregulated (*IL1A, NXPH4, HSPA1A, LOXL2, VNN1, DDIT4, CDA, ADIRF, ERO1L*). *IL1A* and *DDIT4* were notably downregulated (**Figure 6A**).

Due to the limited number of DEGs in pairwise 3, MCODE clustering and computational driver filtering could not be applied. Instead, an individual gene analysis was performed, leveraging literature data to classify DEGs into three main biological pathways:

1. Inflammatory and Stress Response – *VNN1, IL1A, HSPA1A, DDIT4*.
2. Matrix Remodeling and Cell Interactions – *LOXL2, SEMA3A*.
3. Metabolism and Redox Balance – *VDLR, ADIRF, ERO1L* (**Figure 6B**).

Two DEGs, *CDA* and *NXPH4*, could not be assigned to any specific pathway. *CDA* (Cytidine Deaminase) is involved in protein coding, while *NXPH4* (Neurexophilin 4) has an undefined role but is predicted to facilitate signaling receptor binding.

Additionally, a literature review was conducted to assess the potential involvement of the identified DEGs in the meiotic maturation process (**Supplementary Datasheet 8**).

The literature revision showed that out of the 11 endpoint DEGs 4 were previously recognized through scientific evidence confirming their role on meiotic maturation *(IL1A, HSPA1, VNN1, DDIT4*). While the remaining 7 genes (*ADIRF1, SEMA3A, NXPH4, ERO1L, LOXL2, VLDLR, CDA*) have not been previously described in this specific reproductive model thus suggesting them as new players in CC mediating oocyte maturation process.

### 3.4 Molecular validation and spatial mapping of cumulus cell markers of oocyte competence

Quantitative RT−PCR confirmed the differential expression of the principal drivers identified in each pairwise comparison of the ovine transcriptome (**Supplementary Datasheet 9**). Specifically, *HS6ST2* and *CDC6* from Network 1 (MII_Endpoint_ vs GV_Startpoint)_, *ERO1A* and *CASP3* from Network 2 (GV _Endpoint_ vs GV_Startpoint_), and *SEMA3A* from Network 3 (MII_Endpoint_ vs GV_Endpoint_) all showed changes in the same direction and of comparable magnitude to those detected on the microarray.

**Figure 7:**
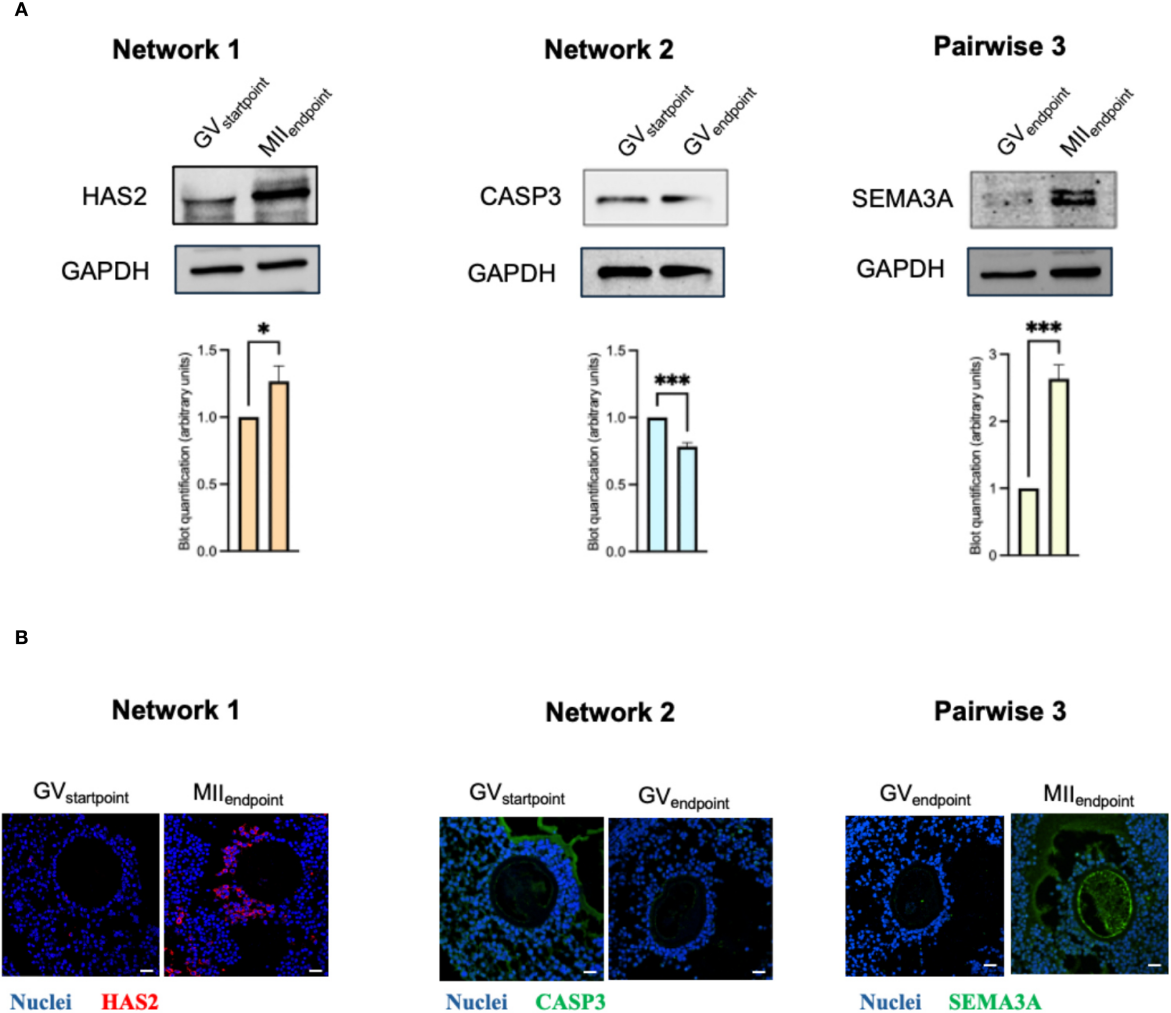
Spatial distribution and expression of cumulus cell genes linked to oocyte maturity. Validation of drivers through immunoblotting and immunofluorescence **(A, B)** analyses. *HAS2* was shown for Network 1(MII_Endpoint_- GV_Startpoint_); *CASP3* for Network 2(GV_Endpoint_-GV_Startpoint_); *SEMA3A* for Pairwise 3 (MII_Endpoint_- GV_Endpoint_. **(A)** Immunoblotting of the selected drivers. Blot quantification was made with ImageLab software by Bio-Rad. Data (mean ± SD) represent 3 independent sets of experiments (n = at least 3 biological replicates in each group per set; each biological replicate assayed in at least 3 technical replicates). * and *** Statistically significant values between the different studied groups (p<0.05 and p<0.001respectively). **(B)** Representative confocal images of co-immunofluorescence staining of nuclei (Hoechst), Alexa-Fluor 488 (*SEMA3A* or *CASP3*) or Alexa-Fluor 568 (*HAS2*) in CCs enclosing oocytes in the different nuclear stage. *HAS2* and *SEMA3A* reactions for the MII_Endpoint_ group were carried out on serial sections immediately adjacent to the equatorial plane of the same EAf. The equatorial section itself was used to document the oocyte nuclear stage (see **Supplementary Datasheet 10**). Likewise, *CASP3* and *SEMA3A* reactions for the GV_Endpoint_ group were performed on sections contiguous to the equatorial plane of the same EAf. Scale bar: 25 µm.

To determine whether these transcriptional cues translated into protein−level differences and to define their spatial distribution within the cumulus–oocyte complex (COC), we carried out immunodetection for one representative driver per comparison: *HAS2, CASP3*, and *SEMA3A*, respectively (**Figure 7**). In every case the protein data mirrored the qRT−PCR results (**Supplementary Datasheet 9**; **Figure 7A**). Moreover, the localization patterns provided functional context: *HAS2* and *CASP3* signals were confined to the cumulus compartment, whereas *SEMA3A* was detected both in cumulus cells and at the oolemma, with a pronounced accumulation in the surrounding follicular fluid of oocytes that had progressed to metaphase II (**Figure 7B**; **Supplementary Datasheet 10**). This compartment−specific distribution supports a cooperative role of somatic and germ−line cells in the acquisition of meiotic competence within our *in vitro* follicle culture system.

## 4 Discussion

This study, conducted using a macroarray, provides compelling evidence that oocyte maturation competence is orchestrated through network-specific transcriptional programs in CCs, identifying novel driver and hub genes that underpin this process. The use of a macroarray for the transcriptomic characterization presented here was motivated by the fact that the experimental model is based on the ovine species (*Ovis aries*), whose genome assembly still lacks high coverage in several regions. This incomplete annotation reduces the reliability of transcript quantification by RNA-seq. In contrast, the Ovine MicroArray GeneChip System provides probe sets covering the entire *Ovis aries* transcriptome, enabling a reliable, automated, and statistically validated analysis of global gene expression. By leveraging a computational approach to analyze DEGs, we delineated two distinct regulatory networks associated with divergent maturation outcomes in EAfs, a gamete source of increasing relevance in ART due to their high yield, shorter *in vitro* maturation timelines, and epigenetic flexibility (3, 9–11). In addition, we extended this framework to CC, thereby completing the picture of hCG-dependent somatic signaling pathways. Indeed, in a previous study we conducted a transcriptomic characterization with oocyte fate distinction in follicular wall cells (FC) of EAfs subjected to IVM, which further supports this integrative view (29).

In the effort to address the biological question defined in Aim 1, the study systematically delineated multiple gene interaction networks underlying oocyte maturation competence. Network 1 (MII_Endpoint_–GV_Startpoint_), linked to successful maturation, features the downregulation of key outliers (*EFHD1, HS6ST2, SLC35G1*) and hub genes (*KIF11, CDC6*), which collectively influence calcium homeostasis, cell cycle control, and hyaluronic acid synthesis, essential for cumulus expansion and follicular responsiveness. Notably, our findings on *HS6ST2* suppression align with prior data implicating excessive proteoglycan sulfation as inhibitory to cumulus expansion *in vitro*, correlating inversely with markers like *HAS2* and *TNFAIP5 (* 37). Elevated *HAS2* expression and reduced *HS6ST2* at both mRNA and protein levels in competent CCs support this regulatory balance. Further, *EFHD1* and *SLC35G1*, known mediators of intracellular calcium dynamics crucial for follicular luteinization, were also suppressed in the competent group (38). In contrast, Network 2 (GV_Endpoint_–GV_Startpoint_), reflecting meiotic arrest, was characterized by dysregulated lipid metabolism and apoptosis signaling, as seen in the upregulation of *ERO1* and downregulation of *ELOVL6* and *CASP3*. These alterations echo previous reports emphasizing the role of lipid precursors in oocyte viability (38, 39) and suggest that elevated *ERO1* may signal metabolic stress or impaired apoptosis (40). The reduced *CASP3*, a known *LH* downstream effector, further substantiates defective apoptotic priming in non-competent follicles (41, 42).

It can be hypothesized that the regulatory complexity observed in CCs may involve a broader set of molecular players than previously recognized, as suggested by our identification of several promising targets not yet known for their roles in oocyte maturation. Accordingly, among the twelve driver genes highlighted in this study, only half have established roles in meiotic progression, while the remaining six—including two hub genes in Network 1 (*KIF11* and *CDC6*), potentially involved in modulating proliferative slowdown to support cumulus expansion, four highly modulated genes (*CALCRL, SLC39A8, HBA1, TKDP5*), and the hub gene *CASP3* in Network 2, associated with dysregulated ROS, lipid metabolism, and apoptotic signaling—emerge as novel candidates potentially influencing oocyte fate during FEO-IVM.

In Aim 2, we identified DEGs in CCs at key final stages of oocyte maturation. Eleven DEGs were significantly modulated, with *SEMA3A* being the only gene upregulated in competent CCs, while others such as *IL1A a*nd *DDIT4* were markedly downregulated, suggesting differential regulation of immune and stress response pathways. *IL1A*, a pro-inflammatory cytokine previously implicated in folliculogenesis (43, 44), showed decreased expression in follicles supporting maturation, possibly indicating that a reduced inflammatory environment favors oocyte competence. Similarly, downregulation of *DDIT4*, an mTOR pathway inhibitor involved in cellular stress responses (45), points to a minimized stress signaling environment conducive to maturation. *VNN1* and *HSPA1A* were also implicated in overlapping oxidative stress and immune signaling pathways, emphasizing the role of redox regulation in late folliculogenesis. Mechanisms related to extracellular matrix remodeling and cell communication also emerged as central, with *LOXL2* (46, 47) and *SEMA3A* contributing to CC–oocyte interactions. Notably, *SEMA3A* upregulation was confirmed at transcript and protein levels, and its established involvement in reproductive disorders like Kallmann Syndrome and hypogonadotropic hypogonadism (48, 49), along with its regulatory role in angiogenesis in gonadotropin-stimulated ovarian cancer (50), underscores its relevance. High *SEMA3A* levels have been observed in women with diminished ovarian reserve who respond well to ovarian stimulation (51), while other *SEMA* family members are known mediators of folliculogenesis and cumulus–oocyte communication (52–54). Additionally, the transcriptomic landscape suggested metabolic and oxidative stress modulation as crucial determinants, with genes like *ERO1L* (41), ADIRF, and *VLDLR* contributing to redox homeostasis and reproductive regulation (55). *ERO1L*’s downregulation in competent follicles indicates a finely regulated oxidative environment essential for high-quality oocyte development. Although *NXPH4* and *CDA* could not be integrated into known pathways, their modulation points to possible roles in nucleotide metabolism and signaling that warrant further investigation.

## 5 Conclusion

This study reveals distinct transcriptomic signatures in CCs associated with oocyte competence, highlighting key pathways in hyaluronic acid synthesis, lipid metabolism, inflammation, apoptosis, and extracellular matrix remodeling. The study also highlights several DEGs—some newly implicated in oocyte competence (*SEMA3A, ERO1L, LOXL2, ADIRF, CDA, NXPH4, VLDLR*)—as critical molecular markers. The identification of these genes, particularly the highly modulated *IL1A, VNN1, DDIT4*, and *SEMA3A*, not only deepens understanding of CC function but also paves the way for innovative strategies in assisted reproduction and fertility preservation.

## Data Availability

The original contributions presented in the study are included in the article/**Supplementary Material**, further inquiries can be directed to the corresponding author/s.

## Supplementary material

Supplementary Table 1 (Excel). The 12 centrality coefficients of each DEG of Network 1(MII_Endpoint_- GV_Startpoint_) (Sheet: N1 MII-GV) and Network 2(GV_Endpoint_-GV_Startpoint_) (Sheet: N2 GV-GV) were scored using CytoHUBba. More in detail, they are closeness, degree, MCC, radiality, stress, MCN, DNMC, betweenness, clustering coefficient, eccentricity, bottleneck, and EPC. Network 1(MII_Endpoint_- GV_Startpoint_) and Network 2(GV_Endpoint_-GV_Startpoint_) top 10 DEGs defined on each centrality coefficient score (Sheets: Top 10 N1 and N2 respectively). Venn diagram analysis of the top 10 DEGs of Network 1(MII_Endpoint_- GV_Startpoint_) (Sheet: Ranking N1) and Network 2(GV_Endpoint_-GV_Startpoint_)(Sheet: Ranking N2) shows DEGs overlapping across the 12 algorithms. DEGs that are in the top 10 in at least 5 of the 6 algorithms are highlighted in bold. (Network1_Normalized) and (Network2_Normalized) include dataset values that have been statistically normalized using the standard score formula.

Supplementary Table 2 (Excel). Network 1(MII_Endpoint_- GV_Startpoint_), Network 2(GV_Endpoint_-GV_Startpoint_) and Network 3(GV_Endpoint_-MII_Endpoint_) dataset with complete list of genes that passed filter and list of annotations that were filtered. Greater than 2-fold change and p value less than 0.05 (Sheet: Genes passed filter); Cleaned dataset without genes without an annotation and uncharacterized proteins (Sheet: Final Dataset). Dataset of proteins without an annotation (Sheet: Proteins no annotation). Dataset of all excluded annotations (Sheet: Eliminated annotations).

Supplementary Table 3 (Excel). MCODE clusters and ClueGo KEGG analysis. Resulting modules for Network 1(MII_Endpoint_- GV_Startpoint_) (Sheet: MCODE N1) and corresponding KEGGs (Sheet: ClueGo N1). Resulting modules for Network 2(GV_Endpoint_-GV_Startpoint_) (Sheet: MCODE N2) and corresponding KEGGs (Sheet: ClueGo N2).

Supplementary Table 4 (Excel). List of partners interacting with Highly – modulated DEGs of Network 1(MII_Endpoint_- GV_Startpoint_) and2(GV_Endpoint_-GV_Startpoint_). Results of partnering interactions with outliers (Sheet: Partners Outliers N1) (Sheet: Partners Outliers N2).

Supplementary Table 5 (Excel). List of partners interacting with distinctive HUBs of Network 1(MII_Endpoint_- GV_Startpoint_) and Network 2(GV_Endpoint_-GV_Startpoint_). Results of connecting neighbors with distinctive HUBs (Sheet: Partners HUBs N1) (Sheet: Partners HUBs N2).

Supplementary Table 6 (Excel). List of partners interacting with shared HUBs of network 1(MII_Endpoint_- GV_Startpoint_) and Network 2(GV_Endpoint_-GV_Startpoint_). Results of connecting neighbors with distinctive HUBs (Sheet: Shared HUBs N1) (Sheet: Shared HUBs N2).

## Glossary

ADIRF: Adipogenesis Regulatory Factor
ART: Assisted Reproductive Technologies
CALCRL: Calcitonin Receptor – like Receptor
CASP3: Caspase 3
CC: Cumulus cell
CCNA2: Cyclin A2
CDA: Cytidine Deaminase
CDC25C: Cell Division Cycle 25C
CDC6: Cell Division Cycle 6
CDCA8: Cell Division Cycle Associated 8
CDK2: Cyclin Dependent Kinase 1
COC: Cumulus-oocyte complex
CYP19: Cytochrome p450 Family 19
DEG: Differentially Expressed Gene
DDIT4: DNA Damage Inducible Transcript 4
DTCN5: Dynactin Subunit 5
EAfs: Early Antral Follicles
ECM: Extracellular Matrix
EFHD1: EF – Hand Domain Family Member D1
ELOVL6: ELOVL Fatty Acid Elongase 6
ERO1A: Endoplasmic Reticulum Oxidoreductase 1 Alpha
FEO: Follicle-Enclosed Oocyte
FEO - IVM: *In Vitro* Maturation of Follicle – Enclosed Oocytes
FSH: Follicle Stimulating Hormone
FSHR: Follicle Stimulating Hormone Receptor
GV: Germinal Vesicle
HBA1: Hemoglobin Subunit Alpha 1
hCG: human chorionic gonadotropin
HM - DEGs: Highly modulated – Differentially Expressed Genes
HSPA1A: Heat Shock Protein Family A Member 1A
HS6ST2: Heparan Sulfate 6 – O – Sulfotransferase 2
HUB: Highly interconnecting Genes
IL1A: Interleukin 1 Alpha
IVM: *In Vitro* Maturation
KIF11: Kinesin Family Member 11
KIF23: Kinesin Family Member 23
LH: Luteinizing Hormone
LOXL2: Lysyl Oxidase Like 2
MII: Metaphase II
MMP9: Matrix Metallopeptidase 9
NXPH4: Neurexophilin 4
OSE: Ovarian Surface Epithelial cells
PCL: Poly (Ɛ-caprolactone)
PRKACA: Protein Kinase CAMP – Activated Catalytic Subunit Alpha
ROS: Reactive Oxygen Species
SEMA3A: Semaphorin 3A
SLC35G1: Solute Carrier Family 35 Member G1
SLC39A8: Solute Carrier Family 39 Member A8
TKDP5: Trophoblast Kunitz Domain Protein 5
VNN1: Vanin 1
