# Supplementary material for: Tailor-made 3D in vitro maturation of early antral follicles uncovers cumulus-cell transcriptomic driver signature to predict oocyte competence
Source: Front Endocrinol (Lausanne). 2025 Oct 1;16:1629815. doi: 10.3389/fendo.2025.1629815 (PMC12520894; doi:10.3389/fendo.2025.1629815)
Supplement: Supplementary Table 1 — (Excel). The 12 centrality coefficients of each DEG of Network 1(MIIEndpoint- GVStartpoint) (Sheet: N1 MII-GV) and Network 2(GVEndpoint-GVStartpoint) (Sheet: N2 GV-GV) were scored using CytoHUBba. More in detail, they are closeness, degree, MCC, radiality, stress, MCN, DNMC, betweenness, clustering coefficient, eccentricity, bottleneck, and EPC. Network 1(MIIEndpoint- GVStartpoint) and Network 2(GVEndpoint-GVStartpoint) top 10 DEGs defined on each centrality coefficient score (Sheets: Top 10 N1 and N2 respectively). Venn diagram analysis of the top 10 DEGs of Network 1(MIIEndpoint- GVStartpoint) (Sheet: Ranking N1) and Network 2(GVEndpoint-GVStartpoint)(Sheet: Ranking N2) shows DEGs overlapping across the 12 algorithms. DEGs that are in the top 10 in at least 5 of the 6 algorithms are highlighted in bold. (Network1_Normalized) and (Network2_Normalized) include dataset values that have been statistically normalized using the standard score formula. [file DataSheet1.zip › Supplementary datasheets and tables/Supplementary Datasheet 7.docx]

**Datasheet 7.** **Network 1(MII_Endpoint_- GV_Startpoint_) and Network 2(GV_Endpoint_-GV_Startpoint_) partner analysis and KEGG pathway analysis of shared HUBs.**

SHARED HUBS BETWEEN N1 AND N2

| **CDCA8 (N1)** | | |
| --- | --- | --- |
| **Term** | **Nr. Genes** | **Associated Genes Found** |
| Pyrimidine metabolism | 3 | [RRM1, RRM2, TYMS] |
| DNA replication | 6 | [MCM3, MCM4, MCM5, MCM7, POLE, PRIM1] |
| Fanconi anemia pathway | 3 | [RAD51, UBE2T, USP1] |
| Cell cycle | 16 | [BUB1, BUB1B, CCNA2, CDC20, CDC25C, CDC6, CDK2, CDK4, CHEK1, MCM3, MCM4, MCM5, MCM7, ORC1, PLK1, TTK] |
| p53 signaling pathway | 4 | [CDK2, CDK4, CHEK1, RRM2] |
| oocyte meiosis | 6 | [BUB1, CDC20, CDC25C, CDK2, FBXO5, PLK1] |
| progesterone- mediated oocyte maturation | 5 | [BUB1, CCNA2, CDC25C, CDK2, PLK1] |
| 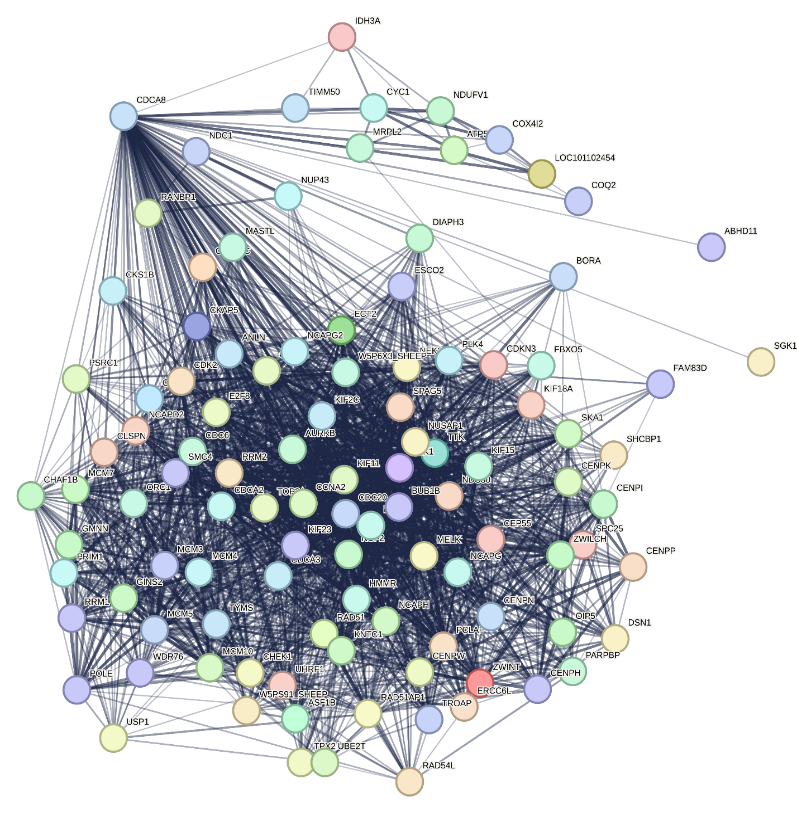 | | |

| **CDCA8 (N2)** | | |
| --- | --- | --- |
| **Term** | **Nr. Genes** | **Associated Genes Found** |
| Pyrimidine metabolism | 3 | [RRM1, RRM2, TYMS] |
| DNA replication | 5 | [MCM3, MCM4, MCM5, MCM7, PRIM1] |
| Fanconi anemia pathway | 3 | [RAD51, UBE2T, USP1] |
| Cell cycle | 14 | [BUB1, BUB1B, CCNA2, CDC20, CDC6, CDK4, CHEK1, MCM3, MCM4, MCM5, MCM7, ORC1, PLK1, TTK] |
| 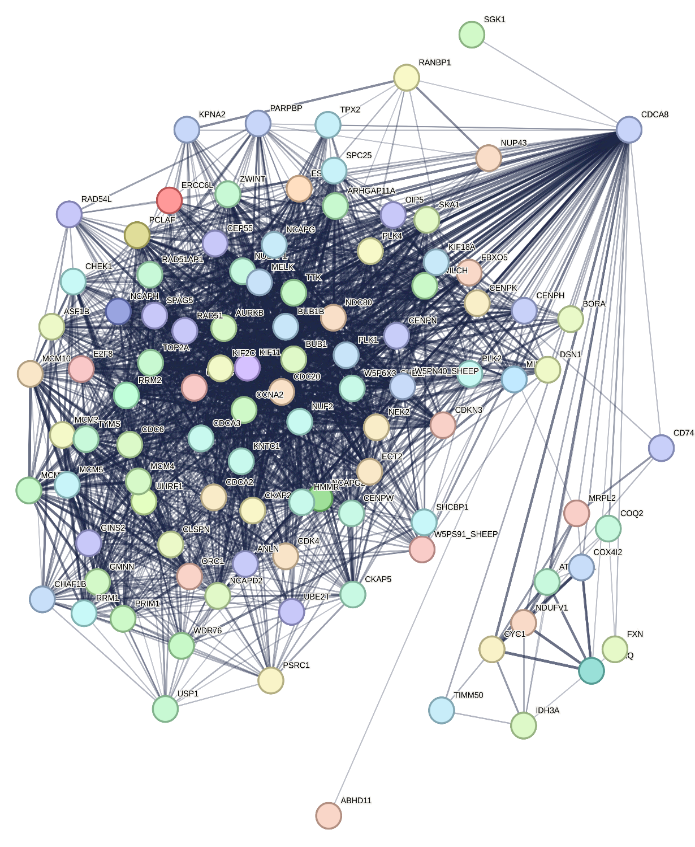 | | |

| **CCNA2 (N1)** | | |
| --- | --- | --- |
| **Term** | **Nr. Genes** | **Associated Genes Found** |
| Pyrimidine metabolism | 3 | [RRM1, RRM2, TYMS] |
| dna replication | 9 | [FEN1, MCM3, MCM4, MCM5, MCM6, MCM7, PCNA, PRIM1, RFC4] |
| MicroRNAs in cancer | 9 | [BRCA1, CCNE2, CDC25A, CDC25C, CDKN1A, E2F1, EZH2, KIF23, STAT3] |
| Cell cycle | 28 | [ANAPC5, BUB1, BUB1B, CCNA2, CCNE2, CDC20, CDC23, CDC25A, CDC25C, CDC6, CDC7, CDK2, CDK4, CDKN1A, CHEK1, E2F1, MCM3, MCM4, MCM5, MCM6, MCM7, ORC1, PCNA, PLK1, RBL1, SKP2, TTK, WEE1] |
| ubiquitin mediated proteolysis | 6 | [ANAPC5, BRCA1, CDC20, CDC23, SKP2, UBE2D1] |
| oocyte meiosis | 9 | [ANAPC5, BUB1, CCNE2, CDC20, CDC23, CDC25C, CDK2, FBXO5, PLK1] |
| progesterone-mediated oocyte maturation | 8 | [ANAPC5, BUB1, CCNA2, CDC23, CDC25A, CDC25C, CDK2, PLK1] |
| homologous recombination | 3 | [BARD1, BRCA1, RAD51] |
| fanconi anemia pathway | 4 | [BRCA1, FANCD2, RAD51, UBE2T] |
| FoxO signaling pathway | 6 | [CDK2, CDKN1A, PLK1, PLK4, SKP2, STAT3] |
| p53 signaling pathway | 6 | [CCNE2, CDK2, CDK4, CDKN1A, CHEK1, RRM2] |
| Cellular senescence | 9 | [CCNA2, CCNE2, CDC25A, CDK2, CDK4, CDKN1A, CHEK1, E2F1, RBL1] |
| Human T-cell leukemia virus 1 infection | 11 | [ANAPC5, BUB1B, CCNA2, CCNE2, CDC20, CDC23, CDK2, CDK4, CDKN1A, CHEK1, E2F1] |
| Viral carcinogenesis | 10 | [CCNA2, CCNE2, CDC20, CDK2, CDK4, CDKN1A, CHEK1, RBL1, SKP2, STAT3] |
| pancreatic cancer | 5 | [CDK4, CDKN1A, E2F1, RAD51, STAT3] |
| prostate cancer | 4 | [CCNE2, CDK2, CDKN1A, E2F1] |
| melanoma | 3 | [CDK4, CDKN1A, E2F1] |
| bladder cancer | 3 | [CDK4, CDKN1A, E2F1]] |
| small cell lung cancer | 7 | [CCNE2, CDK2, CDK4, CDKN1A, CKS1B, E2F1, SKP2] |
| non-small lung cancer | 4 | [CDK4, CDKN1A, E2F1, STAT3]] |
| 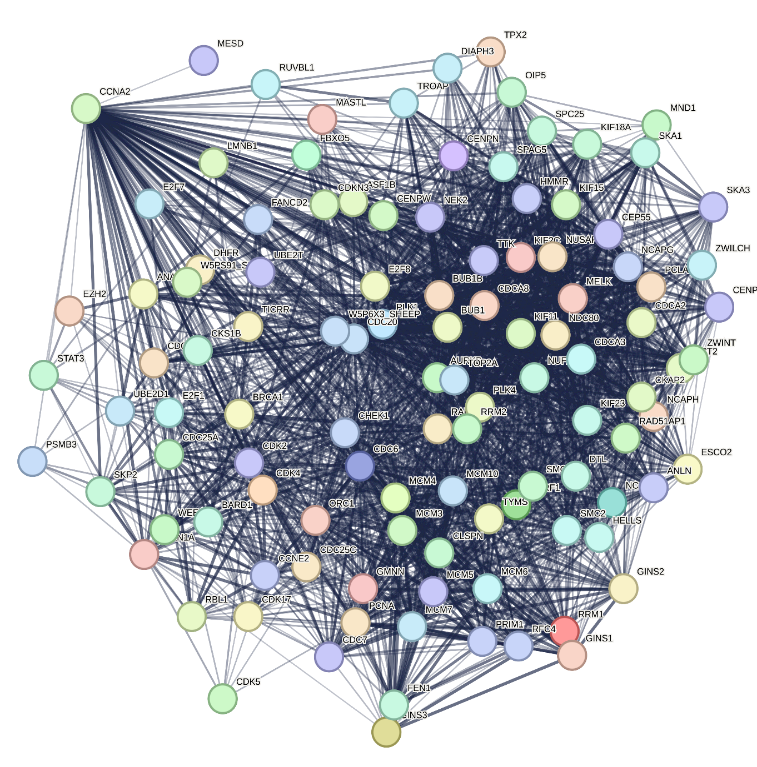 | | |

| **CCNA2 (N2)** | | |
| --- | --- | --- |
| **Term** | **Nr. Genes** | **Associated Genes Found** |
| Pyrimidine metabolism | 3 | [RRM1, RRM2, TYMS] |
| dna replication | 9 | [FEN1, MCM3, MCM4, MCM5, MCM6, MCM7, PCNA, PRIM1, RFC4] |
| FoxO signaling pathway | 7 | [CCNG2, CDKN1A, PLK1, PLK2, PLK4, SKP2, STAT3] |
| cell cycle | 26 | [ANAPC4, ANAPC5, ANAPC7, BUB1, BUB1B, CCNA2, CCNE2, CDC20, CDC25A, CDC6, CDC7, CDK4, CDKN1A, CHEK1, E2F1, MCM3, MCM4, MCM5, MCM6, MCM7, ORC1, PCNA, PLK1, RBL1, SKP2, TTK] |
| ubiquitin mediated proteolysis | 8 | [ANAPC4, ANAPC5, ANAPC7, BRCA1, CDC20, SKP2, UBE2C, UBE2D1] |
| homologous recombination | 3 | [BARD1, BRCA1, RAD51] |
| fanconi anemia pathway | 4 | [BRCA1, FANCD2, RAD51, UBE2T] |
| oocyte meiosis | 8 | [ANAPC4, ANAPC5, ANAPC7, BUB1, CCNE2, CDC20, FBXO5, PLK1] |
| p53 signaling pathway | 7 | [CASP3, CCNE2, CCNG2, CDK4, CDKN1A, CHEK1, RRM2] |
| Cellular senescence | 8 | [CCNA2, CCNE2, CDC25A, CDK4, CDKN1A, CHEK1, E2F1, RBL1] |
| progesterone-mediated oocyte maturation | 7 | [ANAPC4, ANAPC5, ANAPC7, BUB1, CCNA2, CDC25A, PLK1] |
| Hepatitis B | 8 | [CASP3, CCNA2, CCNE2, CDKN1A, E2F1, MMP9, PCNA, STAT3] |
| Human T-cell leukemia virus 1 infection | 11 | [ANAPC4, ANAPC5, ANAPC7, BUB1B, CCNA2, CCNE2, CDC20, CDK4, CDKN1A, CHEK1, E2F1] |
| Viral carcinogenesis | 10 | [CASP3, CCNA2, CCNE2, CDC20, CDK4, CDKN1A, CHEK1, RBL1, SKP2, STAT3] |
| pancreatic cancer | 5 | [CDK4, CDKN1A, E2F1, RAD51, STAT3] |
| prostate cancer | 4 | [CCNE2, CDKN1A, E2F1, MMP9] |
| melanoma | 3 | [CDK4, CDKN1A, E2F1] |
| bladder cancer | 4 | [CDK4, CDKN1A, E2F1, MMP9] |
| small cell lung cancer | 6 | [CASP3, CCNE2, CDK4, CDKN1A, E2F1, SKP2] |
| non-small lung cancer | 4 | [CDK4, CDKN1A, E2F1, STAT3] |
| 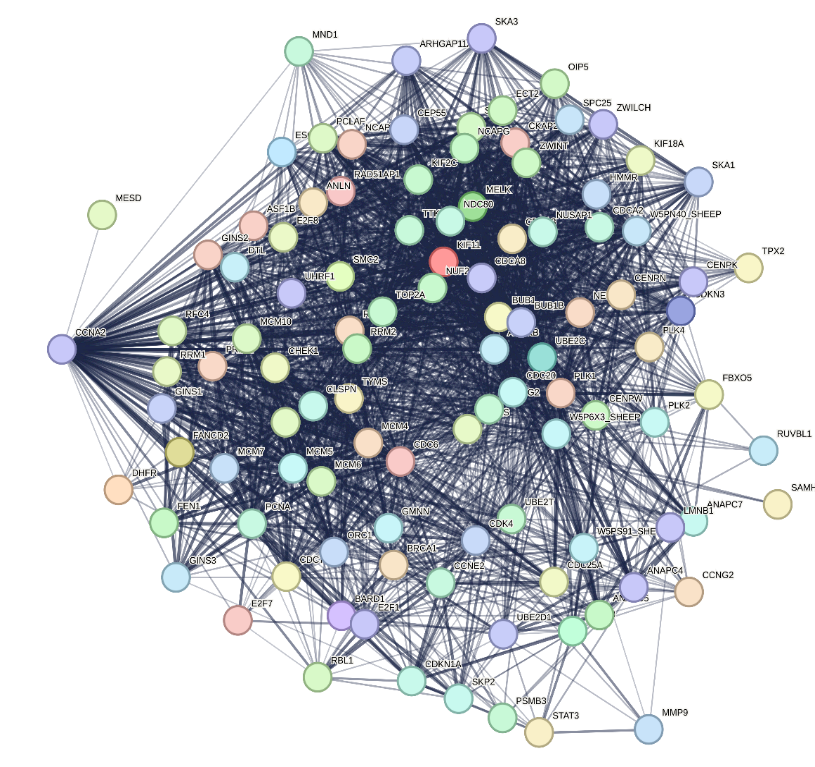 | | |

| **PLK1 (N1)** | | |
| --- | --- | --- |
| **Term** | **Nr. Genes** | **Associated Genes Found** |
| DNA replication | 5 | [MCM3, MCM4, MCM5, MCM6, MCM7] |
| Cell cycle | 24 | [ANAPC5, BUB1, BUB1B, CCNA2, CDC14A, CDC20, CDC23, CDC25A, CDC25C, CDC6, CDC7, CDK2, CDK4, CDKN1A, E2F1, MCM3, MCM4, MCM5, MCM6, MCM7, PLK1, SKP2, TTK, WEE1] |
| Ubiquitin mediated proteolysis | 6 | [ANAPC5, BRCA1, CDC20, CDC23, SKP2, UBE2D1] |
| homologous recombination | 5 | [BARD1, BLM, BRCA1, RAD51, RAD54L] |
| fanconi anemia pathway | 4 | [BLM, BRCA1, RAD51, UBE2T] |
| oocyte meiosis | 11 | [ANAPC5, BUB1, CDC20, CDC23, CDC25C, CDK2, CPEB4, FBXO5, PLK1, PPP2R5C, PPP2R5D] |
| progesterone-mediated oocyte maturation | 9 | [ANAPC5, BUB1, CCNA2, CDC23, CDC25A, CDC25C, CDK2, CPEB4, PLK1] |
| p53 signaling pathway | 4 | [CDK2, CDK4, CDKN1A, RRM2] |
| Cellular senescence | 7 | [CCNA2, CDC25A, CDK2, CDK4, CDKN1A, E2F1, HIPK2] |
| Human T-cell leukemia virus 1 infection | 10 | [ANAPC5, BUB1B, CCNA2, CDC20, CDC23, CDK2, CDK4, CDKN1A, E2F1, RANBP1] |
| pancreatic cancer | 4 | [CDK4, CDKN1A, E2F1, RAD51] |
| melanoma | 3 | [CDK4, CDKN1A, E2F1] |
| bladder cancer | 3 | [CDK4, CDKN1A, E2F1] |
| small cell lung cancer | 5 | [CDK2, CDK4, CDKN1A, E2F1, SKP2] |
| non-small lung cancer | 3 | [CDK4, CDKN1A, E2F1] |
| 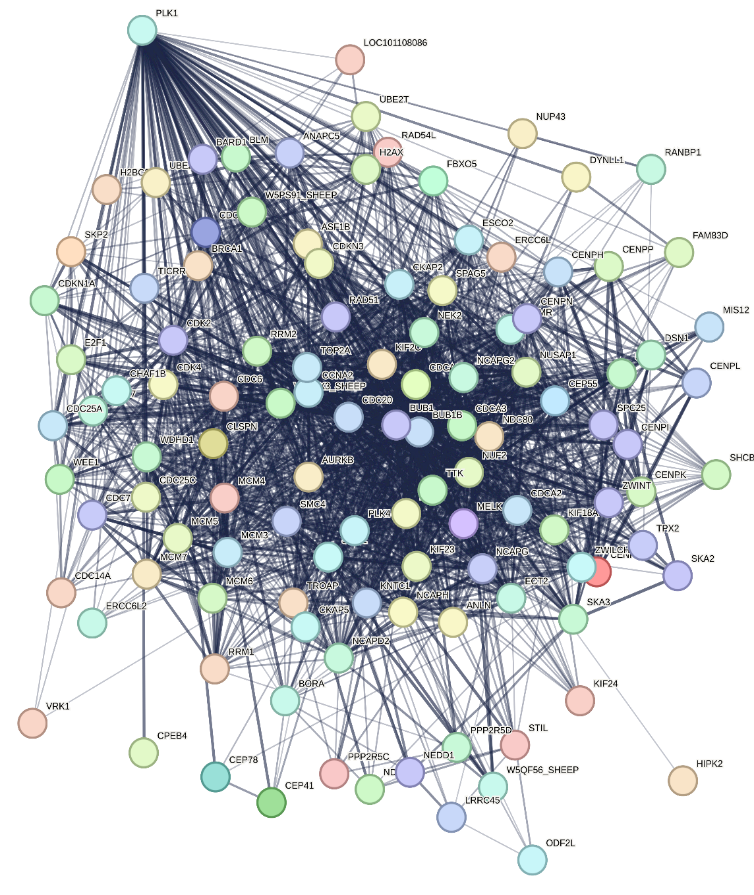 | | |

| **PLK1 (N2)** | | |
| --- | --- | --- |
| **Term** | **Nr. Genes** | **Associated Genes Found** |
| DNA replication | 5 | [MCM3, MCM4, MCM5, MCM6, MCM7] |
| Cell cycle | 22 | [ANAPC4, ANAPC5, ANAPC7, BUB1, BUB1B, CCNA2, CDC14A, CDC20, CDC25A, CDC6, CDC7, CDK4, CDKN1A, E2F1, MCM3, MCM4, MCM5, MCM6, MCM7, PLK1, SKP2, TTK] |
| Ubiquitin mediated proteolysis | 8 | [ANAPC4, ANAPC5, ANAPC7, BRCA1, CDC20, SKP2, UBE2C, UBE2D1] |
| Vasopressin-regulated water reabsorption | 3 | [DCTN5, DYNLL1, PRKACA] |
| Human T-cell leukemia virus 1 infection | 11 | [ANAPC4, ANAPC5, ANAPC7, BUB1B, CCNA2, CDC20, CDK4, CDKN1A, E2F1, PRKACA, RANBP1] |
| homologous recombination | 5 | [BARD1, BLM, BRCA1, RAD51, RAD54L] |
| fanconi anemia pathway | 4 | [BLM, BRCA1, RAD51, UBE2T] |
| oocyte meiosis | 11 | [ANAPC4, ANAPC5, ANAPC7, BUB1, CDC20, CPEB2, CPEB4, FBXO5, PLK1, PPP2R5C, PRKACA] |
| progesterone-mediated oocyte maturation | 10 | [ANAPC4, ANAPC5, ANAPC7, BUB1, CCNA2, CDC25A, CPEB2, CPEB4, PLK1, PRKACA] |
| p53 signaling pathway | 5 | [CASP3, CCNG2, CDK4, CDKN1A, RRM2] |
| pancreatic cancer | 4 | [CDK4, CDKN1A, E2F1, RAD51] |
| melanoma | 3 | [CDK4, CDKN1A, E2F1] |
| bladder cancer | 3 | [CDK4, CDKN1A, E2F1] |
| small cell lung cancer | 5 | [CASP3, CDK4, CDKN1A, E2F1, SKP2] |
| non-small lung cancer | 3 | [CDK4, CDKN1A, E2F1] |
| 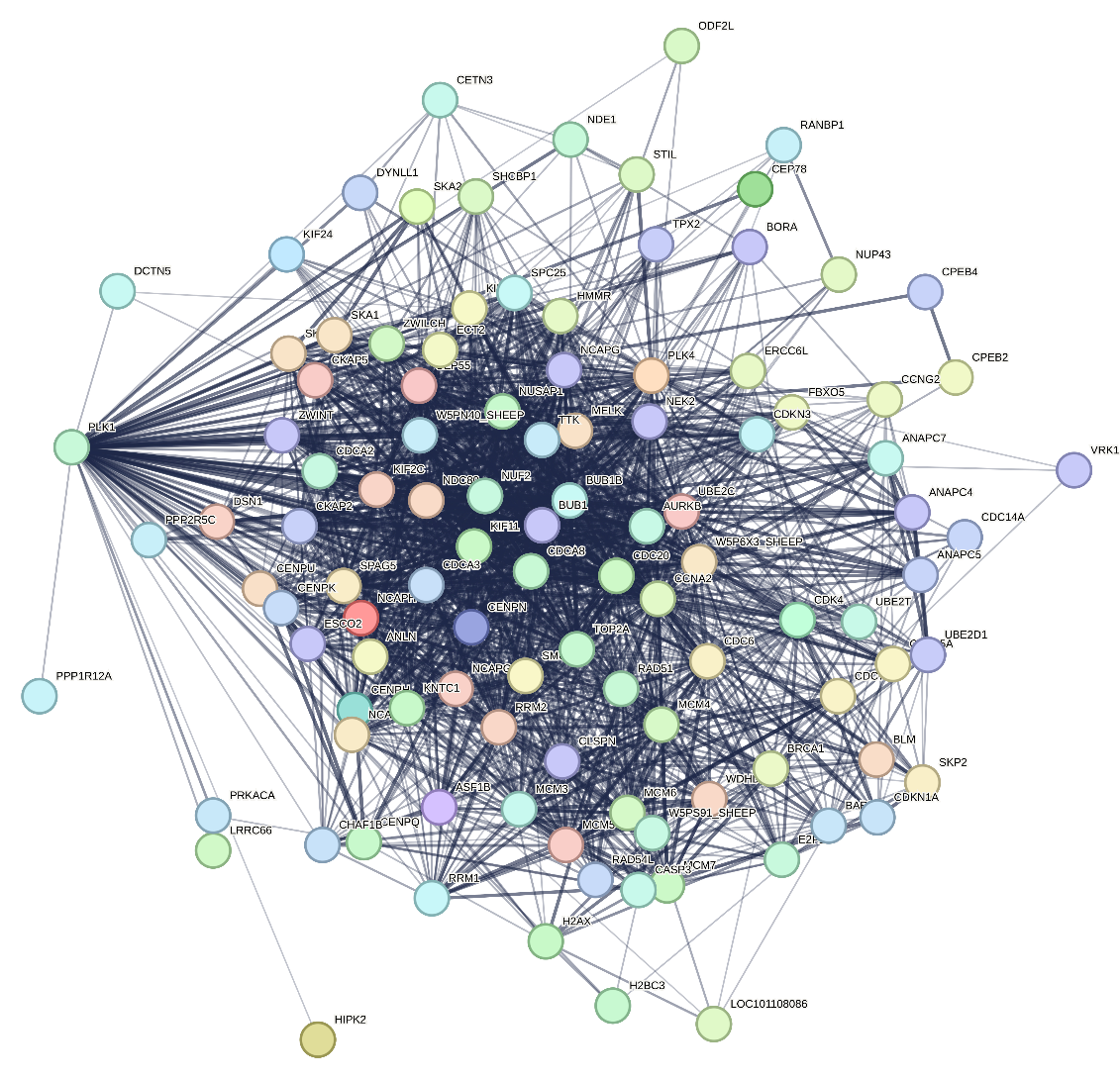 | | |
