# Supplementary material for: Tailor-made 3D in vitro maturation of early antral follicles uncovers cumulus-cell transcriptomic driver signature to predict oocyte competence
Source: Front Endocrinol (Lausanne). 2025 Oct 1;16:1629815. doi: 10.3389/fendo.2025.1629815 (PMC12520894; doi:10.3389/fendo.2025.1629815)
Supplement: Supplementary Table 1 — (Excel). The 12 centrality coefficients of each DEG of Network 1(MIIEndpoint- GVStartpoint) (Sheet: N1 MII-GV) and Network 2(GVEndpoint-GVStartpoint) (Sheet: N2 GV-GV) were scored using CytoHUBba. More in detail, they are closeness, degree, MCC, radiality, stress, MCN, DNMC, betweenness, clustering coefficient, eccentricity, bottleneck, and EPC. Network 1(MIIEndpoint- GVStartpoint) and Network 2(GVEndpoint-GVStartpoint) top 10 DEGs defined on each centrality coefficient score (Sheets: Top 10 N1 and N2 respectively). Venn diagram analysis of the top 10 DEGs of Network 1(MIIEndpoint- GVStartpoint) (Sheet: Ranking N1) and Network 2(GVEndpoint-GVStartpoint)(Sheet: Ranking N2) shows DEGs overlapping across the 12 algorithms. DEGs that are in the top 10 in at least 5 of the 6 algorithms are highlighted in bold. (Network1_Normalized) and (Network2_Normalized) include dataset values that have been statistically normalized using the standard score formula. [file DataSheet1.zip › Supplementary datasheets and tables/Supplementary Datasheet 6.docx]

**Supplementary Datasheet 6. Network 1(MII_Endpoint_- GV_Startpoint_) and Network 2(GV_Endpoint_-GV_Startpoint_) partner analysis and KEGG pathway analysis of distinctive HUBs.**

HUBS OF NETWORK 1

| **KIF11** | | |
| --- | --- | --- |
| **Term** | **Nr. Genes** | **Associated Genes Found** |
| Pyrimidine metabolism | 3 | [RRM1, RRM2, TYMS] |
| DNA replication | 7 | [MCM3, MCM4, MCM5, MCM6, MCM7, PCNA, POLA1] |
| Cell cycle | 17 | [BUB1, BUB1B, CCNA2, CDC20, CDC6, CDK2, CDK4, CHEK1, MCM3, MCM4, MCM5, MCM6, MCM7, ORC1, PCNA, PLK1, TTK] |
| p53 signaling pathway | 4 | [CDK2, CDK4, CHEK1, RRM2] |
| Homologous recombination | 4 | [BRIP1, RAD51, RAD54L, XRCC2] |
| Fanconi anemia pathway | 3 | [BRIP1, FANCI, RAD51] |
| Oocyte meiosis | 5 | [BUB1, CDC20, CDK2, FBXO5, PLK1] |
| Progesterone-mediated oocyte maturation | 4 | [BUB1, CCNA2, CDK2, PLK1] |
| **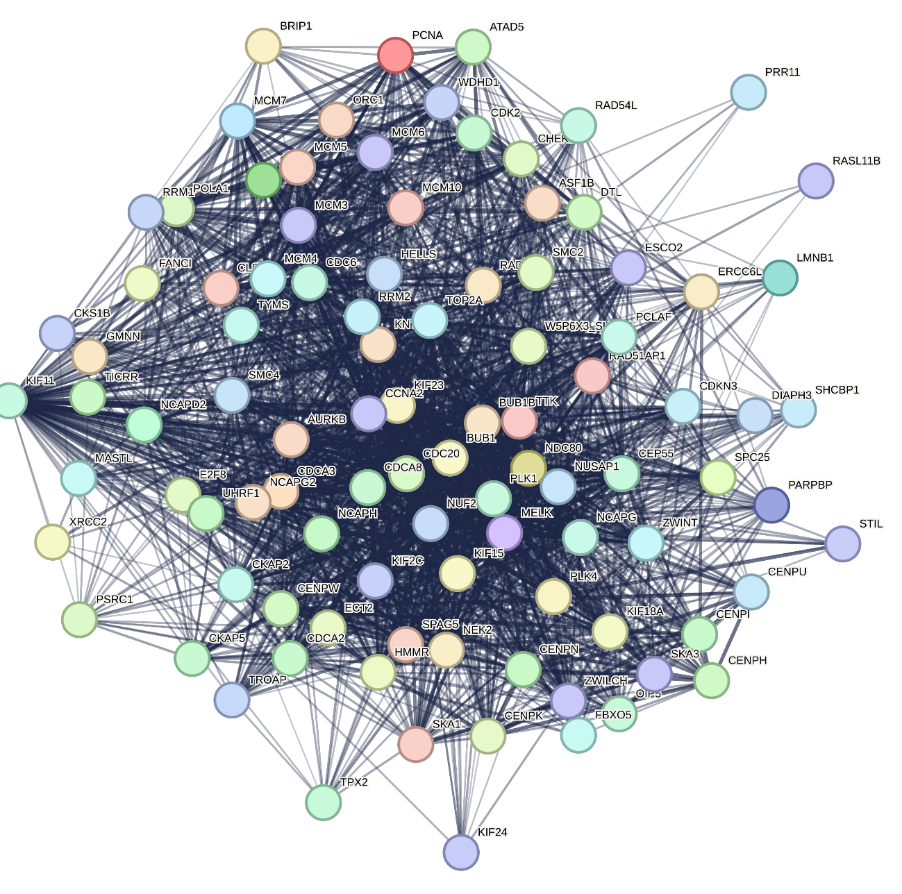** | | |

| **CDC6** | | |
| --- | --- | --- |
| **KEGG** | **Nr. Genes** | **Associated Genes Found** |
| Pyrimidine metabolism | 3 | [RRM1, RRM2, TYMS] |
| Cell cycle | 29 | [ANAPC5, BUB1, BUB1B, CCNA2, CCNE2, CDC14A, CDC20, CDC23, CDC25A, CDC25C, CDC6, CDC7, CDK2, CDK4, CDKN1A, CHEK1, E2F1, MCM3, MCM4, MCM5, MCM6, MCM7, ORC1, PCNA, PLK1, RBL1, SKP2, TTK, WEE1] |
| Homologous recombination | 4 | [BRCA1, BRCC3, RAD51, RPA2] |
| Fanconi anemia pathway | 5 | [BRCA1, RAD51, REV3L, RPA2, UBE2T] |
| Oocyte meiosis | 8 | [ANAPC5, BUB1, CCNE2, CDC20, CDC23, CDC25C, CDK2, PLK1] |
| Progesterone-mediated oocyte maturation | 8 | [ANAPC5, BUB1, CCNA2, CDC23, CDC25A, CDC25C, CDK2, PLK1] |
| Human T-cell leukemia virus 1 infection | 11 | [ANAPC5, BUB1B, CCNA2, CCNE2, CDC20, CDC23, CDK2, CDK4, CDKN1A CHEK1, E2F1] |
| DNA replication | 15 | [FEN1, MCM3, MCM4, MCM5, MCM6, MCM7, PCNA, POLA1, POLA2, POLE, POLE2, PRIM1, RFC2, RFC4, RPA2] |
| Base excision repair | 4 | [FEN1, PCNA, POLE, POLE2] |
| Nucleotide excision repair | 6 | [PCNA, POLE, POLE2, RFC2, RFC4, RPA2] |
| Mismatch repair | 4 | [PCNA, RFC2, RFC4, RPA2] |
| Oocyte meiosis | 8 | [ANAPC5, BUB1, CCNE2, CDC20, CDC23, CDC25C, CDK2, PLK1] |
| p53 signaling pathway | 6 | [CCNE2, CDK2, CDK4, CDKN1A, CHEK1, RRM2] |
| Cellular senescence | 9 | [CCNA2, CCNE2, CDC25A, CDK2, CDK4, CDKN1A, CHEK1, E2F1, RBL1] |
| Human T-cell leukemia virus 1 infection | 11 | [ANAPC5, BUB1B, CCNA2, CCNE2, CDC20, CDC23, CDK2, CDK4, CDKN1A, CHEK1, E2F1] |
| Pancreatic cancer | 4 | [CDK4, CDKN1A, E2F1, RAD51] |
| Prostate cancer | 4 | [CCNE2, CDK2, CDKN1A, E2F1] |
| Melanoma | 3 | [CDK4, CDKN1A, E2F1] |
| Bladder cancer | 3 | [CDK4, CDKN1A, E2F1] |
| Small cell lung cancer | 6 | [CCNE2, CDK2, CDK4, CDKN1A, E2F1, SKP2] |
| Non-small cell lung cancer | 3 | [CDK4, CDKN1A E2F1] |
| **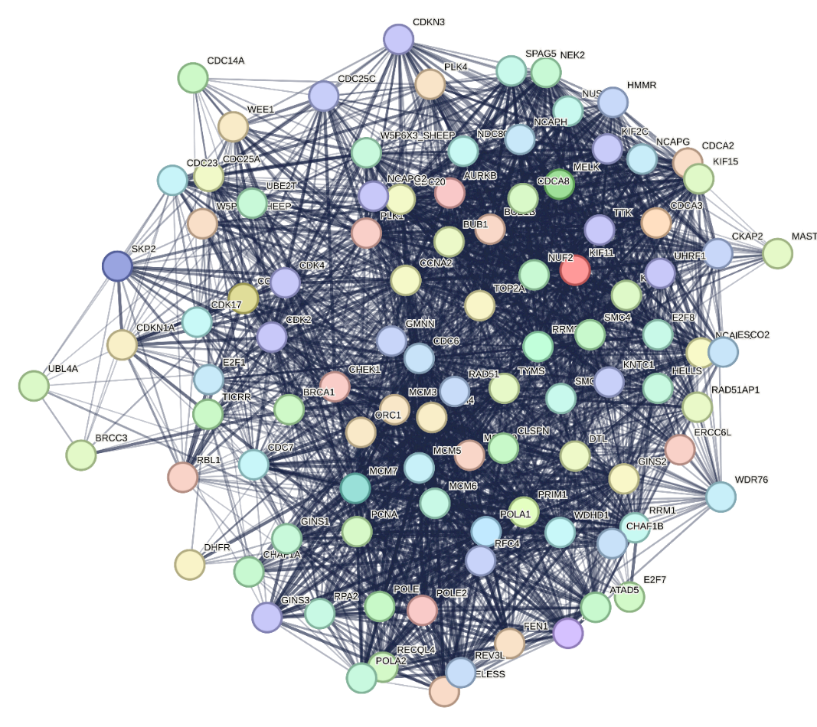** | | |

HUBS OF NETWORK 2

| **CASP3** | | |
| --- | --- | --- |
| **Term** | **Nr. Genes** | **Associated Genes Found** |
| HIF-1 signaling pathway | 6 | [CDKN1A, EDN1, HK2, NOS2, SERPINE1, STAT3] |
| FoxO signaling pathway | 11 | [BNIP3, CCND2, CDKN1A, FOXO3, IRS1, PLK1, SIRT1, SKP2, SMAD3, STAT3, TNFSF10] |
| Mitophagy | 4 | [BNIP3, E2F1, FOXO3, SQSTM1] |
| Apoptosis | 8 | [CASP2, CASP3, CTSS, DDIT3, LMNB1, LMNB2, PARP1, TNFSF10] |
| Progesterone-mediated oocyte maturation | 4 | [CCNA2, CDC25A, PLK1, PRKACA] |
| Thyroid hormone synthesis | 3 | [GPX2, GPX3, PRKACA] |
| Type II diabetes mellitus | 3 | [HK2, IRS1, PRKCD] |
| Legionellosis | 3 | [BNIP3, CASP3, HSPA6] |
| Amoebiasis | 4 | [CASP3, NOS2, PRKACA, VCL] |
| MicroRNAs in cancer | 12 | [BMF, BRCA1, CASP3, CCND2, CDC25A, CDKN1A, E2F1, IRS1, MDM4, MMP9, SIRT1, STAT3] |
| Longevity regulating pathway | 4 | [FOXO3, IRS1, PRKACA, SIRT1] |
| Longevity regulating pathway | 5 | [FOXO3, HSPA6, IRS1, PRKACA, SIRT1] |
| AGE-RAGE signaling pathway in diabetic complications | 8 | [CASP3, CDK4, EDN1, IL1A, PRKCD, SERPINE1, SMAD3, STAT3] |
| Inflammatory bowel disease (IBD) | 3 | [IL1A, SMAD3, STAT3] |
| Cell cycle | 10 | [CCNA2, CCND2, CDC20, CDC25A, CDK4, CDKN1A, E2F1, PLK1, SKP2, SMAD3] |
| Cellular senescence | 12 | [CCNA2, CCND2, CDC25A, CDK4, CDKN1A, E2F1, FOXO3, IL1A, SERPINE1, SIRT1, SMAD3, SQSTM1] |
| Epstein-Barr virus infection | 11 | [CASP3, CCNA2, CCND2, CDK4, CDKN1A, CXCL10, E2F1, JAK1, SKP2, STAT2, STAT3] |
| Viral carcinogenesis | 11 | [CASP3, CCNA2, CCND2, CDC20, CDK4, CDKN1A, JAK1, PRKACA, SCIN, SKP2, STAT3] |
| Cell cycle | 10 | [CCNA2, CCND2, CDC20, CDC25A, CDK4, CDKN1A, E2F1, PLK1, SKP2, SMAD3] |
| p53 signaling pathway | 6 | [CASP3, CCND2, CDK4, CDKN1A, MDM4, SERPINE1] |
| Hepatitis C | 8 | [CASP3, CDK4, CDKN1A, CXCL10, E2F1, JAK1, STAT2, STAT3] |
| Hepatitis B | 9 | [CASP3, CCNA2, CDKN1A, E2F1, JAK1, MMP9, SMAD3, STAT2, STAT3] |
| Measles | 8 | [CASP3, CCND2, CDK4, HSPA6, IL1A, JAK1, STAT2, STAT3] |
| Epstein-Barr virus infection | 11 | [CASP3, CCNA2, CCND2, CDK4, CDKN1A, CXCL10, E2F1, JAK1, SKP2, STAT2, STAT3] |
| Viral carcinogenesis | 11 | [CASP3, CCNA2, CCND2, CDC20, CDK4, CDKN1A, JAK1, PRKACA, SCIN, SKP2, STAT3] |
| Pancreatic cancer | 7 | [CDK4, CDKN1A, E2F1, JAK1, RAD51, SMAD3, STAT3] |
| Melanoma | 3 | [CDK4, CDKN1A, E2F1] |
| Bladder cancer | 4 | [CDK4, CDKN1A, E2F1, MMP9] |
| Small cell lung cancer | 6 | [CASP3, CDK4, CDKN1A, E2F1, NOS2, SKP2] |
| Non-small cell lung cancer | 5 | [CDK4, CDKN1A, E2F1, FOXO3, STAT3] |
| **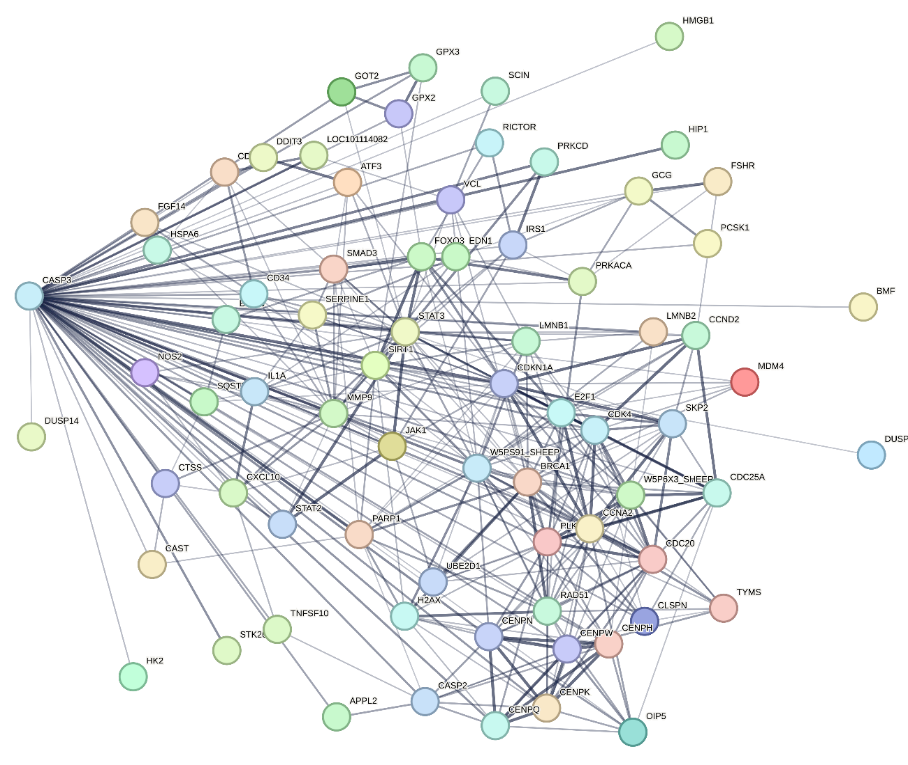** | | |
