# Supplementary material for: Tailor-made 3D in vitro maturation of early antral follicles uncovers cumulus-cell transcriptomic driver signature to predict oocyte competence
Source: Front Endocrinol (Lausanne). 2025 Oct 1;16:1629815. doi: 10.3389/fendo.2025.1629815 (PMC12520894; doi:10.3389/fendo.2025.1629815)
Supplement: Supplementary Table 1 — (Excel). The 12 centrality coefficients of each DEG of Network 1(MIIEndpoint- GVStartpoint) (Sheet: N1 MII-GV) and Network 2(GVEndpoint-GVStartpoint) (Sheet: N2 GV-GV) were scored using CytoHUBba. More in detail, they are closeness, degree, MCC, radiality, stress, MCN, DNMC, betweenness, clustering coefficient, eccentricity, bottleneck, and EPC. Network 1(MIIEndpoint- GVStartpoint) and Network 2(GVEndpoint-GVStartpoint) top 10 DEGs defined on each centrality coefficient score (Sheets: Top 10 N1 and N2 respectively). Venn diagram analysis of the top 10 DEGs of Network 1(MIIEndpoint- GVStartpoint) (Sheet: Ranking N1) and Network 2(GVEndpoint-GVStartpoint)(Sheet: Ranking N2) shows DEGs overlapping across the 12 algorithms. DEGs that are in the top 10 in at least 5 of the 6 algorithms are highlighted in bold. (Network1_Normalized) and (Network2_Normalized) include dataset values that have been statistically normalized using the standard score formula. [file DataSheet1.zip › Supplementary datasheets and tables/Supplementary Datasheet 1.docx]

**Supplementary Datasheet 1. Computed topological parameters for Network 1(MII_Endpoint_- GV_Startpoint_) and Network 2(GV_Endpoint_-GV_Startpoint_)**

Network 1(MII_Endpoint_- GV_Startpoint_) topological parameters.

| **Statistics** | **Network 1(MII_Endpoint_- GV_Startpoint_)** |
| --- | --- |
| **Number of nodes** | 1144 |
| **Number of edges** | 7388 |
| **Avg. number of neighbors** | 13 949 |
| **Network diameter** | 9 |
| **Characteristic path length** | 3.791 |
| **Clustering coefficient** | 0.238 |
| **Connected components** | 84 |

Network 2(GV_Endpoint_-GV_Startpoint_) topological parameters.

| **Statistics** | **Network 2(GV_Endpoint_-GV_Startpoint_)** |
| --- | --- |
| **Number of nodes** | 1206 |
| **Number of edges** | 7617 |
| **Avg. number of neighbors** | 13 291 |
| **Network diameter** | 9 |
| **Characteristic path length** | 3.761 |
| **Clustering coefficient** | 0.224 |
| **Connected components** | 60 |
