# Supplementary material for: Tailor-made 3D in vitro maturation of early antral follicles uncovers cumulus-cell transcriptomic driver signature to predict oocyte competence
Source: Front Endocrinol (Lausanne). 2025 Oct 1;16:1629815. doi: 10.3389/fendo.2025.1629815 (PMC12520894; doi:10.3389/fendo.2025.1629815)
Supplement: Supplementary Table 1 — (Excel). The 12 centrality coefficients of each DEG of Network 1(MIIEndpoint- GVStartpoint) (Sheet: N1 MII-GV) and Network 2(GVEndpoint-GVStartpoint) (Sheet: N2 GV-GV) were scored using CytoHUBba. More in detail, they are closeness, degree, MCC, radiality, stress, MCN, DNMC, betweenness, clustering coefficient, eccentricity, bottleneck, and EPC. Network 1(MIIEndpoint- GVStartpoint) and Network 2(GVEndpoint-GVStartpoint) top 10 DEGs defined on each centrality coefficient score (Sheets: Top 10 N1 and N2 respectively). Venn diagram analysis of the top 10 DEGs of Network 1(MIIEndpoint- GVStartpoint) (Sheet: Ranking N1) and Network 2(GVEndpoint-GVStartpoint)(Sheet: Ranking N2) shows DEGs overlapping across the 12 algorithms. DEGs that are in the top 10 in at least 5 of the 6 algorithms are highlighted in bold. (Network1_Normalized) and (Network2_Normalized) include dataset values that have been statistically normalized using the standard score formula. [file DataSheet1.zip › Supplementary datasheets and tables/Supplementary Datasheet 3.docx]

**Supplementary Datasheet 3. Comparative meiotic and developmental competence of FEO *in vitro* matured oocytes derived from EAfs and SAfs.**

**A**

| Follicle category | Healthy oocytes  (n°) | Oocyte Nuclear Stage | | |
| --- | --- | --- | --- | --- |
|  |  | GV  (%; SD) | GVBD/MI | MII |
| EA -hCG | 40 | 100% | -- | -- |
| Small A- hCG | 38 | 100% | -- | -- |
| EA +hCG | 160 | 10.3±1.6 | 23.1±2.5 | 66.6± 2.7 |
| Small A+ hCG | 160 | 8.5±1 | 16.8 2±2.5 | 74.7±3.5 |

**B**

|  | Healthy oocytes  (n°) | Parthenogenetic Embryo Development | | | |
| --- | --- | --- | --- | --- | --- |
|  |  | Uncleaved  (%; SD) | PN  (%; SD) | <8 nuclei  (%; SD) | > 8 nuclei  (%; SD) |
| EA | 40 | 29.9±1 | 29±4^a^ | 13.6±3 | 27.5±5.5^a^ |
| Small A | 40 | 32±2 | 15.3±4 | 15±6 | 37.7±3.2 |

**C**

|  | Healthy oocytes  (n°) | Post-Fertilization Embryo Development | | | |
| --- | --- | --- | --- | --- | --- |
|  |  | Uncleaved  (%; SD) | Fertilization rate  (%; SD) | | Blastocyst rate  (%; SD) |
| EA | 66 | 31.8±4.5 | | 68.8±5.1 | 9.2±3.9 |
| Small A | 79 | 25±5 | 75±3 | | 15.8±5.5 |

**Evaluation of Meiotic and Developmental Competence in EAfs and SAfs**. **(A)** FEO in vitro maturation was conducted following the protocol described in [14], where EA follicles (average diameter: 400±26 µm) were cultured with hCG (25 IU/mL) in the presence of ovarian surface epithelial cells. Follicular cells (FCs) were isolated after a 24-hour maturation period based on the nuclear status of the enclosed oocytes, categorized as either MII or GV. MII oocytes were subsequently used for parthenogenetic activation and IVF. **(B)** The cytoplasmic maturation of MII oocytes, confirmed by the extrusion of the polar body, was assessed through parthenogenetic activation. This approach enabled an evaluation of oocyte quality independently of fertilization. Embryo cleavage was examined 72 hours post-activation with ethanol. **(C)** The developmental potential of in vitro matured FEO oocytes was analyzed following IVF. Fertilization rates were determined by the proportion of embryos that cleaved by day 2, while blastocyst formation was assessed at day 8 relative to the number of cleaved embryos recorded on day 2.
