# Supplementary material for: Tailor-made 3D in vitro maturation of early antral follicles uncovers cumulus-cell transcriptomic driver signature to predict oocyte competence
Source: Front Endocrinol (Lausanne). 2025 Oct 1;16:1629815. doi: 10.3389/fendo.2025.1629815 (PMC12520894; doi:10.3389/fendo.2025.1629815)
Supplement: Supplementary Table 1 — (Excel). The 12 centrality coefficients of each DEG of Network 1(MIIEndpoint- GVStartpoint) (Sheet: N1 MII-GV) and Network 2(GVEndpoint-GVStartpoint) (Sheet: N2 GV-GV) were scored using CytoHUBba. More in detail, they are closeness, degree, MCC, radiality, stress, MCN, DNMC, betweenness, clustering coefficient, eccentricity, bottleneck, and EPC. Network 1(MIIEndpoint- GVStartpoint) and Network 2(GVEndpoint-GVStartpoint) top 10 DEGs defined on each centrality coefficient score (Sheets: Top 10 N1 and N2 respectively). Venn diagram analysis of the top 10 DEGs of Network 1(MIIEndpoint- GVStartpoint) (Sheet: Ranking N1) and Network 2(GVEndpoint-GVStartpoint)(Sheet: Ranking N2) shows DEGs overlapping across the 12 algorithms. DEGs that are in the top 10 in at least 5 of the 6 algorithms are highlighted in bold. (Network1_Normalized) and (Network2_Normalized) include dataset values that have been statistically normalized using the standard score formula. [file DataSheet1.zip › Supplementary datasheets and tables/Supplementary Datasheet 2.docx]

**Supplementary Datasheet 2. RT – qPCR primer sequences for principal Driver Gene validation.**

| **Gene name** | **Fw/Rv** | **Sequence 5’-3’** | **Tm (°C)** |
| --- | --- | --- | --- |
| **oaHS6ST2** | Fw | ACACATCTAAGAATGGGAAGAACT | 57.62 |
|  | Rv | CAGCTCTTCAGAGGTTGGGG | 60.04 |
| **oaCDC6** | Fw | TCAGATCTCAGGAAGCTGTGC | 57.62 |
|  | Rv | TTTGTCCACTGTCCGAGACG | 55.00 |
| **oaERO1A** | Fw | AAAAGACCTTTAAATCCTTTGGCTT | 57.89 |
|  | Rv | TCTACGCAGAGACCTTCTAGC | 58.71 |
| **oaCASP3** | Fw | ACGTTGTGGCTGAACGTAAA | 58.35 |
|  | Rv | CCGGAGTCCACTGATTTGCT | 60.04 |
| **oaSEMA3A** | Fw | ATATATGTGGACAGAATTGAGGGTA | 57.13 |
|  | Rv | GACGCTGTTGGTCCTATGCT | 60.11 |
