# Supplementary material for: Tailor-made 3D in vitro maturation of early antral follicles uncovers cumulus-cell transcriptomic driver signature to predict oocyte competence
Source: Front Endocrinol (Lausanne). 2025 Oct 1;16:1629815. doi: 10.3389/fendo.2025.1629815 (PMC12520894; doi:10.3389/fendo.2025.1629815)
Supplement: Supplementary Table 1 — (Excel). The 12 centrality coefficients of each DEG of Network 1(MIIEndpoint- GVStartpoint) (Sheet: N1 MII-GV) and Network 2(GVEndpoint-GVStartpoint) (Sheet: N2 GV-GV) were scored using CytoHUBba. More in detail, they are closeness, degree, MCC, radiality, stress, MCN, DNMC, betweenness, clustering coefficient, eccentricity, bottleneck, and EPC. Network 1(MIIEndpoint- GVStartpoint) and Network 2(GVEndpoint-GVStartpoint) top 10 DEGs defined on each centrality coefficient score (Sheets: Top 10 N1 and N2 respectively). Venn diagram analysis of the top 10 DEGs of Network 1(MIIEndpoint- GVStartpoint) (Sheet: Ranking N1) and Network 2(GVEndpoint-GVStartpoint)(Sheet: Ranking N2) shows DEGs overlapping across the 12 algorithms. DEGs that are in the top 10 in at least 5 of the 6 algorithms are highlighted in bold. (Network1_Normalized) and (Network2_Normalized) include dataset values that have been statistically normalized using the standard score formula. [file DataSheet1.zip › Supplementary datasheets and tables/Supplementary Datasheet 5.docx]

**Supplementary Datasheet 5. Network 1(MII_Endpoint_- GV_Startpoint_) and Network 2(GV_Endpoint_-GV_Startpoint_) partner analysis and KEGG pathways analysis of Highly – modulated DEGs.**

OUTLIERS OF NETWORK 1

| EFHD1 |
| --- |
| KEGG |
| **Purine metabolism** |
| **Pyrimidine metabolism** |
| **Metabolic pathways** |
| **Nicotinate and Nicotinamide metabolism** |
| **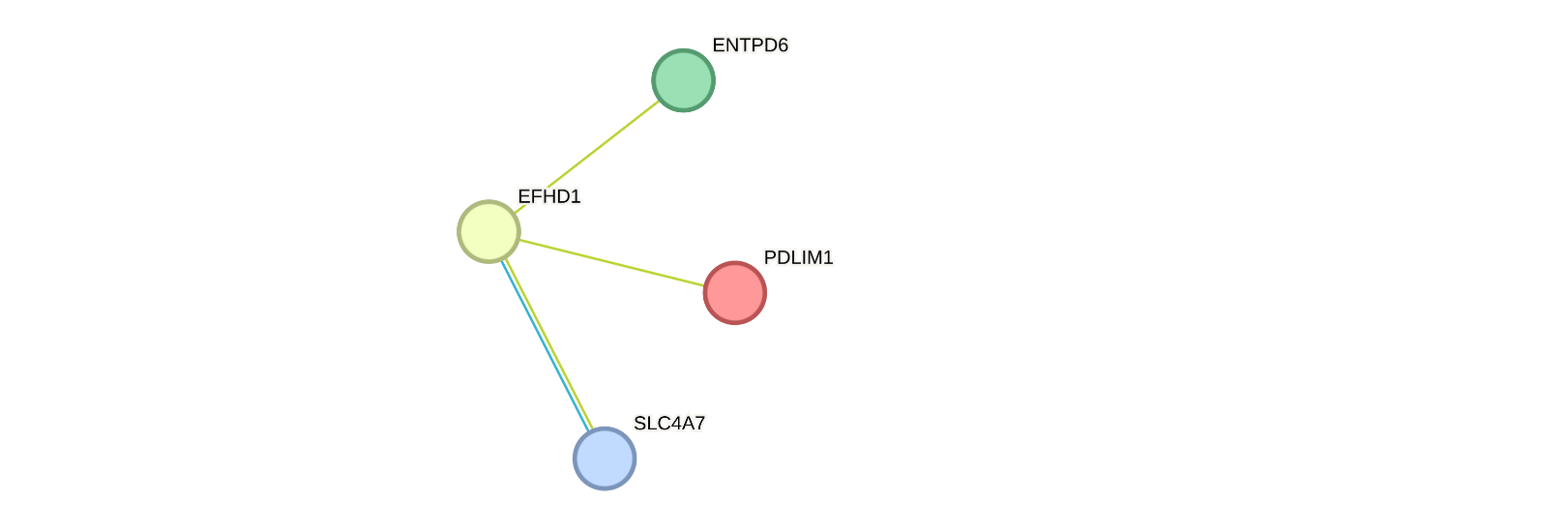** |

| **HS6ST2** |
| --- |
| KEGG |
| **Glycosaminoglycan biosynthesis- heparan sulfate/heparin** |
| **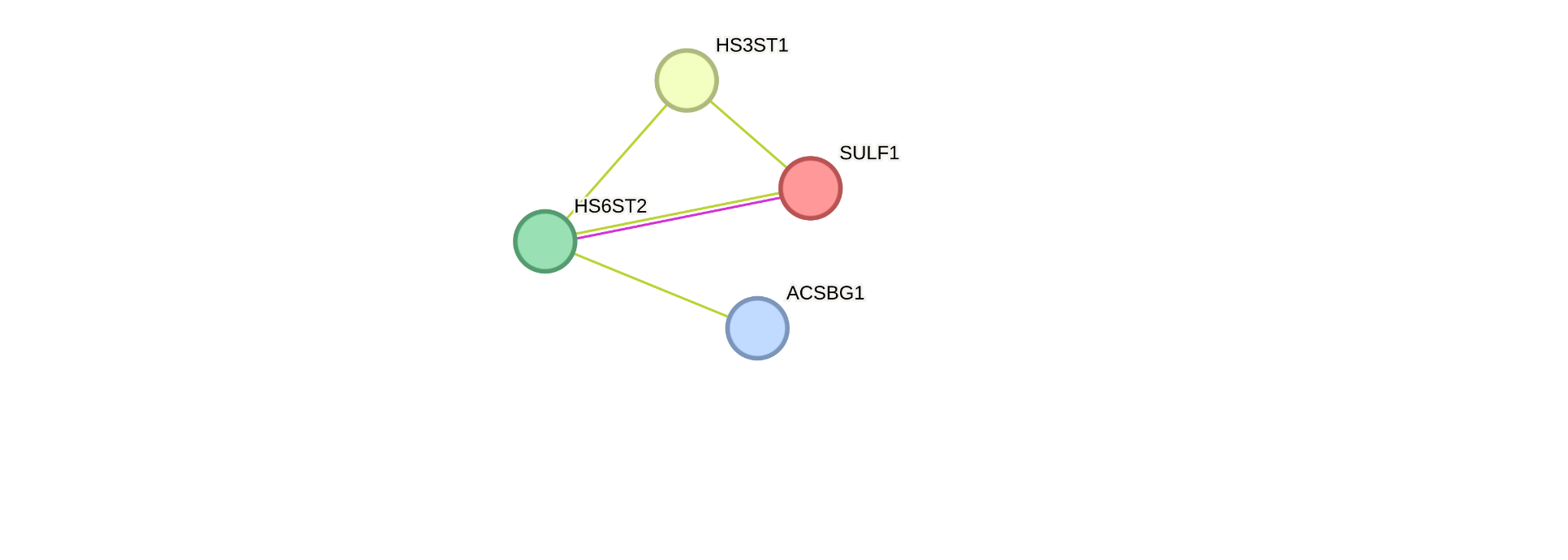** |

| **SLC35G1** |
| --- |
| **KEGG** |
| **N/A** |
| **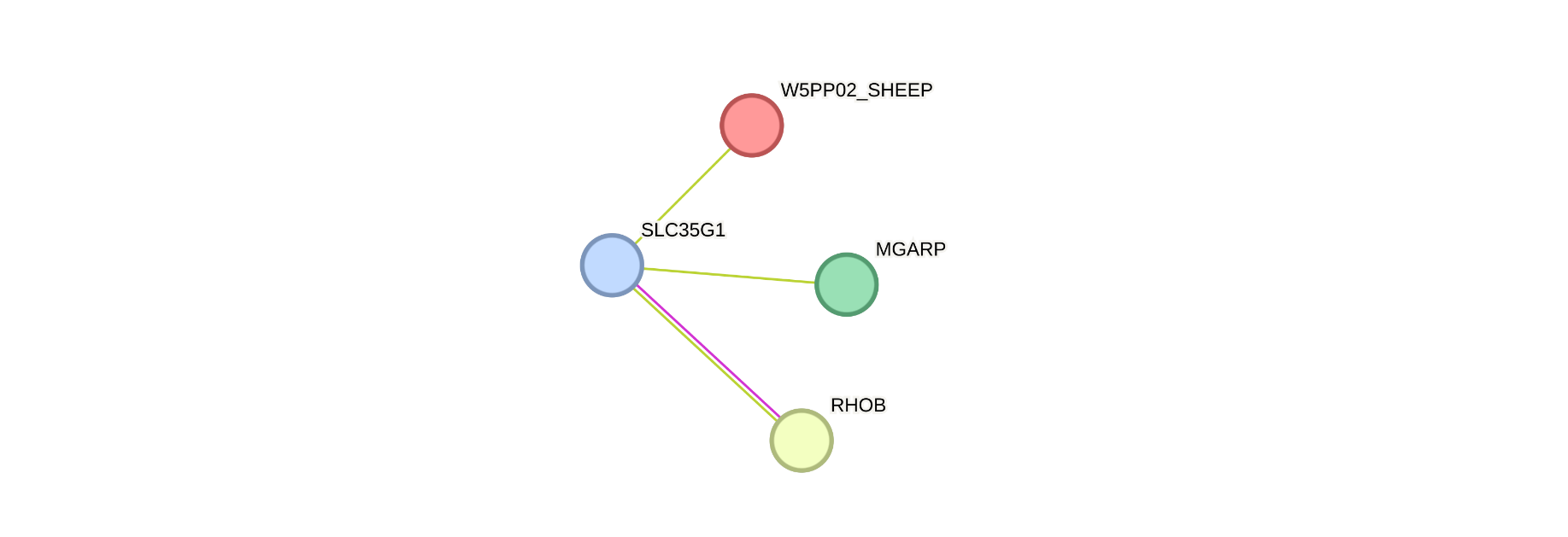** |

OUTLIERS OF NETWORK 2

| HBA1 |
| --- |
| KEGG |
| N/A |

| TKDP5 |
| --- |
| KEGG |
| N/A |

| CALCRL |
| --- |
| KEGG |
| **Neuroactive ligand-receptor interaction** |
| **Circadian entrainment** |
| **Glutamatergic synapse** |
| **Dopaminergic synapse** |
| **Retrograde endocannabinoid signaling** |
| **cAMP signaling pathway** |
| **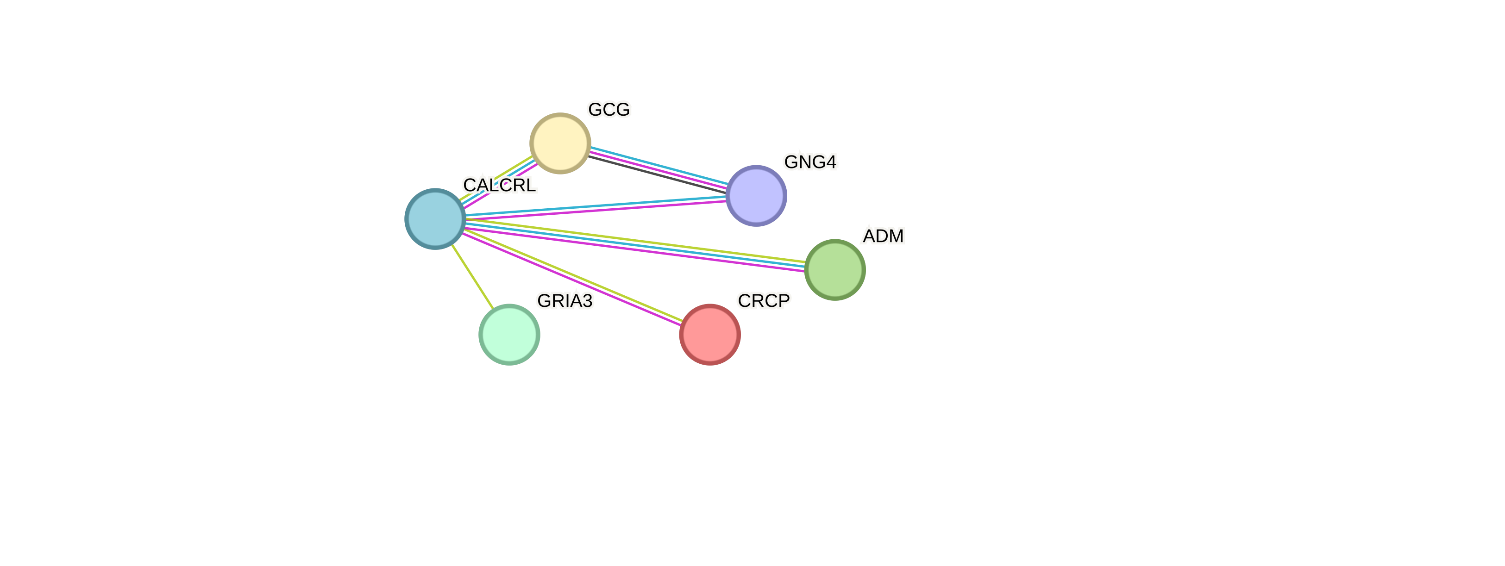** |

| **ELOVL6** |
| --- |
| **KEGG** |
| **Fatty acid metabolism** |
| **Biosynthesis of unsaturated fatty acids** |
| **Fatty acid elongation** |
| **PPAR signaling pathway** |
| **Fatty acid degradation** |
| **Metabolic pathways** |
| **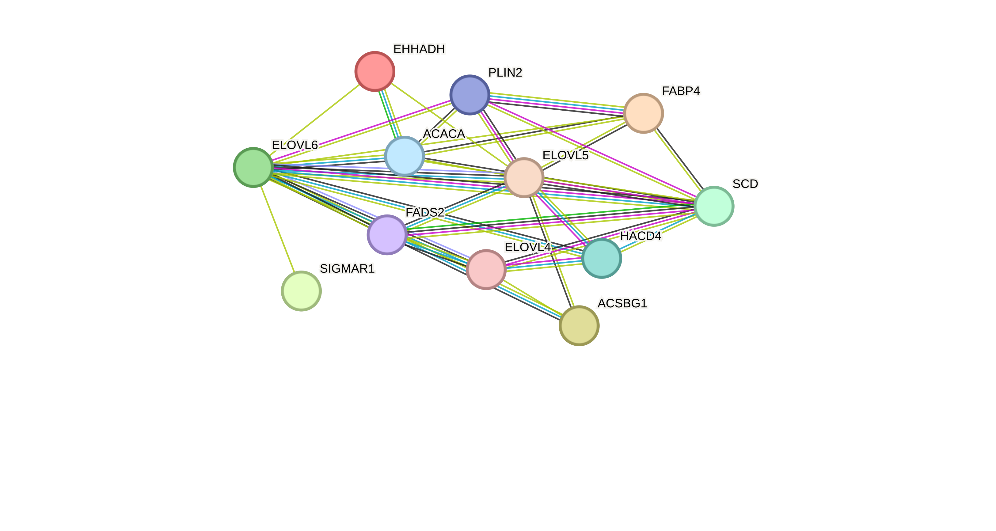** |

| **ERO1A** |
| --- |
| **KEGG** |
| **Protein processing in endoplasmic reticulum** |
| **Antigen processing and presentation** |
| **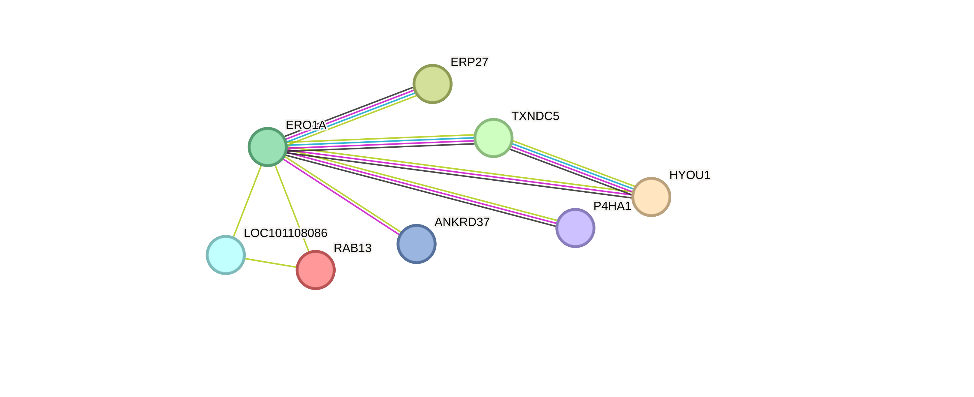** |

| **SLC39A8** |
| --- |
| **KEGG** |
| **Sphingolipid metabolism** |
| **Sphingolipid signaling pathway** |
| **Metabolic pathways** |
| **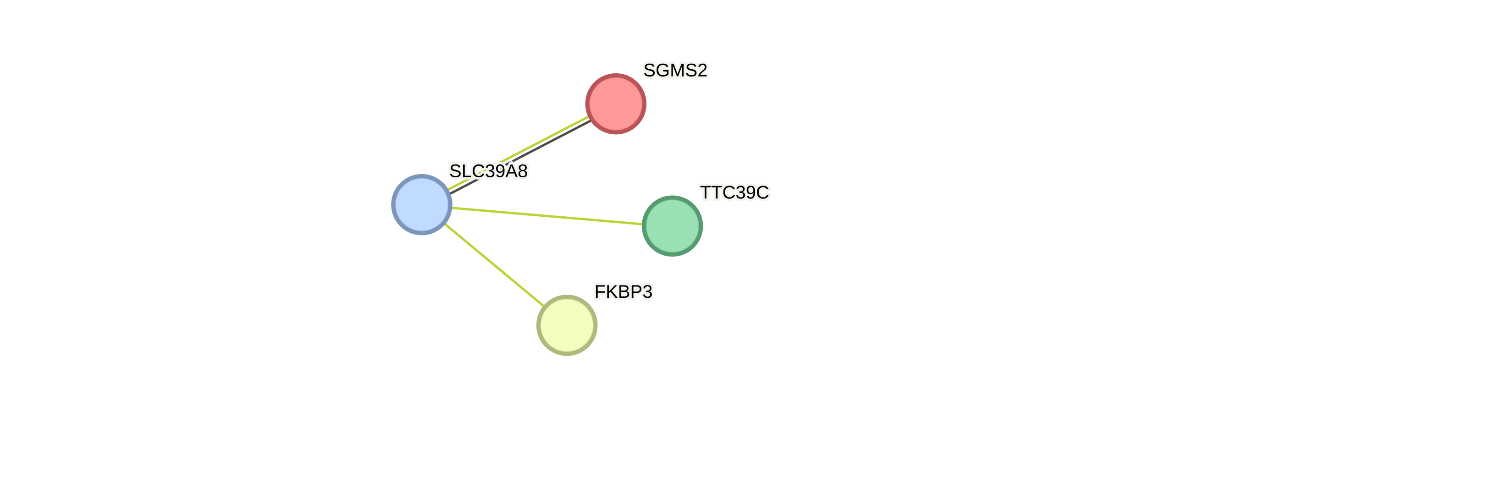** |

SHARED HIGHLY MODULATED DEGS BETWEEN N1 AND N2

| **INHA (N1)** | | |
| --- | --- | --- |
| **Term** | **Nr. Genes** | **Associated Genes Found** |
| Hormonal Regulation | 3 | INHA, FST, INHBA |
| Steroidogenesis and metabolism | 2 | CYP11A1, HSD17B1 |
| Transcriptional Regulation | 1 | FOXL2 |
| Receptor and Signaling Pathways | 1 | FSHR |
| Structural role | 1 | ODF2L |
| **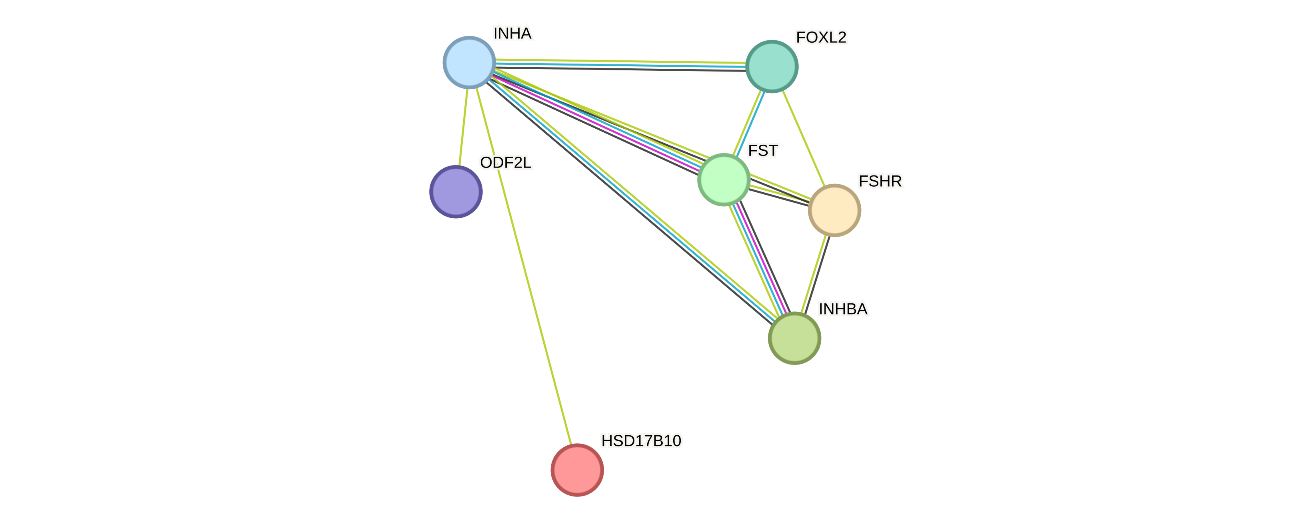** | | |
| **INHA (N2)** | | |
| **Term** | **Nr. Genes** | **Associated Genes Found** |
| Hormonal Regulation | 3 | INHA. FST, INHBA |
| Steroidogenesis and metabolism | 3 | CYP11A1, CYP19, HSD17B1 |
| Transcriptional Regulation | 1 | FOXL2 |
| Receptor and Signaling pathway | 2 | FSHR, NTRK1 |
| Structural Role | 1 | ODF2L |
| **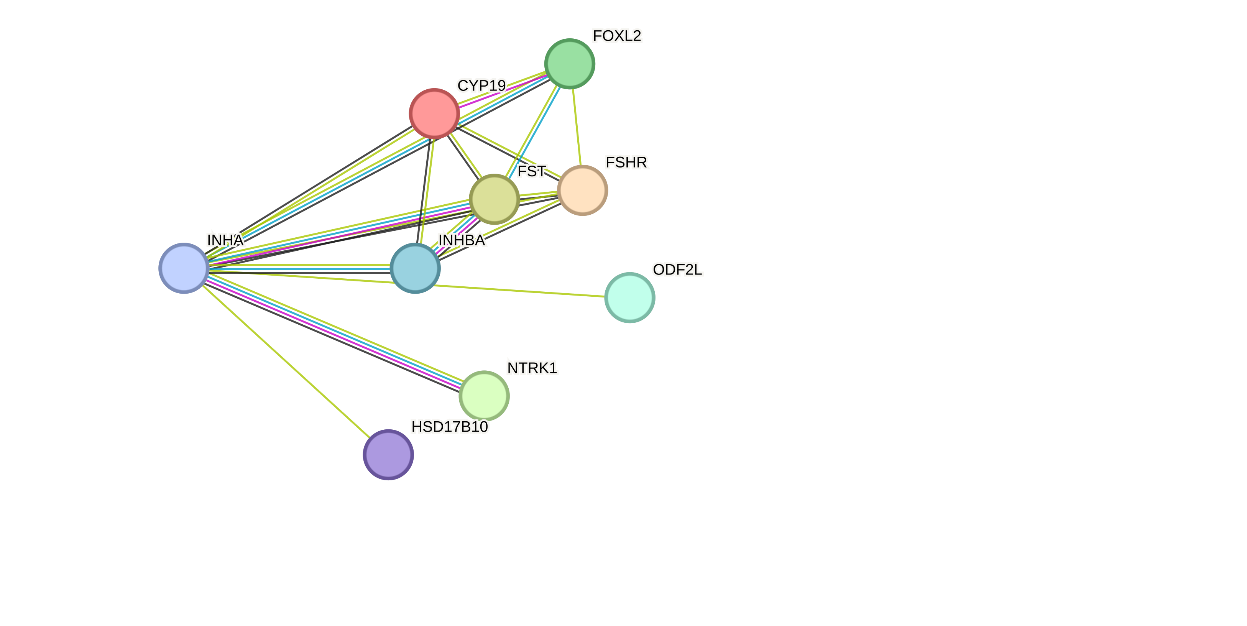** | | |

| **GFRA3 (N1)** | | |
| --- | --- | --- |
| **Term** | **Nr. Genes** | **Associated Genes Found** |
| Receptor and Signaling Pathways | 1 | GFRA3 |
| Cell Cycle Regulation | 2 | CDC23, STIL |
| Structural role | 1 | NEFM |
| **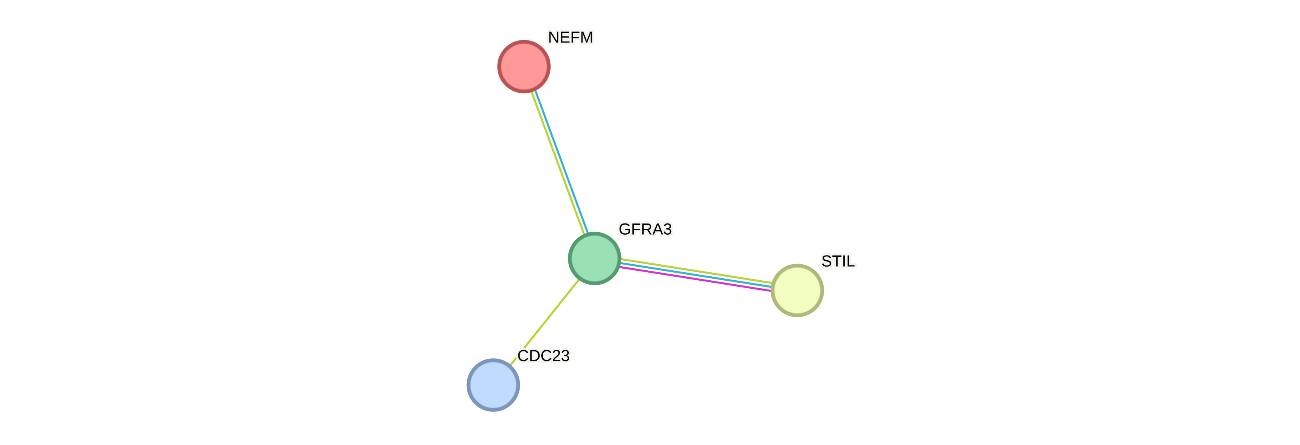** | | |

| **GFRA3 (N2)** | | |
| --- | --- | --- |
| **Term** | **Nr. Genes** | **Associated Genes Found** |
| Receptor and Signaling Pathways | 2 | GFRA3, FLT1 |
| Epigenetic Regulation | 1 | COPRS |
| Structural Role | 1 | NEFM |
| Cell Cycle Regulation | 1 | STIL |
| 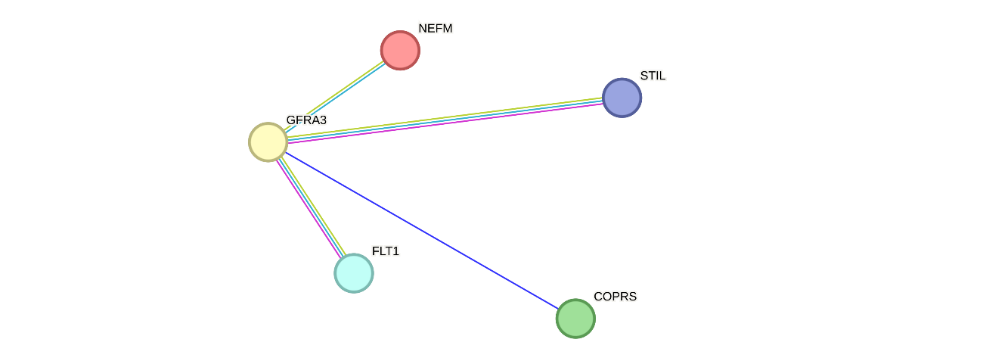 | | |

| **POSTN (N1)** | | |
| --- | --- | --- |
| **Term** | **Nr. Genes** | **Associated Genes Found** |
| ECM – Receptor interaction | 4 | [COL1A1, ITGA2, THBS1, THBS4] |
| Malaria | 3 | [IL6, THBS1, THBS4] |
| **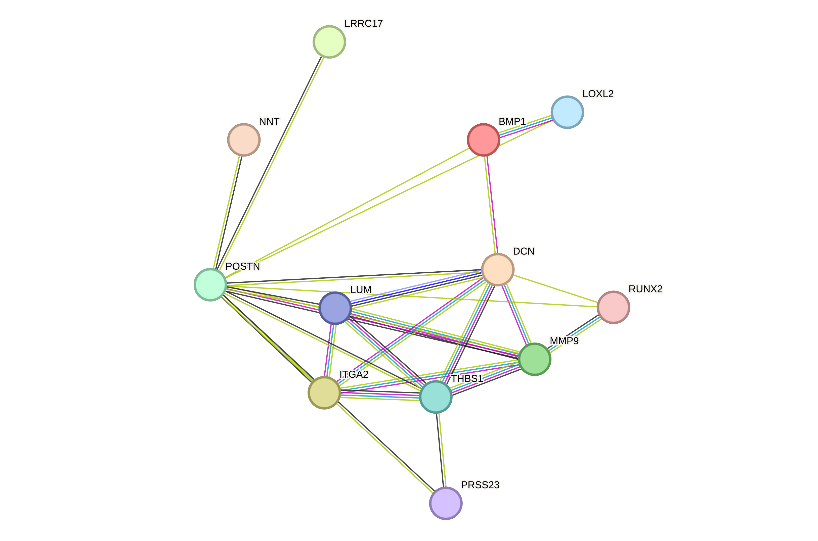** | | |

| **POSTN (N2)** | | |
| --- | --- | --- |
| **Term** | **Nr. Genes** | **Associated Genes Found** |
| Extracellular Matrix and Structural Roles | 5 | POSTN, BGN, DCN, LOXL2, LUM |
| Bone Formation and Remodeling | 3 | BMP1, RUNX2, LRRC17 |
| Cell Adhesion and Integrins | 1 | ITGA2 |
| Proteolysis and ECM degradation | 2 | MMP9, PRSS23 |
| Metabolism and Redox Regulation | 1 | NNT |
| 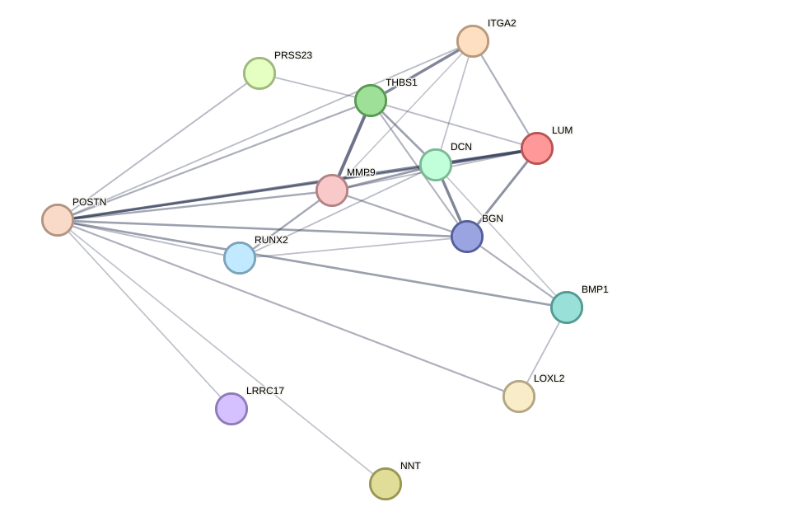 | | |

| **S1PR1 (N1)** |
| --- |
| **KEGG** |
| **FoxO signaling pathway** |
| **Chemokine signaling pathway** |
| **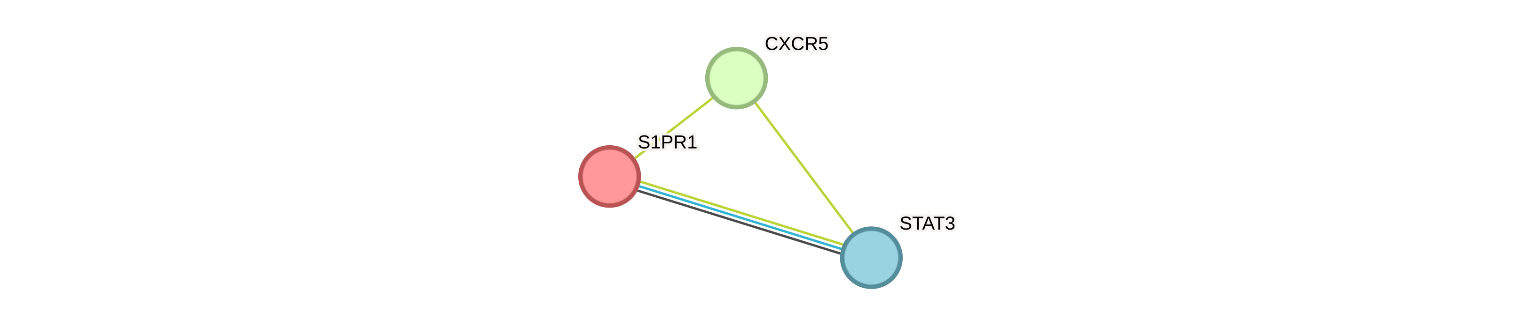** |

| **S1PR1 (N1)** |
| --- |
| **KEGG** |
| **Chemokine signaling pathway** |
| **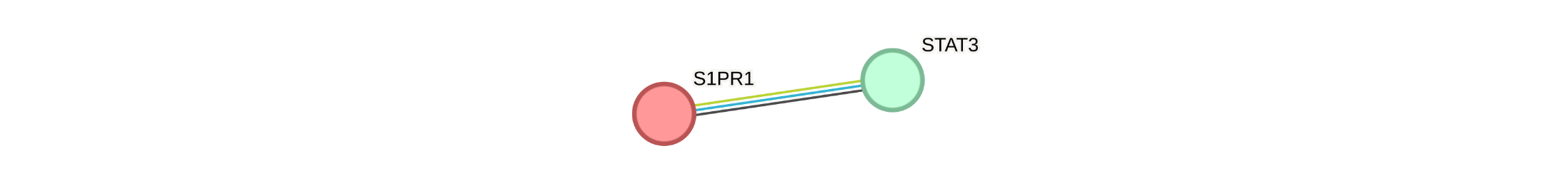** |

| **STC1 (N1)** |
| --- |
| **KEGG** |
| **cAMP signaling pathway** |
| **Insulin secretion** |
| **Neuroactive ligand-receptor interaction** |
| **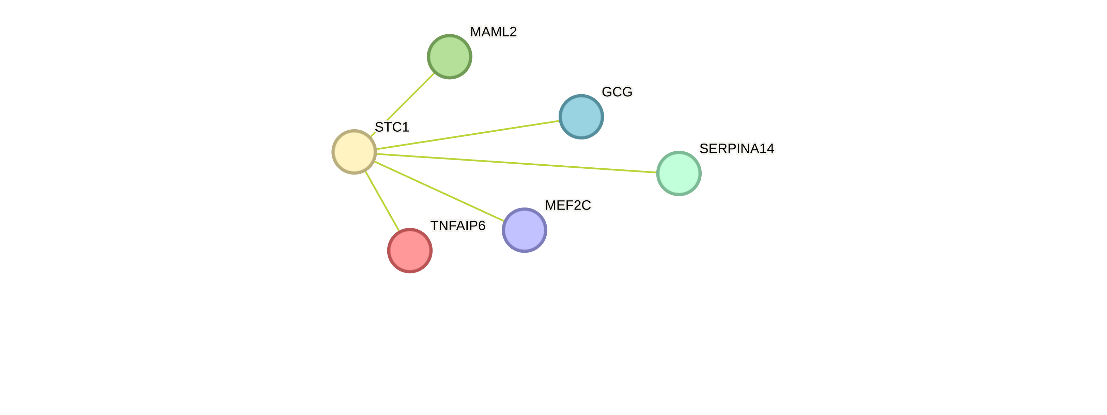** |

| **STC1 (N2)** |  |  |
| --- | --- | --- |
| **Term** | **Nr. Genes** | **Associated Genes Found** |
| Metabolism and Homeostasis | 1 | STC1 |
| Glucose metabolism and hormones | 1 | GCG |
| Transcription regulation | 1 | MAML2 |
| Protease inhibition | 1 | SERPIN14 |
| 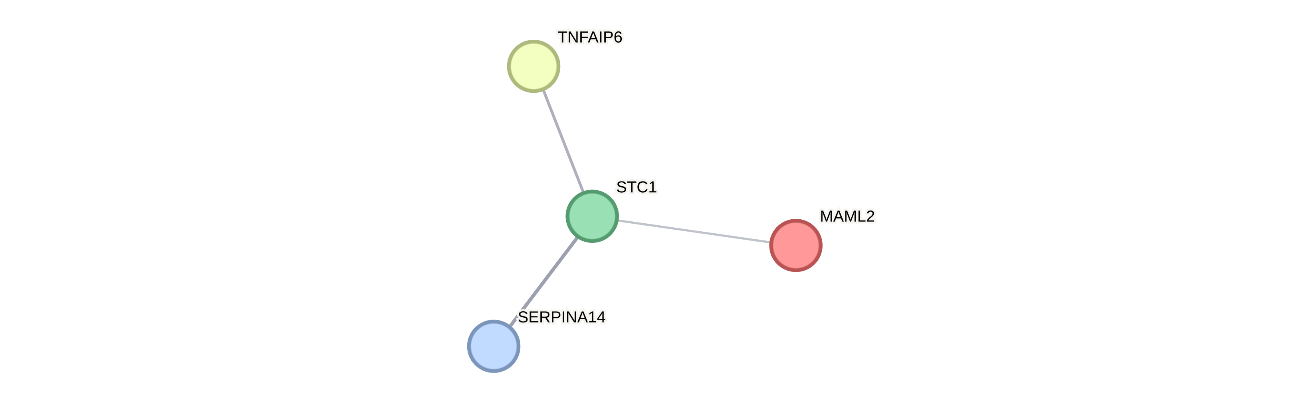 | | |

| **EFEMP1 (N1)** |
| --- |
| **KEGG** |
| **N/A** |

| **EFEMP1 (N2)** |
| --- |
| **KEGG** |
| **N/A** |

| **KRT8 (N1)** |
| --- |
| **KEGG** |
| **Fanconi anemia pathway** |
| **Protein processing in endoplasmic reticulum** |
| **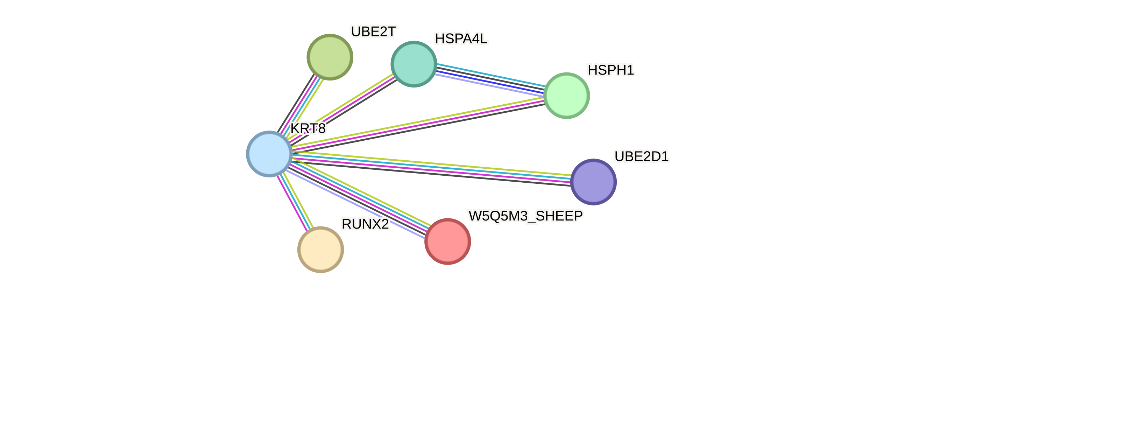** |

| **KRT8 (N2)** |
| --- |
| **KEGG** |
| **Cell cycle** |
| **Ubiquitin mediated proteolysis** |
| **Human T-cell leukemia virus 1 infection** |
| **Progesterone-mediated oocyte maturation** |
| **Oocyte meiosis** |
| **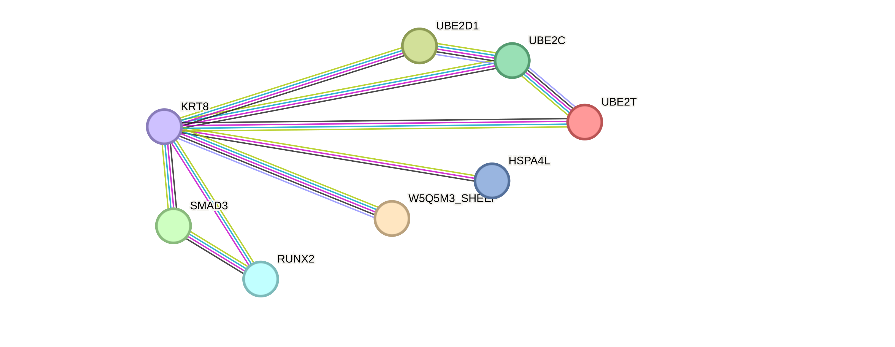** |

| **MMP1 (N1)** |
| --- |
| **KEGG** |
| **Proteoglycans in cancer** |
| Relaxin signaling pathway |
| AGE-RAGE signaling pathway in diabetic complications |
| Focal adhesion |
| Hypertrophic cardiomyopathy |
| IL-17 signaling pathway |
| Rheumatoid arthritis |
| Hematopoietic cell lineage |
| HIF-1 signaling pathway |
| Platelet activation |
| ECM-receptor interaction |
| Cellular senescence |
| PI3K-Akt signaling pathway |
| Chagas disease |
| Pathways to cancer |
| Amoebiasis |
| 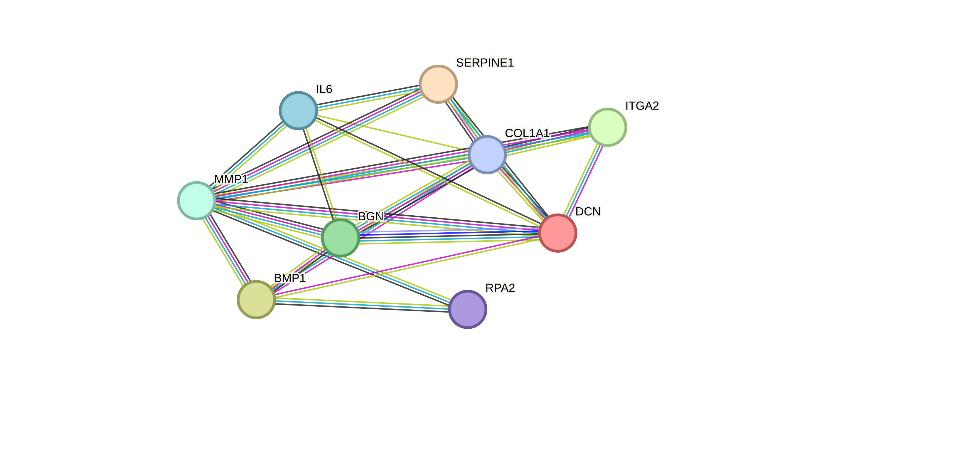 |

| **MMP1 (N2)** |
| --- |
| **KEGG** |
| **Proteoglycans in cancer** |
| **Bladder cancer** |
| **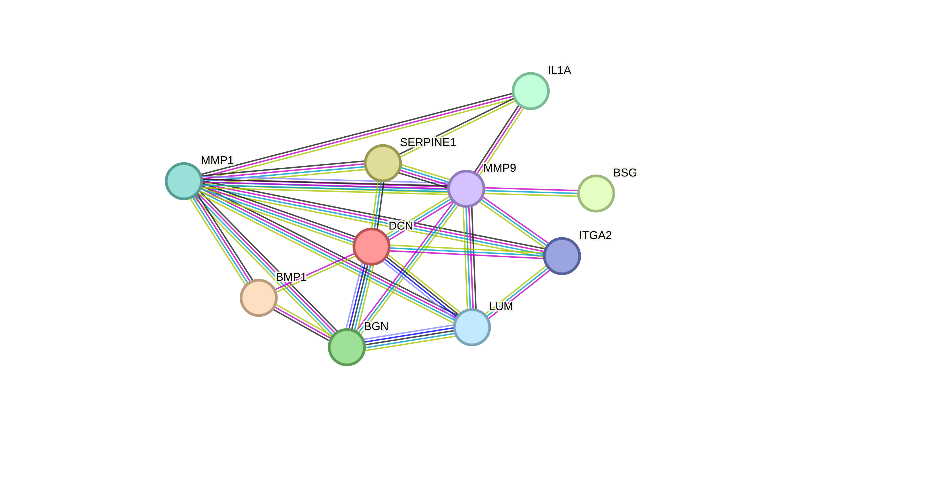** |

| **MME (N1)** |
| --- |
| **KEGG** |
| **Renin-angiotensin system** |
| **Renin secretion** |
| **Protein digestion and absorption** |
| **Hypertrophic cardiomyopathy** |
| **Chagas disease** |
| **Alzheimer disease** |
| **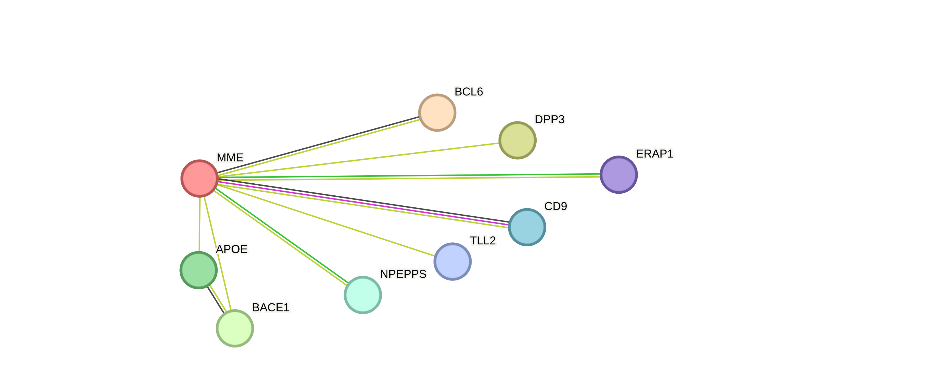** |

| **MME (N2)** |
| --- |
| **KEGG** |
| **Renin-angiotensin system** |
| **Renin secretion** |
| **Protein digestion and absorption** |
| **Hypertrophic cardiomyopathy** |
| **Neuroactive ligand-receptor interaction** |
| **Adrenergic signaling in cardiomyocytes** |
| **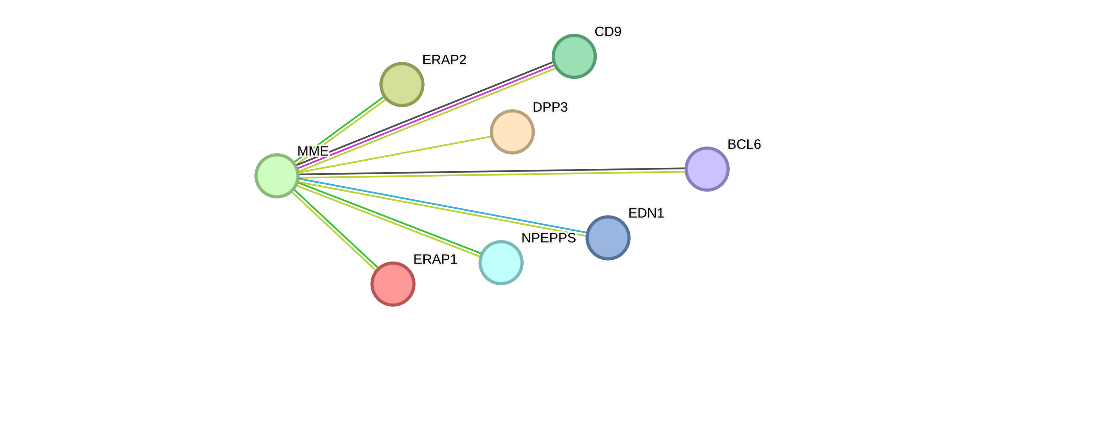** |

| **CYP19 (N1)** |
| --- |
| **KEGG** |
| **Ovarian steroidogenesis** |
| **Steroid hormone biosynthesis** |
| **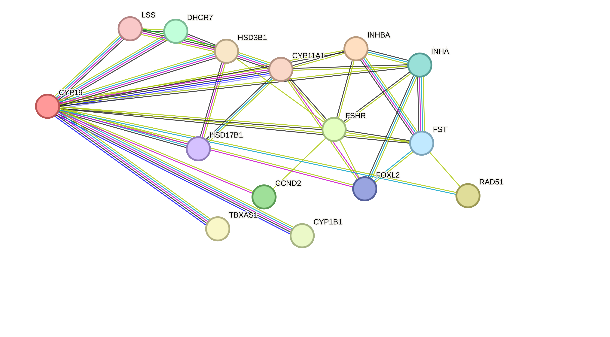** |

| **CYP19 (N2)** | | |
| --- | --- | --- |
| **Term** | **Nr. Genes** | **Associated Genes Found** |
| Steroid hormone biosynthesis | 4 | [CYP11A1, CYP19, HSD17B1, HSD3B1] |
| Ovarian steroidogenesis | 6 | [CYP11A1, CYP19, FSHR, HSD17B1, HSD3B1, PRKACA] |
| Cortisol synthesis and secretion | 3 | [CYP11A1, HSD3B1, PRKACA] |
| 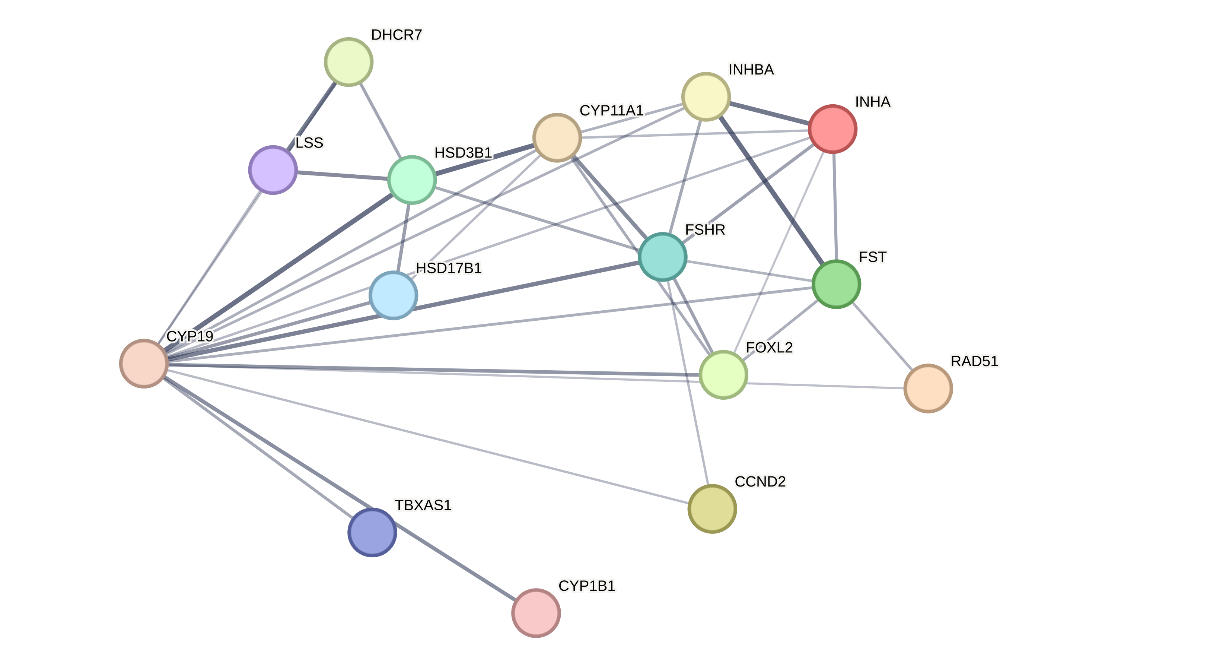 | | |

| **VNN2 (N1)** |
| --- |
| **KEGG** |
| **Pantothenate and CoA biosynthesis** |
| **Valine, leucine and isoleucine degradation** |
| **Cysteine and methionine metabolism** |
| **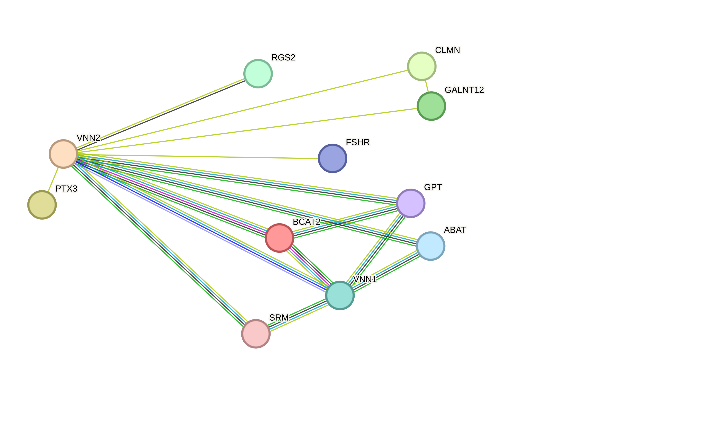** |

| **VNN2 (N2)** |
| --- |
| **KEGG** |
| **Pantothenate and CoA biosynthesis** |
| **Cysteine and methionine metabolism** |
| **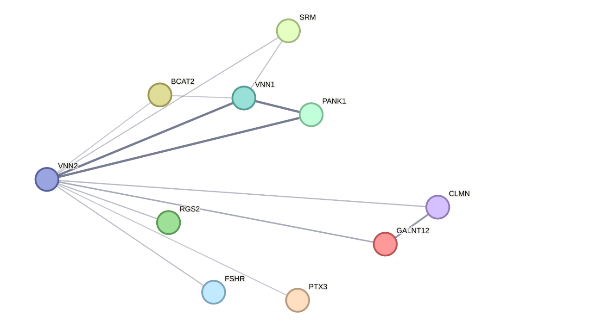** |

| **RHCG (N1)** |
| --- |
| **KEGG** |
| **Collecting duct acid secretion** |
| **Rheumatoid arthritis** |
| **Synaptic vesicle cycle** |
| **Oxidative phosphorylation** |
| **Phagosome** |
| **Human papillomavirus infection** |
| **Lysosome** |
| **Tuberculosis** |
| **Metabolic pathways** |
| **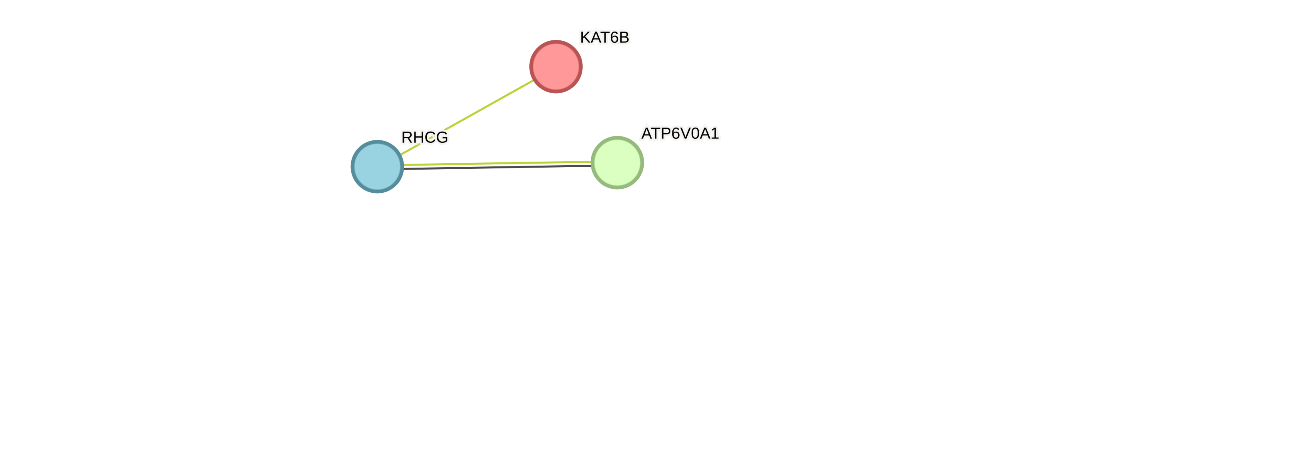** |

| **RHCG (N2)** |  |  |
| --- | --- | --- |
| **Term** | **Nr. Genes** | **Associated Genes Found** |
| Ion transport and pH regulation | 2 | RHCG, ATP6V0A1 |
| Epigenetic Regulation and Transcription | 1 | KAT6B |
| Bone Remodeling and Immune Modulation | 1 | LRRC17 |
| 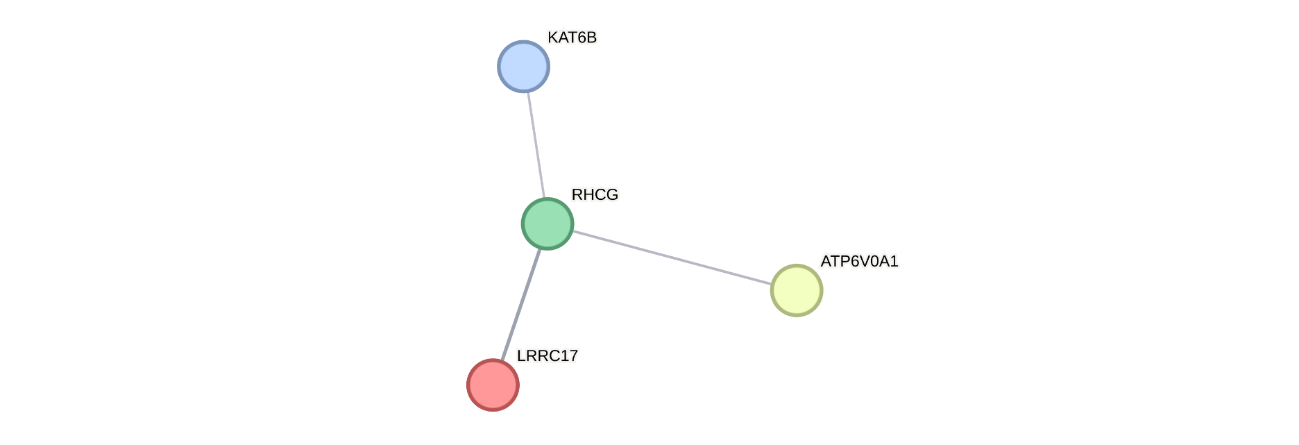 | | |

| **NDNF (N1)** |
| --- |
| **KEGG** |
| **Fatty acid metabolism** |
| **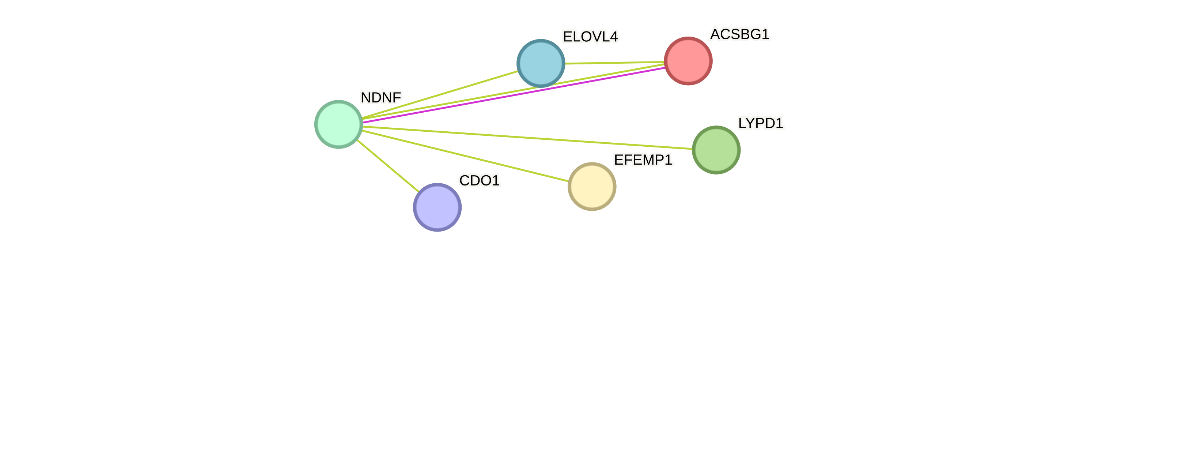** |

| **NDNF (N2)** |
| --- |
| **KEGG** |
| **Fatty acid metabolism** |
| **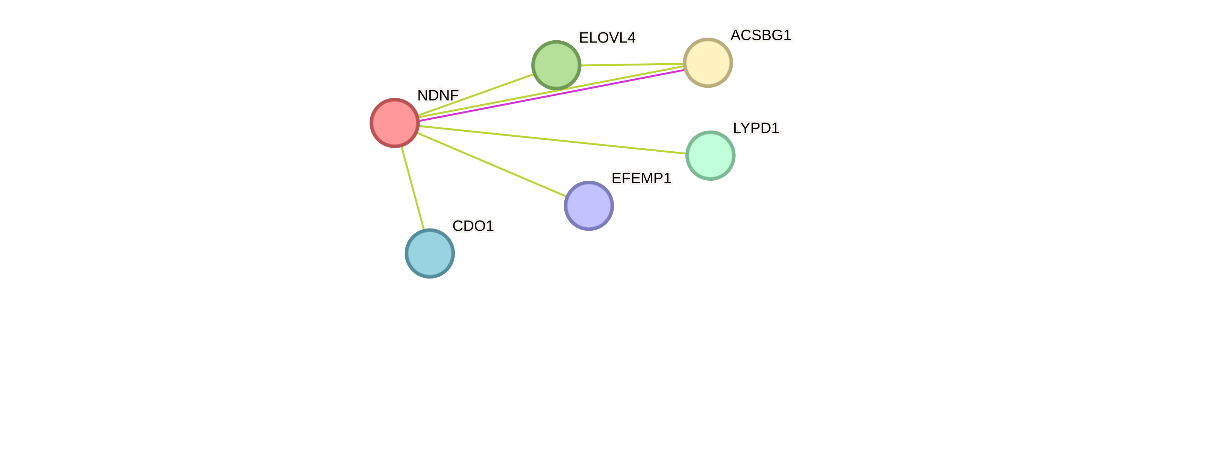** |

| **TNFAIP6 (N1)** | | |
| --- | --- | --- |
| **Term** | **Nr. Genes** | **Associated Genes Found** |
| Inflammatory Response and cytokines | 4 | TNFAIP6, CXCL10, CXCL9, IL6 |
| Growth factors and tissue development | 2 | EREG, INHBA |
| Extracellular Matrix and structural roles | 3 | ITIH5, PTX3, THBS1 |
| Stem Cells and Angiogenesis | 1 | CD34 |
| Metabolism and Calcium regulation | 1 | STC1 |
| 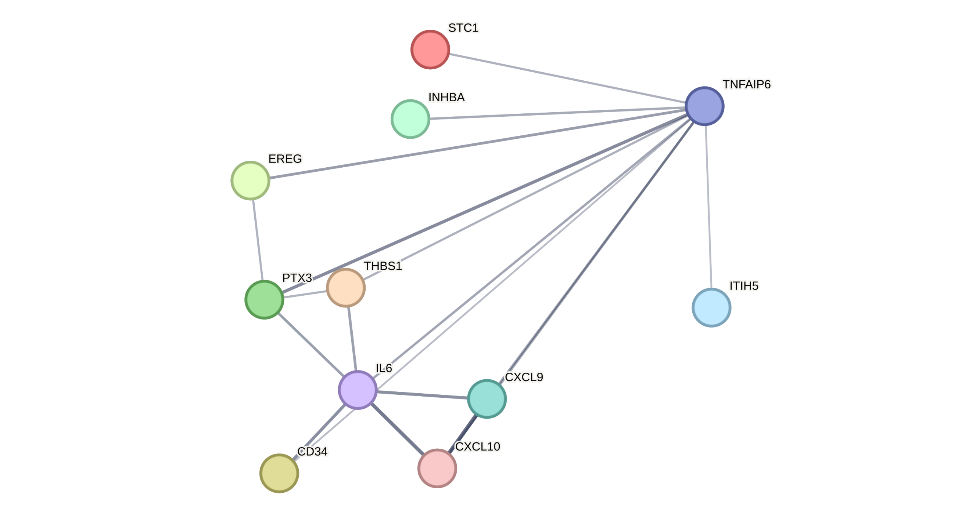 | | |

| **TNFAIP6 (N2)** |
| --- |
| **KEGG** |
| **Viral protein interaction with cytokine and cytokine receptor** |
| **Toll-like receptor signaling pathway** |
| **Cytokine – cytokine receptor interaction** |
| **Malaria** |
| **Cytosolic DNA-sensing pathway** |
| **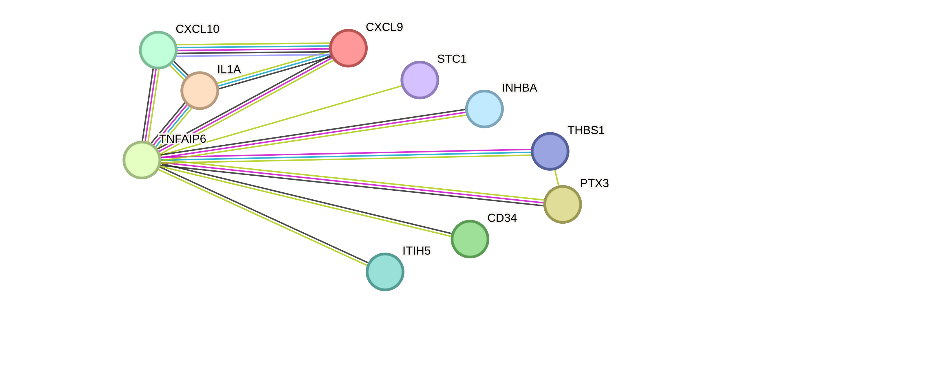** |

| **TMEM37 (N1)** |
| --- |
| **KEGG** |
| **N/A** |

| **TMEM37 (N2)** |
| --- |
| **KEGG** |
| **N/A** |
|  |
