# Supplementary material for: Tailor-made 3D in vitro maturation of early antral follicles uncovers cumulus-cell transcriptomic driver signature to predict oocyte competence
Source: Front Endocrinol (Lausanne). 2025 Oct 1;16:1629815. doi: 10.3389/fendo.2025.1629815 (PMC12520894; doi:10.3389/fendo.2025.1629815)
Supplement: Supplementary Table 1 — (Excel). The 12 centrality coefficients of each DEG of Network 1(MIIEndpoint- GVStartpoint) (Sheet: N1 MII-GV) and Network 2(GVEndpoint-GVStartpoint) (Sheet: N2 GV-GV) were scored using CytoHUBba. More in detail, they are closeness, degree, MCC, radiality, stress, MCN, DNMC, betweenness, clustering coefficient, eccentricity, bottleneck, and EPC. Network 1(MIIEndpoint- GVStartpoint) and Network 2(GVEndpoint-GVStartpoint) top 10 DEGs defined on each centrality coefficient score (Sheets: Top 10 N1 and N2 respectively). Venn diagram analysis of the top 10 DEGs of Network 1(MIIEndpoint- GVStartpoint) (Sheet: Ranking N1) and Network 2(GVEndpoint-GVStartpoint)(Sheet: Ranking N2) shows DEGs overlapping across the 12 algorithms. DEGs that are in the top 10 in at least 5 of the 6 algorithms are highlighted in bold. (Network1_Normalized) and (Network2_Normalized) include dataset values that have been statistically normalized using the standard score formula. [file DataSheet1.zip › Supplementary datasheets and tables/Supplementary Datasheet 9.docx]

**Supplementary Datasheet 9. From Microarray to Bench: qPCR Validation of Selected Drivers: Real-Time qPCR validation of selected drivers of each pairwise.**


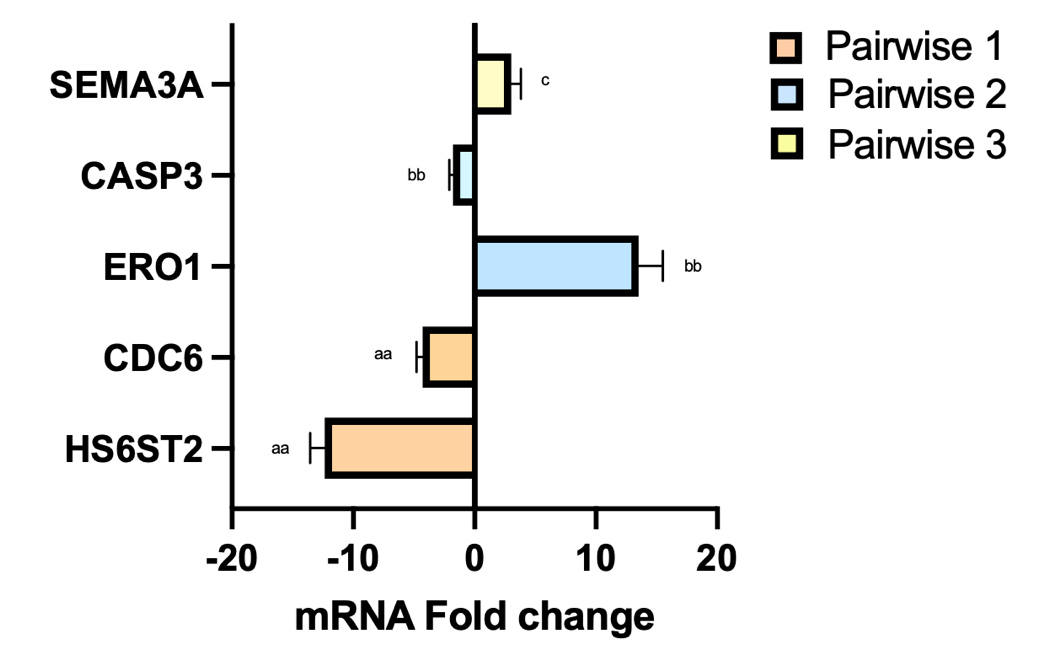


**From Microarray to Bench: qPCR Validation of Selected Drivers: Real-Time qPCR validation of selected drivers of each pairwise.** Fold-changes are related to the mean ± SD of 2^- ∆∆Ct values obtained in n = 3 independent experiments. For pairwise 1, the significance of *HS6ST2* and *CDC6* data related to CCs enclosing MII oocytes at the end of FEO (p < 0.01) was statistically analyzed vs. CCs enclosing GV oocytes as the starting point of FEO and indicated with aa. For pairwise 2, the significance of *ERO1* and *CASP3* data related to CCs enclosing GV oocytes at the end of FEO (p < 0.01) was statistically analyzed vs. CCs enclosing GV oocytes as the starting point of FEO and indicated with bb. For pairwise 3, the significance of *SEMA3A* data related to CCs enclosing MII oocytes at the end of FEO (p < 0.05) was statistically analyzed vs. CCs enclosing GV oocytes at the end of FEO and indicated with (c).
