# Supplementary material for: Tailor-made 3D in vitro maturation of early antral follicles uncovers cumulus-cell transcriptomic driver signature to predict oocyte competence
Source: Front Endocrinol (Lausanne). 2025 Oct 1;16:1629815. doi: 10.3389/fendo.2025.1629815 (PMC12520894; doi:10.3389/fendo.2025.1629815)
Supplement: Supplementary Table 1 — (Excel). The 12 centrality coefficients of each DEG of Network 1(MIIEndpoint- GVStartpoint) (Sheet: N1 MII-GV) and Network 2(GVEndpoint-GVStartpoint) (Sheet: N2 GV-GV) were scored using CytoHUBba. More in detail, they are closeness, degree, MCC, radiality, stress, MCN, DNMC, betweenness, clustering coefficient, eccentricity, bottleneck, and EPC. Network 1(MIIEndpoint- GVStartpoint) and Network 2(GVEndpoint-GVStartpoint) top 10 DEGs defined on each centrality coefficient score (Sheets: Top 10 N1 and N2 respectively). Venn diagram analysis of the top 10 DEGs of Network 1(MIIEndpoint- GVStartpoint) (Sheet: Ranking N1) and Network 2(GVEndpoint-GVStartpoint)(Sheet: Ranking N2) shows DEGs overlapping across the 12 algorithms. DEGs that are in the top 10 in at least 5 of the 6 algorithms are highlighted in bold. (Network1_Normalized) and (Network2_Normalized) include dataset values that have been statistically normalized using the standard score formula. [file DataSheet1.zip › Supplementary datasheets and tables/Supplementary Datasheet 8.docx]

**Supplementary Datasheet 8.**

1. **Literature annotations on female reproductive role of the identified candidate driver genes (Network 1 and 2).**

| **DRIVER GENE NAME** | **NETWORK** | **MCODE** | **DEG**  **Fold change** | **P-value** | **FEMALE FERTILITY EFFECTS** | |
| --- | --- | --- | --- | --- | --- | --- |
|  |  |  |  |  | **KNOCKOUT/MUTANT MICE** | **EFFECTS RELATED TO MATURATION** |
| ***EFHD1**** | 1 | N/A | -10.36 | 0.00000707 | N/A | N/A in CCs. |
| ***HS6ST2*** |  | N/A | -11.37 | 0.000000204 | N/A | In vitro maturation of cumulus oocyte complexes in the presence of exogenous heparin, which antagonizes *HSPG* signaling, prevented cumulus expansion and blocked the induction of cumulus-specific matrix genes: *HAS2* and *TNFAIP6. HS6ST2* is an enzyme that is necessary to sulfate proteoglycans for cumulus expansion (1). |
| ***SLC35G1*** |  | N/A | -10.08 | 0.000016 | N/A | Loss of *SLC35G1* expression in Granulosa cells, is consistent with a switch form GC transcriptome to a transcriptome of a large luteal cell (2). |
| ***HBA1**** | 2 | N/A | 39.04 | 0.0000000163 | N/A | N/A in CCs. |
| ***SLC39A8*** |  | N/A | 15.33 | 2.04E-08 | N/A | N/A in CCs. |
| ***ERO1A*** |  | N/A | 14.47 | 2.35E-08 | N/A | Possible anti-apoptotic role and regulate steroidogenesis in granulosa cells in mice (3). |
| ***TKDP5**** |  | N/A | 16.12 | 1.21E-05 | N/A | N/A in CCs.  Trophoblast Kunitz domain proteins can interact with components of the extracellular matrix, which is essential for cell adhesion and migration during early development. By stabilizing the extracellular matrix around the oocyte, they help ensure proper cell signaling and nutrient transport (4) |
| ***CALCRL**** |  | N/A | 9.77 | 0.000000797 | N/A | N/A in CCs. |
| ***ELOVL6*** |  | 9 | -10.50 | 0.000000451 | Mutation causes decreased fatty acid oxidation and decreased circulating steroid levels that are important in hormonal steroid biosynthesis  (J:130031 MGI database) | Various proteins that belong to the *ELOVL* family have been reported through different stages of Ewe and Bovine folliculogenesis (5). |
| *S1PR1* | 1,2 | N/A | 10.88; 9.83 | 7,45E-05; 0.00000775 | N/A | N/A in CCs.  *S1P* intervenes in cell growth, proliferation, differentiation, cell survival, migration and angiogenesis regulation. Also protects germ cells from cell death in vivo and in vitro (6). |
| *STC1* | 1,2 | N/A | 17.94; 20.95 | 0,0001; 0.00000105 | N/A | *STC1* in chicken folliculogenesis study inhibited proliferation and stimulated the apoptosis of follicular granulosa cells, as well as decreasing P4 and E2 (7). |
| *EFEMP1* | 1,2 | N/A | 11.3; 10.48 | 1,88E-06; 0.0000016 | Efemp1tm2Lmar/Efemp1tm2Lmar knockout causes reduced infertility in mice, as well as abnormal reproductive morphology, specifically atrophy and degeneration in young individuals (J: 132043 MGI database) | N/A in CCs.  *EFEMP1* is present in ovarian cancer cells through growth, invasion and metastasis. KO of this gene results in inhibition of cancer progression and induces cell cycle arrest (8). |
| *KRT8* | 1,2 | N/A | 14.1; 14.9 | 6,61E-07; 0.000000205 | Krt8tm1Rgo/Krt8tm1Rgo mutation causes reduced female fertility, abnormal embryo quality (degeneration of trophoblastic cells) (J:12899 and 121811 MGI database) | N/A in CCs.  *KRT-8* positive cells participate in ovarian follicle-like structure formation in a xenograft mice model. As it plays an essential role in primordial germ cell migration and involved in cytoskeletal organization, cell migration, adhesion and metastasis (9). |
| *POSTN* | 1,2 | 9 | 77.4; 51.53 | 4,22E-07; 5.03E-08 | N/A | *POSTN* affects granulosa cells proliferation and follicle development, potentially improving fecundity in sheep (10). |
| *MMP1* | 1,2 | N/A | 13.47; 12.47 | 7,35E-06; 0.000000062 | Mutation in humans causes multiple oocyte maturation defects, including oocyte arrest at metaphase I (https://www.alliancegenome.org/gene/HGNC:7155) | *MMP1* expression has been found in granulosa cells of all follicle stages. High mRNA and protein expression of *MMP1* indicate its involvement in antrum formation and expansion (11). |
| *MME* | 1,2 | N/A | 10.28; 11.45 | 2,65E-06; 0.000000263 | N/A | N/A in CCs. |
| *VNN2* | 1,2 | N/A | 12.24; 13.11 | 0.0006;0.0003 | N/A | N/A in CCs.  *VNN2* mRNA levels can rise dramatically in ovulatory follicles compared to dominant follicles​ (12). |
| *RHCG* | 1,2 | N/A | 11.77; 13.84 | 0.001; 0.0000441 | N/A | N/A in CCs. |
| *NDNF* | 1,2 | N/A | 11.44; 11.11 | 1,37E-06; 0.000000128 | In humans, this mutation affects the reproductive system as ovarian hypoplasia characterized by fibrous stroma without generative elements or small number of primary follicles, low estrogen levels (https://hpo.jax.org/browse/term/HP:0000118) | *NDNF* is involved in endothelial cell migration and angiogenesis. Changes in expression level of *NDNF* in the follicle may be important for a concurrent regulation in the vasculature following follicular selection (13). |
| *TNFAIP6* | 1,2 | N/A | 37.89; 18.59 | 6,31E-06; 1.36E-08 | Tnfaip6^tm1Cful^/Tnfaip6^tm1Cful^  This mutation in mice causes an absence in cumulus expansion, follicles fail to display normal cumulus mucification after ovulation induction. Also, female homozygotes are sterile due to impaired cumulus matrix formation. (J:82539 MGI database) | The downregulation of *TNFAIP6* inhibits in vitro maturation in ovine oocytes and proliferation of cumulus cells (14). |
| *TMEM37* | 1,2 | N/A | 10.16; 10.41 | 0.0001; 0.00000012 | N/A | N/A in CCs. |
| *CYP19* | 1,2 | 9 | -12.31; -10.93 | 2,85E-07; 5.54E-08 | N/A | *CYP19* gene influences ovarian response and is actively involved in aromatase expression in granulosa cells (15,16). |
| *GFRA3* | 1,2 | N/A | -17.35; -13.21 | 2,14E-07; 0.000000166 | N/A | N/A in CCs. |
| *INHA* | 1,2 | 9 | -14.83; -17.69 | 1,61E-07; 1.99E-08 | [Inha^em1Crah^](https://www.informatics.jax.org/allele/MGI:7491749)/[Inha^em1Crah^](https://www.informatics.jax.org/allele/MGI:7491749) Mutant mice have 33% abnormal oocyte after ovulation, also an increased primary ovarian follicle number (J:324311 by MGI database)  Abnormal ovarian follicle morphology (J:3315 by MGI database) | A mutation of *INHA* can be the main cause of premature ovarian failure (POF) and other ovarian functional abnormalities; *INHA* and *INHB* are members of *TGFB* superfamily, and it is suggested that they regulate important stages of oocyte maturation *in vivo* and *in vitro (17).* |
| ***KIF11*** | 1 | 1 | -2.96 | 0.0001 | [Kif11^tm1Much^](https://www.informatics.jax.org/allele/MGI:3803280)/[Kif11^tm1Much^](https://www.informatics.jax.org/allele/MGI:3803280)  This mutation causes direct effects on embryo morphology, functionality and death in mutant mice embryos. (J:137799 from MGI database.) | N/A in CCs.  IVM oocytes present a higher expression of *KIF11* on meiosis II spindle than IVV oocytes, which leads to sensitivity to the inhibition of *KIF11 (18).*  Inhibition of *KIF11* activity disturbs the cell cycle progression (19). |
| ***CDC6*** | 1 | 1 | -4.74 | 0.0000115 | N/A | N/A in CCs.  *CDC6* was detected in proliferating follicular cells of primary and secondary follicles, but not in primary oocytes. The overexpression of *CDC6* in primary cells has been shown to promote DNA hyper replication and can lead to senescence (20,21). |
| ***CASP3*** | 2 | 7 | -2.03 | 0.0001 | [Casp3^tm1Flv^](https://www.informatics.jax.org/allele/MGI:2158753)/[Casp3^tm1Flv^](https://www.informatics.jax.org/allele/MGI:2158753)  Mutation causes the blockage of apoptosis in granulosa cells of atretic follicles, as well as shrunken and disorganized atretic follicles (J:70205 by MGI database). | *CASP3* is present during apoptosis in Granulosa Cells and its action is activated when the follicles begin to leave the resting pool. The same study concludes the presence of *CASP3* in granulosa cells of atretic, but not healthy antral follicles (22). |
| *CDCA8* | 1, 2 | 2, 1 | -2.31 | 0.0000496 | N/A | N/A in CCs.  Study demonstrates that *CDCA8* expression is elevated, and its protein exhibits dynamic localization in human oocytes from germinal vesicle breakdown (GVBD) to metaphase II (MII) ((23). |
| *CCNA2* | 1, 2 | 2, 2 | -3.74 | 0.00000876 | N/A | N/A in CCs. |
| *PLK1* | 1, 2 | 1, 1 | -2.37 | 0,0003 | N/A | N/A in CCs. |

**B. Literature annotations on female reproductive role of the identified candidate driver genes (Pairwise 3).**

| **DRIVER GENE NAME** | **NETWORK** | **MCODE** | **DEG**  **Fold change** | **P-value** | **FEMALE FERTILITY EFFECTS** | |
| --- | --- | --- | --- | --- | --- | --- |
|  |  |  |  |  | **KNOCKOUT/MUTANT MICE** | **EFFECTS RELATED TO MATURATION** |
| *IL1A* | 3 | N/A | -3.27 | 0.0000176 | N/A | It has been shown that *IL1A* deficiency increases the expression of follicle stimulating hormone receptors in granulosa cells. Also, elevated level of *IL-1* in follicle fluids may be the major causal factor for follicular apoptosis that consequently impairs follicle reserve and ovarian functions by follicle depletion (24,25). |
| *NXPH4** |  | N/A | -2.34 | 0.0009 | N/A | N/A in CCs. |
| *HSPA1A* |  | N/A | -2.2 | 0.0018 | N/A | *HSPA1A* encodes for *HSP70* protein, its expression has been found in cumulus cells, oocytes and embryos under non – heat stress conditions. It may play a role in fertilization and embryo development, acting against apoptosis. Upregulated *HSPA1A* has been found in poor quality CCs (26). |
| *LOXL2** |  | N/A | -2.24 | 0.0025 | N/A | N/A in CCs  Expression of *LOXL2* is present in multiple cancers and is an indicator of tumor progression. Mainly involved as an extracellular enzyme that participates in maturation and remodeling of the ECM. Suppression of *LOXL2* leads to an inhibition of cell migration and invasion in trophoblastic cells. Downregulation of *LOXL2* has been described in ovarian tumor progression (27,28). |
| *VNN1* |  | N/A | -2.02 | 0.0083 | N/A | *VNN1* could be triggered after the decline of oocyte competence and initiation of follicular atresia, due to oxidative-stress stimulus. This molecule possibly has a role in the protection of the global follicle oxidative state (29) |
| *DDIT4* |  | N/A | -3.54 | 0.0104 | [Ddit4tm1.1Sinc](https://www.informatics.jax.org/allele/MGI:5432482)/[Ddit4tm1.1Sinc](https://www.informatics.jax.org/allele/MGI:5432482)  In wildtype mice, they exhibit normal serum levels of luteinizing hormone, follicle stimulating hormone and testosterone levels.  ([J:186462](https://www.informatics.jax.org/reference/J:186462) from MGI  database) | May play a crucial role in the development and progression of cells through oocyte meiosis (30). |
| *VLDLR* |  | N/A | -2.17 | 0.0106 | [Vldlrem1(IMPC)Mbp](https://www.informatics.jax.org/allele/MGI:6277005)/[Vldlrem1(IMPC)Mbp](https://www.informatics.jax.org/allele/MGI:6277005)  In mutant mice, the knockout of this gene causes a blind uterus phenotype. ( [J:211773](https://www.informatics.jax.org/reference/J:211773) from MGI database) | *VLDLR*, together with other genes involved in the cholesterol biosynthesis pathway, are down regulated in IVM CCs in comparison with in vivo matured. Suggesting IVM alters CCs ability to provide lipids for oocyte nuclear maturation (31). |
| *CDA* |  | N/A | -2.21 | 0.024 | N/A | Activation-induced cytidine deaminase is a possible regulator of crosstalk between oocytes and granulosa cells through *GDF-9* and SCF feedback system (32). |
| *SEMA3A** |  | N/A | 2.01 | 0.0288 | N/A | N/A in CCs.  *SEMA3* has been demonstrated to have a role in neuronal control of puberty and linked to reproductive disorders in humans. Indeed, mutations have been identified in patients with Kallmann Syndrome (KS) (33,34) resulting in  pubertal failure. Furthermore, *SEMA3* has been linked to female reproductive failure due to an autosomal dominant mutation that causes hypogonadotropic hypogonadism (HH) (35).  Its expression is regulated by gonadotropins in ovarian cancer, where it plays a functional role in controlling angiogenesis (35). Consistently, it has been found increased in the Plasma of Women with Diminished Ovarian Reserve Who Respond Better to Controlled Ovarian Stimulation (36).  Other members of the *SEMA* family have been described as linked to cell-to-cell communication, particularly in the context of oocyte cumulus complex maturation and follicular development (37–39) . |
| *ADIRF** |  | N/A | -2.2 | 0.001 | N/A | N/A in CCs. |
| *ERO1L** |  | N/A | -2.28 | 0.0251 | N/A | N/A in CCs.  A previous study demonstrated that the overexpression of this gene inhibits apoptosis of granulosa cells, and the knockdown promoted the opposite action (3). |

**References**

1. Watson LN, Mottershead DG, Dunning KR, Robker RL, Gilchrist RB, Russell DL. Heparan Sulfate Proteoglycans Regulate Responses to Oocyte Paracrine Signals in Ovarian Follicle Morphogenesis. Endocrinology [Internet]. 2012 Sep 1 [cited 2025 Apr 21];153(9):4544–55. Available from: https://dx.doi.org/10.1210/en.2012-1181

2. Romereim SM, Summers AF, Pohlmeier WE, Zhang P, Hou X, Talbott HA, et al. Gene expression profiling of bovine ovarian follicular and luteal cells provides insight into cellular identities and functions. Mol Cell Endocrinol [Internet]. 2017 Jan 5 [cited 2025 Apr 21];439:379–94. Available from: https://pubmed.ncbi.nlm.nih.gov/27693538/

3. Hu J, Jin J, Qu Y, Liu W, Ma Z, Zhang J, et al. ERO1α inhibits cell apoptosis and regulates steroidogenesis in mouse granulosa cells. Mol Cell Endocrinol [Internet]. 2020 Jul 1 [cited 2025 Apr 21];511. Available from: https://pubmed.ncbi.nlm.nih.gov/32376276/

4. Smitz J, Platteau P. Influence of human chorionic gonadotrophin during ovarian stimulation: An overview. Reproductive Biology and Endocrinology [Internet]. 2020 Aug 6 [cited 2025 Apr 21];18(1):1–17. Available from: https://rbej.biomedcentral.com/articles/10.1186/s12958-020-00639-3

5. Dalbies-Tran R, Cadoret V, Desmarchais A, Elis S, Maillard V, Monget P, et al. A Comparative Analysis of Oocyte Development in Mammals. Cells [Internet]. 2020 Apr 1 [cited 2025 Apr 21];9(4). Available from: https://pubmed.ncbi.nlm.nih.gov/32316494/

6. Guo L, Ou X, Li H, Han Z. Roles of sphingosine-1-phosphate in reproduction. Reprod Sci [Internet]. 2014 [cited 2025 Apr 21];21(5):550–4. Available from: https://pubmed.ncbi.nlm.nih.gov/24336672/

7. Sun J, Zhang P, Wang D, Zhu S, Ma X, Du Z, et al. Integrative analyses of the mRNA expression profile reveal the involvement of STC1 in chicken folliculogenesis. J Anim Sci [Internet]. 2023 Jan 3 [cited 2025 Apr 21];101. Available from: https://dx.doi.org/10.1093/jas/skad295

8. Yin X, Fang S, Wang M, Wang Q, Fang R, Chen J, et al. EFEMP1 promotes ovarian cancer cell growth, invasion and metastasis via activated the AKT pathway. Oncotarget [Internet]. 2016 Jun 25 [cited 2025 Apr 21];7(30):47938–53. Available from: https://www.oncotarget.com/article/10296/text/

9. Yu DCW, Wu FC, Wu CE, Chow LP, Ho HN, Chen HF. Human pluripotent stem cell-derived DDX4 and KRT-8 positive cells participate in ovarian follicle-like structure formation. iScience [Internet]. 2020 Jan 22 [cited 2025 Apr 21];24(1). Available from: https://pubmed.ncbi.nlm.nih.gov/33490911/

10. Abudureyimu G, Wu Y, Wang L, Hao G, Chen Y, Yu J, et al. POSTN promotes granulosa cell proliferation in sheep follicles through focal adhesion. Gene Rep. 2024 Jun 1;35:101890.

11. Fujihara M, Yamamizu K, Wildt DE, Songsasen N. Expression pattern of matrix metalloproteinases changes during folliculogenesis in the cat ovary. Reproduction in Domestic Animals [Internet]. 2016 Oct 1 [cited 2025 Apr 21];51(5):717–25. Available from: https://onlinelibrary.wiley.com/doi/full/10.1111/rda.12736

12. Lussier JG, Sayasith K, Sirois J. Expression, Regulation, and Promoter Activation of Vanin-2 in Bovine Preovulatory Follicles During the Ovulatory Process Induced by hCG. Biol Reprod [Internet]. 2011 Jul 1 [cited 2025 Apr 22];85(Suppl_1):648–648. Available from: https://dx.doi.org/10.1093/biolreprod/85.s1.648

13. Li Q, Hu S, Wang Y, Deng Y, Yang S, Hu J, et al. mRNA and miRNA Transcriptome Profiling of Granulosa and Theca Layers From Geese Ovarian Follicles Reveals the Crucial Pathways and Interaction Networks for Regulation of Follicle Selection. Front Genet [Internet]. 2019 [cited 2025 Apr 22];10(OCT). Available from: https://pubmed.ncbi.nlm.nih.gov/31708963/

14. Li Z, Liu Y, Ma T, Lv C, Li Y, Duan H, et al. Smart-seq2 Technology Reveals a Novel Mechanism That Zearalenone Inhibits the In Vitro Maturation of Ovine Oocytes by Influencing TNFAIP6 Expression. Toxins 2023, Vol 15, Page 617 [Internet]. 2023 Oct 17 [cited 2025 Apr 22];15(10):617. Available from: https://www.mdpi.com/2072-6651/15/10/617/htm

15. Lazaros LA, Hatzi EG, Xita N V., Makrydimas G V., Kaponis AI, Takenaka A, et al. Aromatase (CYP19) gene variants influence ovarian response to standard gonadotrophin stimulation. J Assist Reprod Genet [Internet]. 2011 Feb [cited 2025 Apr 22];29(2):203. Available from: https://pmc.ncbi.nlm.nih.gov/articles/PMC3270141/

16. Hashemain Z, Amiri-Yekta A, Khosravifar M, Alvandian F, Shahhosseini M, Hosseinkhani S, et al. CYP19A1 Promoters Activity in Human Granulosa Cells: A Comparison between PCOS and Normal Subjects. Cell Journal (Yakhteh) [Internet]. 2022 Apr 1 [cited 2025 Apr 22];24(4):170. Available from: https://pmc.ncbi.nlm.nih.gov/articles/PMC9124446/

17. Kempisty B, Jackowska M, Woźna M, Antosik P, Piotrowska H, Zawierucha P, et al. Expression and Cellular Distribution of INHA and INHB before and after In Vitro Cultivation of Porcine Oocytes Isolated from Follicles of Different Size. J Biomed Biotechnol [Internet]. 2012 [cited 2025 Apr 22];2012:742829. Available from: https://pmc.ncbi.nlm.nih.gov/articles/PMC3511843/

18. Kovacovicova K, Awadova T, Mikel P, Anger M. In vitro maturation of mouse oocytes increases the level of kif11/eg5 on meiosis ii spindles. Biol Reprod [Internet]. 2016 Jul 1 [cited 2025 Apr 22];95(1):1–9. Available from: https://dx.doi.org/10.1095/biolreprod.115.133900

19. Wan X, Zhang Y, Lan M, Pan MH, Tang F, Zhang HL, et al. Meiotic arrest and spindle defects are associated with altered KIF11 expression in porcine oocytes. Environ Mol Mutagen [Internet]. 2018 Dec 1 [cited 2025 Apr 22];59(9):805–12. Available from: https://pubmed.ncbi.nlm.nih.gov/30151839/

20. Yi ZY, Meng TG, Ma XS, Li J, Zhang CH, Ouyang YC, et al. CDC6 regulates both G2/M transition and metaphase-to-anaphase transition during the first meiosis of mouse oocytes. J Cell Physiol [Internet]. 2020 Jul 1 [cited 2025 Apr 22];235(7–8):5541–54. Available from: https://onlinelibrary.wiley.com/doi/full/10.1002/jcp.29469

21. Borlado LR, Méndez J. CDC6: from DNA replication to cell cycle checkpoints and oncogenesis. Carcinogenesis [Internet]. 2008 Feb 1 [cited 2025 Apr 22];29(2):237–43. Available from: https://dx.doi.org/10.1093/carcin/bgm268

22. Almeida CP, Silveira CO, Ferreira EF, Ferreira MC, Oliveira GG, Veloso ES, et al. Pro caspase-3 and cleaved caspase-3 gene and protein expression in human granulosa cells correlate with cos duration, length of infertility and proportion of mature oocytes retrieved. Fertil Steril [Internet]. 2019 Sep [cited 2025 Apr 22];112(3):e183. Available from: https://www.researchgate.net/publication/336050219_Pro_caspase-3_and_cleaved_caspase-3_gene_and_protein_expression_in_human_granulosa_cells_correlate_with_cos_duration_length_of_infertility_and_proportion_of_mature_oocytes_retrieved

23. Zhang C, Zhao L, Leng L, Zhou Q, Zhang S, Gong F, et al. CDCA8 regulates meiotic spindle assembly and chromosome segregation during human oocyte meiosis. Gene. 2020 May 30;741:144495.

24. Uri-Belapolsky S, Miller I, Shaish A, Levi M, Harats D, Ninio-Many L, et al. Interleukin 1-alpha deficiency increases the expression of Follicle-stimulating hormone receptors in granulosa cells. Mol Reprod Dev. 2017 Jun 1;84(6):460–7.

25. Yang H, Pang H, Miao C. Ovarian IL-1α and IL-1β levels are associated with primary ovarian insufficiency. Int J Clin Exp Pathol [Internet]. 2018 [cited 2025 Apr 21];11(9):4711. Available from: https://pmc.ncbi.nlm.nih.gov/articles/PMC6962969/

26. Dhali A, Javvaji PK, Kolte AP, Francis JR, Roy SC, Sejian V. Temporal expression of cumulus cell marker genes during in vitro maturation and oocyte developmental competence. J Assist Reprod Genet [Internet]. 2017 Nov 1 [cited 2025 Apr 22];34(11):1493–500. Available from: https://link.springer.com/article/10.1007/s10815-017-0998-z

27. Xu XH, Jia Y, Zhou X, Xie D, Huang X, Jia L, et al. Downregulation of lysyl oxidase and lysyl oxidase-like protein 2 suppressed the migration and invasion of trophoblasts by activating the TGF-β/collagen pathway in preeclampsia. Exp Mol Med [Internet]. 2019 Feb 1 [cited 2025 Apr 21];51(2). Available from: https://pubmed.ncbi.nlm.nih.gov/30804321/

28. Cano A, Eraso P, Mazón MJ, Portillo F. LOXL2 in Cancer: A Two-Decade Perspective. Int J Mol Sci [Internet]. 2023 Sep 1 [cited 2025 Apr 21];24(18). Available from: https://pubmed.ncbi.nlm.nih.gov/37762708/

29. Bunel A, Nivet AL, Blondin P, Vigneault C, Richard FJ, Sirard MA. Cumulus cell gene expression associated with pre-ovulatory acquisition of developmental competence in bovine oocytes. Reprod Fertil Dev [Internet]. 2014 [cited 2025 Apr 22];26(6):855–65. Available from: https://pubmed.ncbi.nlm.nih.gov/23827322/

30. Guo J, Shi L, Gong X, Jiang M, Yin Y, Zhang X, et al. Oocyte-dependent activation of MTOR in cumulus cells controls the development and survival of cumulus-oocyte complexes. J Cell Sci [Internet]. 2016 [cited 2025 Apr 21];129(16):3091–103. Available from: https://pubmed.ncbi.nlm.nih.gov/27358481/

31. Ouandaogo ZG, Frydman N, Hesters L, Assou S, Haouzi D, Dechaud H, et al. Differences in transcriptomic profiles of human cumulus cells isolated from oocytes at GV, MI and MII stages after in vivo and in vitro oocyte maturation. Human Reproduction [Internet]. 2012 Aug 1 [cited 2025 Apr 22];27(8):2438–47. Available from: https://dx.doi.org/10.1093/humrep/des172

32. Iizuka T, Wakae K, Ono M, Suzuki T, Mizumoto Y, Kitamura K, et al. Activation-induced cytidine deaminase is a possible regulator of cross-talk between oocytes and granulosa cells through GDF-9 and SCF feedback system. Scientific Reports 2021 11:1 [Internet]. 2021 Feb 15 [cited 2025 Apr 22];11(1):1–12. Available from: https://www.nature.com/articles/s41598-021-83529-x

33. Young J, Metay C, Bouligand J, Tou B, Francou B, Maione L, et al. SEMA3A deletion in a family with Kallmann syndrome validates the role of semaphorin 3A in human puberty and olfactory system development. Hum Reprod [Internet]. 2012 [cited 2025 Apr 21];27(5):1460–5. Available from: https://pubmed.ncbi.nlm.nih.gov/22416012/

34. Hanchate NK, Giacobini P, Lhuillier P, Parkash J, Espy C, Fouveaut C, et al. SEMA3A, a gene involved in axonal pathfinding, is mutated in patients with Kallmann syndrome. PLoS Genet [Internet]. 2012 Aug [cited 2025 Apr 21];8(8). Available from: https://pubmed.ncbi.nlm.nih.gov/22927827/

35. Joseph D, Ho SM, Syed V. Hormonal regulation and distinct functions of semaphorin-3B and semaphorin-3F in ovarian cancer. Mol Cancer Ther [Internet]. 2010 [cited 2025 Apr 21];9(2):499–509. Available from: https://pubmed.ncbi.nlm.nih.gov/20124444/

36. Palese M, Ferretti G, Perruolo G, Serafini S, Sirabella R, Marrone V, et al. Semaphorin 3A Increases in the Plasma of Women with Diminished Ovarian Reserve Who Respond Better to Controlled Ovarian Stimulation. Life (Basel) [Internet]. 2024 Mar 1 [cited 2025 Apr 21];14(3). Available from: https://pubmed.ncbi.nlm.nih.gov/38541683/

37. Emery A, Dunning KR, Dinh DT, Akison LK, Robker RL, Russell DL. Dynamic regulation of semaphorin 7A and adhesion receptors in ovarian follicle remodeling and ovulation. Front Cell Dev Biol [Internet]. 2023 [cited 2025 Apr 21];11. Available from: https://pubmed.ncbi.nlm.nih.gov/37941899/

38. Yan W, Zhou S, Shen W, Cheng J, Yuan S, Ye S, et al. Suppression of SEMA6C promotes preantral follicles atresia with decreased cell junctions in mice ovaries. J Cell Physiol [Internet]. 2019 Apr 1 [cited 2025 Apr 21];234(4):4934–43. Available from: https://pubmed.ncbi.nlm.nih.gov/30256425/

39. Regev A, Goldman S, Shalev E. Semaphorin-4D (Sema-4D), the Plexin-B1 ligand, is involved in mouse ovary follicular development. Reprod Biol Endocrinol [Internet]. 2007 Mar 21 [cited 2025 Apr 21];5. Available from: https://pubmed.ncbi.nlm.nih.gov/17376242/
