# Supplementary material for: Tailor-made 3D in vitro maturation of early antral follicles uncovers cumulus-cell transcriptomic driver signature to predict oocyte competence
Source: Front Endocrinol (Lausanne). 2025 Oct 1;16:1629815. doi: 10.3389/fendo.2025.1629815 (PMC12520894; doi:10.3389/fendo.2025.1629815)
Supplement: Supplementary Table 1 — (Excel). The 12 centrality coefficients of each DEG of Network 1(MIIEndpoint- GVStartpoint) (Sheet: N1 MII-GV) and Network 2(GVEndpoint-GVStartpoint) (Sheet: N2 GV-GV) were scored using CytoHUBba. More in detail, they are closeness, degree, MCC, radiality, stress, MCN, DNMC, betweenness, clustering coefficient, eccentricity, bottleneck, and EPC. Network 1(MIIEndpoint- GVStartpoint) and Network 2(GVEndpoint-GVStartpoint) top 10 DEGs defined on each centrality coefficient score (Sheets: Top 10 N1 and N2 respectively). Venn diagram analysis of the top 10 DEGs of Network 1(MIIEndpoint- GVStartpoint) (Sheet: Ranking N1) and Network 2(GVEndpoint-GVStartpoint)(Sheet: Ranking N2) shows DEGs overlapping across the 12 algorithms. DEGs that are in the top 10 in at least 5 of the 6 algorithms are highlighted in bold. (Network1_Normalized) and (Network2_Normalized) include dataset values that have been statistically normalized using the standard score formula. [file DataSheet1.zip › Supplementary datasheets and tables/Supplementary Datasheet 4.docx]

**Supplementary Datasheet 4. Table with number of DEGs included in each analysis process after final filtering criteria**

|  | *Number of Recognized DEGs* | | | |
| --- | --- | --- | --- | --- |
|  | Passed filter (including non-characterized genes) | Passed filter (excluded non – characterized by STRING) | Recognized by Cytoscape plug-in | Recognized by MCODE plug-in |
| *Pairwise 1* | 1386 | 1149 | 1144/1149 | 170/1144 |
| *Pairwise 2* | 1420 | 1209 | 1206/1209 | 259/1206 |

**Number of DEGs included in each analysis process after final filtering criteria.** The table is composed by the amount of DEGs found in each pairwise after excluding non – characterized genes, long non-coding RNAs and genes without a protein annotation from the originally filtered dataset; the number of DEGs recognized by the Cytoscape and MCODE programme, respectively. All data from FEO maturation, parthenogenesis, and IVF experiments represent the mean values from three independent biological replicates. Statistically significant differences (p < 0.05) between EAf and SAf groups are indicated by superscript letters within each column.
